# Altered Chromatin Occupancy of Master Regulators Underlies Evolutionary Divergence in the Transcriptional Landscape of Erythroid Differentiation

**DOI:** 10.1371/journal.pgen.1004890

**Published:** 2014-12-18

**Authors:** Jacob C. Ulirsch, Jessica N. Lacy, Xiuli An, Narla Mohandas, Tarjei S. Mikkelsen, Vijay G. Sankaran

**Affiliations:** 1 Division of Hematology/Oncology, The Manton Center for Orphan Disease Research, Boston Children's Hospital and Department of Pediatric Oncology, Dana-Farber Cancer Institute, Harvard Medical School, Boston, Massachusetts, United States of America; 2 Broad Institute of MIT and Harvard, Cambridge, Massachusetts, United States of America; 3 New York Blood Center, New York, New York, United States of America; 4 Harvard Stem Cell Institute, Cambridge, Massachusetts, United States of America; Stanford University School of Medicine, United States of America

## Abstract

Erythropoiesis is one of the best understood examples of cellular differentiation. Morphologically, erythroid differentiation proceeds in a nearly identical fashion between humans and mice, but recent evidence has shown that networks of gene expression governing this process are divergent between species. We undertook a systematic comparative analysis of six histone modifications and four transcriptional master regulators in primary proerythroblasts and erythroid cell lines to better understand the underlying basis of these transcriptional differences. Our analyses suggest that while chromatin structure across orthologous promoters is strongly conserved, subtle differences are associated with transcriptional divergence between species. Many transcription factor (TF) occupancy sites were poorly conserved across species (∼25% for GATA1, TAL1, and NFE2) but were more conserved between proerythroblasts and cell lines derived from the same species. We found that certain *cis*-regulatory modules co-occupied by GATA1, TAL1, and KLF1 are under strict evolutionary constraint and localize to genes necessary for erythroid cell identity. More generally, we show that conserved TF occupancy sites are indicative of active regulatory regions and strong gene expression that is sustained during maturation. Our results suggest that evolutionary turnover of TF binding sites associates with changes in the underlying chromatin structure, driving transcriptional divergence. We provide examples of how this framework can be applied to understand epigenomic variation in specific regulatory regions, such as the β-globin gene locus. Our findings have important implications for understanding epigenomic changes that mediate variation in cellular differentiation across species, while also providing a valuable resource for studies of hematopoiesis.

## Author Summary

The process whereby blood progenitor cells differentiate into red blood cells, known as erythropoiesis, is very similar between mice and humans. Yet, while studies of this process in mouse have substantially improved our knowledge of human erythropoiesis, recent work has shown a significant divergence in global gene expression across species, suggesting that extrapolation from mouse models to human is not always straightforward. In order to better understand these differences, we have performed a comparative epigenomic analysis of six histone modifications and four master transcription factors. By globally comparing chromatin structure across primary cells and model cell lines in both species, we discovered that while chromatin structure is well conserved at orthologous promoters, subtle changes are predictive of species-specific gene expression. Furthermore, we discovered that the genomic localizations of master transcription factors are poorly conserved, and species-specific losses or gains are associated with changes to the underlying chromatin structure and concomitant gene expression. By using our comparative epigenomics framework, we identified a putative human-specific *cis-*regulatory module that drives expression of human, but not mouse, *GDF15*, a gene implicated in iron homeostasis. Our results provide a resource to aid researchers in interpreting genetic and epigenetic differences between species.

## Introduction

Red blood cell (RBC) production (erythropoiesis) is one of the best understood examples of lineage commitment and cellular differentiation [1]–[4]. This process begins as multipotent hematopoietic stem cells (HSCs) differentiate into lineage committed erythroid progenitors, losing multipotency in intermediate progenitor cell populations. Early erythroid progenitors then differentiate into morphologically distinct early erythroid precursors, termed proerythroblasts (ProEs). The ProEs subsequently undergo terminal erythroid differentiation into mature RBCs that enucleate, contain a significant concentration of hemoglobin, and have highly elastic cytoskeletons [3]. This differentiation process is governed by a number of transcription factors (TFs) that dynamically coordinate a complex transcriptional gene regulatory network (GRN). Importantly, much of our knowledge of this GRN has been derived from mouse models of erythropoiesis [1]–[3]. Extrapolation from mouse models of terminal erythroid differentiation to humans has historically been straightforward, grounded in the nearly identical morphology of mature RBCs and their precursors between species [4]–[6]. While there are many well-known examples of species-specific differences in erythroid GRNs, such as developmental variation of β-like globin gene expression, the divergent role of BCL11A during developmental hemoglobin switching, and differences in *cis-*regulatory modules (CRMs) regulating *GATA1* transcription [7]–[9], a marked global divergence in the expression profiles of the erythroid lineage was only recently described by systematic comparative analyses of human and murine erythroid transcriptomes [10], [11].

Indeed, these recent studies independently identified a large global divergence in temporal patterns of gene expression between human and mouse at critical, canonical stages of terminal erythroid differentiation [10],[11]. While many erythroid specific pathways and genes were generally conserved, such as the heme biosynthetic pathway, cytoskeletal proteins, and master TFs of erythropoiesis (e.g. *GATA1*, *NFE2*, and *KLF1*), significant differences in the timing and expression levels of certain constituent genes were observed (e.g. *TAL1*) [10]. In some pathways, such as the mitogen-associated protein kinase (*MAPK*) pathway, gene profiles were markedly divergent between species during differentiation [11]. These differences have many important implications for integrating the extensive information on erythropoiesis gained from mouse models to better understand human erythropoiesis and how this process goes awry in human disease. For example, congenital dyserythropoietic anemia type II (CDA II) is caused by recessive mutations in *SEC23B*, but the phenotype could not be recapitulated in mouse models [12]–[14]. The expression of *SEC23A*, a *SEC23B* paralog, varied between mice and humans, suggesting a reason for these divergent phenotypes. Moreover, these expression differences were accompanied by variation in TF occupancy proximal to *SEC23A* in erythroid cell lines suggesting that species-specific differences in transcription may be due to evolutionary divergence in TF occupancy and the epigenome [10]. However, the conservation or divergence of chromatin structure and TF occupancy between human and murine erythropoiesis has only been characterized in a few specific regions, and, to the best of our knowledge, we are not aware of any studies that measure the extent to which there is divergence or conservation across the genome [7], [12]. We have therefore undertaken a comparative epigenomic study to systematically analyze the global conservation of histone modifications and master transcriptional regulators necessary for erythroid differentiation. We map these epigenomic marks in both human and murine primary ProEs as well as in the model erythroid cell lines of human and mouse, K562 and G1E/G1E-ER (herein referred to as G1E), respectively. We compare these marks in the context of orthologous genes as well as across conserved regions of both genomes. Finally, we integrate high-quality stage-matched gene expression profiling (RNA-seq) of each cell type to investigate functional intra- and inter-species differences across the epigenome.

Our results suggest that chromatin structure and function is generally well conserved both between species and in erythroid cell models, although certain modifications are under greater constraint than others. In contrast, only ∼25% of the occupancy sites of most TFs are conserved between species, whereas we observed a 2-fold increase in conservation rates for erythroid cell models, validating K562 and G1E cell lines as species-specific model systems for studying such TFs. Nevertheless, we find that CRMs co-occupied by KLF1, GATA1, and TAL1 are significantly more conserved than any lower order combination of these factors and are strictly localized near highly-expressed genes that play a key role in defining erythroid cell state, suggesting that these regions are under strong evolutionary constraint to regulate common features of mammalian erythropoiesis. Moreover, although we show that chromatin structure is largely conserved between similar developmental cell-types across species, subtle changes in chromatin structure are associated with transcriptional divergence. Based on multiple lines of evidence, we suggest that evolutionary changes in transcription are partially driven by large-scale loss or gain of master TF occupancy that associate with changes to the underlying chromatin structure. In addition, these results provide a resource that can aid in translating findings from mouse erythropoiesis to the analogous process in humans.

## Results

### Conservation of histone modifications and TF occupancy between intra- and inter-species cell types at protein-coding genes

For each species, we compiled chromatin immunoprecipitation high-throughput sequencing (ChIP-seq) data sets of histone modifications (H3K4me1, H3K4me2, H3K4me3, H3K9ac, H3K27me3, H3K36me3) and master TFs of erythropoiesis (GATA1, TAL1, KLF1, NFE2) at the ProE stage of erythroid differentiation (S1 and S2 Table) [15]–[25]. The vast majority of ChIP-seq data was available at the ProE stage, and this is known to be an important time point where a variety of epigenetic changes occur to mediate alterations in the transcriptional landscape [19], [20], [26], [27]. Additionally, we compiled and analyzed ChIP-seq data from erythroid cell lines, K562 (human leukemia cell line) and G1E/G1E-ER (mouse erythroid cell lines that are derived from *Gata1*-null erythroid cells containing an estrogen-inducible Gata1 transgene; herein G1E).

**Figure 1:**
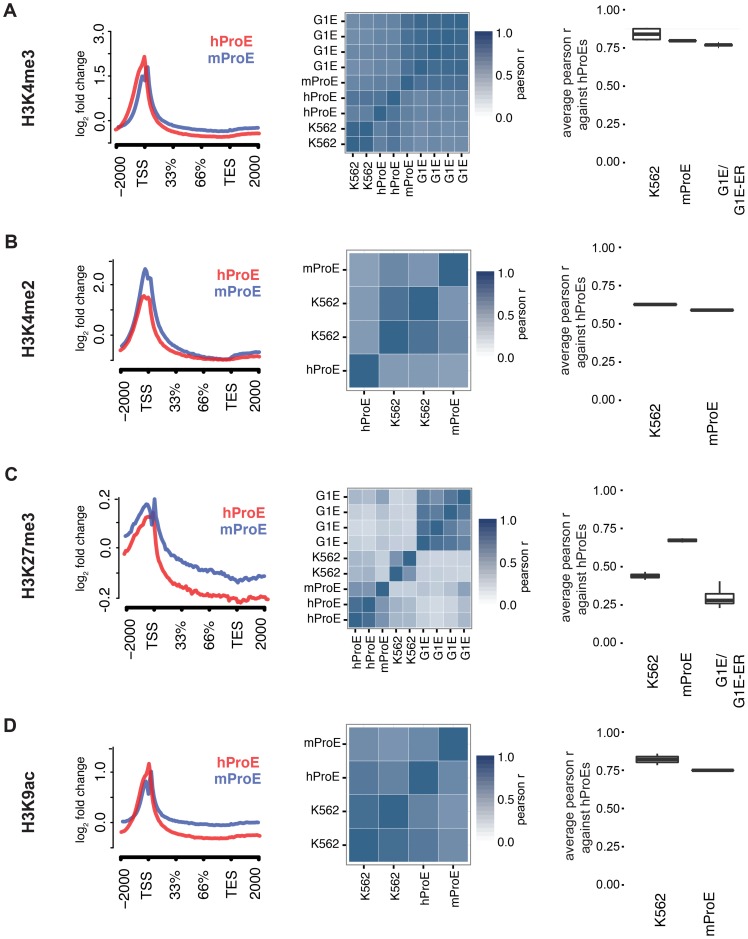
Inter- and intra-species conservation of histone modifications in orthologous promoters. A)–D) *Left*: Average curves of normalized log_2_ fold changes across 15506 orthologous genes for each histone mark. The size of each gene is normalized in order to represent the average shape of histone mark intensity. *Middle*: Heatmaps are clustered by the similarity of the pearson r for histone mark intensities between all cell-types. hProEs are CD71+, mProEs are Ter119+, K562 cells are a human erythroid cell line, and G1E/G1E-ER cells are a mouse erythroid cell line. Replicates are included as independent observations. *Right*: For each category shown (e.g. mProEs, K562, and G1E/G1E-ER), the average pearson correlation between each replicate of that type and each replicate of human ProEs is presented as boxplots. *Abbreviations used*: hProE, human pro-erythroblast; mProE, mouse pro-erythroblast.

We initially leveraged the compiled data to investigate local chromatin structure and TF occupancy across 15,506 orthologous gene bodies with a one-to-one mapping because local chromatin structure is largely indicative of transcription status and interspecies TF occupancy differences [28], [29]. Overall, our observations are concordant with prior data suggesting that the functions of histone modifications, indicated by similar histone intensity profiles and the percent of genes present near each, are well conserved between humans and mice (Fig. 1 left panel, S1 & S2 Fig.) [30]. For example, the signal intensity of H3K4me3, generally regarded as a mark of transcriptional activation, was present in ∼50% of genes in both species and its intensity peaked at the transcription start site TSS (Fig. 1A), while the pattern of H3K27me3, a mark of transcriptional repression, was conserved overall but was present in a lower number of genes (∼20%) (Fig. 1C, S2 Fig.).

When we compared TF (GATA1, KLF1, TAL1, NFE2) occupancy profiles across gene-bodies identical to the above analysis of histone modifications, we discovered that for each TF, normalized occupancy intensities varied significantly more between species than histone modifications (S3 Fig.). One hypothesis for this observation is that certain TFs such as TAL1 are not as abundant or active in mouse versus human ProEs, although our expression data suggests that *TAL1* is highly abundant at this stage in both species [11]. More likely hypotheses are that technical differences in ChIP protocol between labs explain most of the observed difference or that the differences are truly biological. A thorough analysis supporting these alternative hypotheses is detailed in the materials and methods.

To quantify the potential divergence in chromatin structure and TF occupancy, we compared relative histone modification intensity across the proximal promoter regions of a smaller set of 6596 orthologous genes with canonical transcripts in both species. We included erythroid cell lines in this analysis and used human ProEs as the primary cell type against which all others were compared to assess inter- and intra-species conservation of promoter epigenetic structure. Generally, histone promoter modifications were highly conserved between the two species and replicate experiments were highly correlated (middle and right columns of Fig. 1, S3 Table). H3K4me3, H3K4me2, and H3K9ac were highly conserved between all intra- and inter-species cell types, although modifications in K562 were most correlated with those in human ProEs (Fig. 1A,B,D). Interestingly, H3K27me3 was more conserved in mouse ProEs than K562 cells (Fig. 1C). The observed divergence of H3K27me3 in the leukemic K562 cell line is consistent with the fact that H3K27me3 modifications are frequently dysregulated during oncogenesis [31]. H3K36me3 and H3K4me1 were moderately conserved and more strongly correlated between cell types than between species, although these marks show the weakest enrichment at the TSS (S1 Fig. and S3 Table).

When we compared TF occupancy intensity, GATA1 and TAL1 intensity in human ProEs was moderately correlated with that of K562 cells, but not with mouse ProEs or G1E cells (S4 Fig.). In contrast, KLF1 and NFE2 occupancy was weakly to moderately correlated across all cell-types (S4 Fig.). Importantly, K562 cells proved a significantly better model of promoter TF activity in comparison with primary human erythroid promoters than mouse ProEs.

Comparing erythroid cell lines directly, the two classes of cell types showed the weakest correlation, consistent with their respective derivation from primary cells (S3 Table). G1E cells showed similar correlations to mouse ProEs and were moderately to strongly correlated with mouse ProEs across all modifications (S3 Table). Interestingly, these results suggest that active promoters, marked by H3K4me3, H3K4me2, and H3K9ac, are under strict evolutionary constraint and that conservation of these histone modifications is necessary for transcription that defines cell state across species. Overall, these data add to the increasing evidence that inter-species epigenetic differences are larger than intra-species differences – at least for cells that take on a similar global cellular state [29].

### Global divergence of TF occupancy across species

Promoters are only one piece of the total regulatory landscape, so we extended our analysis and performed a global cross-species comparison of chromatin structure and master TF occupancy to better understand patterns in epigenomic evolution. We investigated conservation of global occupancy patterns for all four master regulators of erythropoiesis. Briefly, we derived robust TF occupancy peaks and lifted narrow summits from the mouse genome to the human genome to assess conservation. We note that in this section, when we discuss conservation, we are primarily referring to “conservation of TF occupancy sites” between species or cell types.

**Figure 2:**
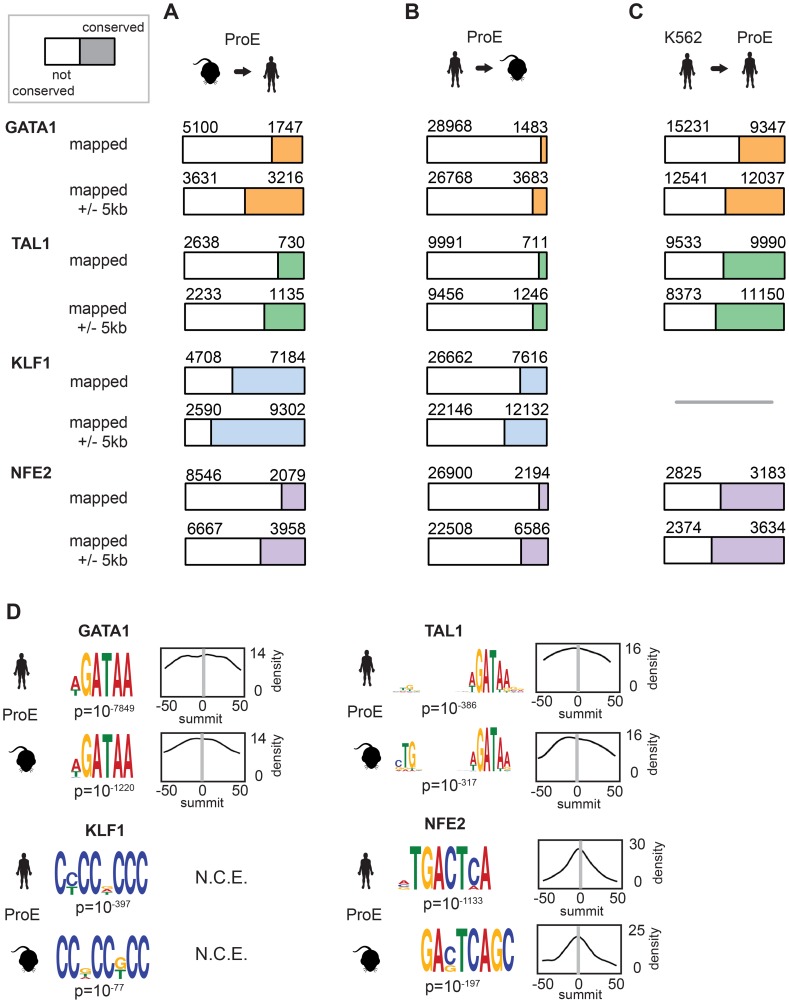
Divergence of transcription factor occupancy sites between human and mouse. A) For each TF (GATA1, TAL1, KLF1, and NFE2), a narrow occupancy site (summit +/- 50 bp) was mapped from mouse ProEs (mm10) to hg19 coordinates using the UCSC liftOver tool and intersected with corresponding peaks in human ProEs. To investigate “compensatory” new occupancy sites in hg19, narrow peaks that were mapped were expanded 5000 bps in each direction and the overlap was recomputed. The denominator (blue, orange, green, or purple plus white) represented the total number of mapped peaks from mouse ProEs to hg19, and the numerator (blue, orange, green, or purple) represented the total number of these mapped peaks that overlap with peaks in human ProEs, referred to as “conserved” occupancy in the main text. B) Similar to A), except that peaks in human ProEs (hg19) were mapped to peaks in mouse ProEs (mm10). Please note that the total number of peaks mapped from mouse to human is smaller than the number mapped from human to mouse. C) Similar to B), except that peaks in the K562 erythroid cell line were directly intersected with peaks in human ProEs. KLF1 data was not available for K562 cells so no overlap was computed. D) For each peak in human and mouse ProEs, MeME-Chip was used to recover canonical motifs for each TF (GATA1, TAL1, KLF1, and NFE2) in a small window (+/- 50 bps) around the summit of each peak. The probability density for each motif is shown across the region (for example, a density of 14 is a probability of 0.14 on a 0 to 1 scale). *Abbreviations used*: ProEs, pro-erythroblasts; N.C.E, no central enrichment.

During the 75 million years of evolution separating the two species, ∼75% of master regulator (GATA1, TAL1, and NFE2) occupancy sites were lost between humans and mice (Fig. 2A, “mapped”). In stark contrast, greater than 60% of KLF1 occupancy peaks were conserved between species. Interestingly, we observed that in ∼25% of lost TF peaks, new human-specific occupancy sites were created for each TF in nearby regions (+/- 5 kbs, henceforth known as “compensatory” occupancy sites), a phenomenon that has been described between human and mouse hepatocytes, adipocytes, and closely related *Drosophila* species (Fig. 2A, “mapped +/- 5 kb”) [32]–[34]. Nevertheless, although we observed that large numbers of TF occupancy sites were lost between species, each master regulator is far more conserved than expected by chance (p<10^−5^ for each, permutation test) and canonical TF binding motifs were nearly identical for each TF across species (Fig. 2D). These findings suggest that the exact genomic location of each TF occupancy site may not be as functionally important as its presence in a broader genomic region and highlight the idea that some presumed cis-regulatory modules (CRMs) may have at most small functional effects.

We also considered the differences in peaks called between species by mapping human peaks to mouse peaks (Fig. 2B). We observed a similar ranking of TF conservation, although the percentages were overall much lower, reflective of the greater number of occupancy sites called in humans. These percentages represent a lower bound on the true percentage of conserved peaks, while those shown in Fig. 2B are a better estimate of the true conservation of TF occupancy rate. As a sensitivity analysis, we investigated conservation of only the strongest 25% of TF occupancy peaks, providing an upper bound on the conservation estimate for each TF (S5 Fig.).

In juxtaposition to mouse sites, TF occupancy sites in K562 cells and human ProEs were highly concordant: ∼50% of occupancy sites were identical, and only a small percentage of compensatory peaks were observed (Fig. 2C). Importantly, the upper bound of the conservation estimates for human and mouse ProEs are still below the standard estimates for human ProEs and K562 cells. Overall, these data suggest that while select master regulators, such as KLF1, are under strong constraint, most master regulators, including GATA1, TAL1, and NFE2, are under weak to moderate constraint. Second, these data suggest that although TF occupancy sites are often lost during evolution, functional effects from these losses are partially buffered by the emergence of compensatory occupancy sites, a possibility that we validate in subsequent analyses. Our findings, both globally across the genome and in promoter regions, support the idea that intra-species TF occupancy is more conserved than inter-species TF occupancy.

### Regulatory modules co-occupied by KLF1, GATA1, and TAL1 are constrained

**Figure 3:**
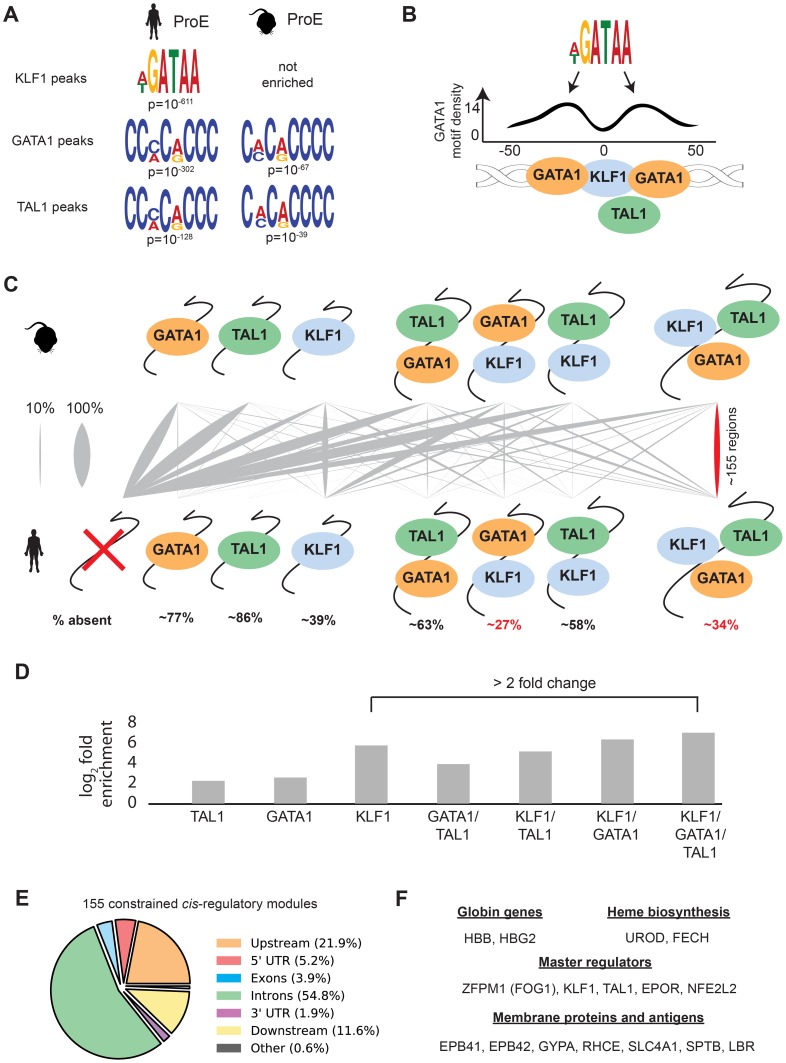
Combinatorial occupancy patterns of transcription factors are strongly conserved. A) In human ProE KLF1 peaks (summit +/- 50 bp), GATA1 motifs were significantly enriched. In human and mouse ProE GATA1 and TAL1 peaks, certain KLF1 motifs were significantly enriched. B) Enrichment of GATA1 motifs identified in A) at non-random distances from the summit of KLF1 occupancy sites reveals that GATA1 and GATA1/TAL1 occupancy may often be found in KLF1-occupied regions. Scales are identical to Fig. 2D. C) For each combinatorial group, the proportion (x_i_/n) that is mapped from mouse to human is represented as grey lines, where a thicker grey line indicates that a larger proportion. Most regions in mouse are lost in human, but certain combinations (GATA1 and KLF1; GATA1, KLF1, and TAL1) are significantly more conserved than others (p<10^−16^). % absent is (1- number of mapped regions with no TF occupancy). D) Fold enrichment of combinatorial TF occupancy overlap (observed divided by expected) between species calculated with GAT. E) Genomic localization of the 155 conserved GATA1, KLF1, and TAL1 co-occupied regions. F) A large number of canonical erythroid genes are assigned by proximity to these 155 regions, suggesting that these are co-occupied regions are functionally conserved regulators. Abbreviations used: ProEs, pro-erythroblasts; GAT, Genomic Association Tester.

In contrast to GATA1, TAL1, and NFE2 binding motifs, KLF1 motifs (SP1 or CACC) were not centrally enriched around the summit of KLF1 occupancy sites (although enrichment for the canonical motifs were observed across the entire peak). A thorough investigation of enriched motifs in KLF1 occupancy sites revealed that GATA1 and GATA1/TAL1 motifs were proximally, but not centrally, enriched in KLF1 peaks in human (Fig. 2D, Fig. 3A–B, S1–S8 Dataset). Furthermore, KLF1 motifs were recovered in both GATA1 and TAL1 peaks for both human and mouse, suggesting that regions co-occupied by KLF1, GATA1, and TAL1 are true CRMs under stricter evolutionary constraint than regions occupied by each factor alone (Fig. 3A).

To address this hypothesis, we mapped combinatorial occupancy regions of these three factors from the mouse to the human genome (S6A Fig.). We discovered that when one or more TF overlapped, the region was more likely to be conserved (p<10^−5^ for each, Fig. 3D). Confirming our hypothesis, ∼35% of regions co-occupied by GATA1, KLF1, and TAL1 in mice were also co-occupied by all three factors in humans, a result far more likely than by chance and a higher rate of conservation than any other grouping (p<10^−5^, Fig. 3C–D). Our observation that certain co-occupied TFs are more conserved than individual TFs is consistent with similar findings across closely related mammalian species [35].

This result suggests that CRMs co-occupied by all three master regulators are important for the regulation of highly conserved processes during erythropoiesis. Confirming this, we found that the majority of these regions localize to and may act as enhancer elements for a number of genes important for erythropoiesis including: β-globin, heme biosynthetic enzymes, red cell membrane and surface proteins, and master regulators of erythropoiesis (Fig. 3E–F). Additionally, the importance to erythropoiesis of many of the genes proximal to these constrained enhancers is unknown, providing a short list of potential new regulators of erythropoiesis under strict evolutionary constraint (S4 Table). Overall, these observations validate the full extent to which KLF1, in conjunction with GATA1 and TAL1, regulates many facets of mouse and human erythropoiesis [18], [36], [37].

### TF alterations associate with chromatin state changes and species-specific gene expression

**Figure 4:**
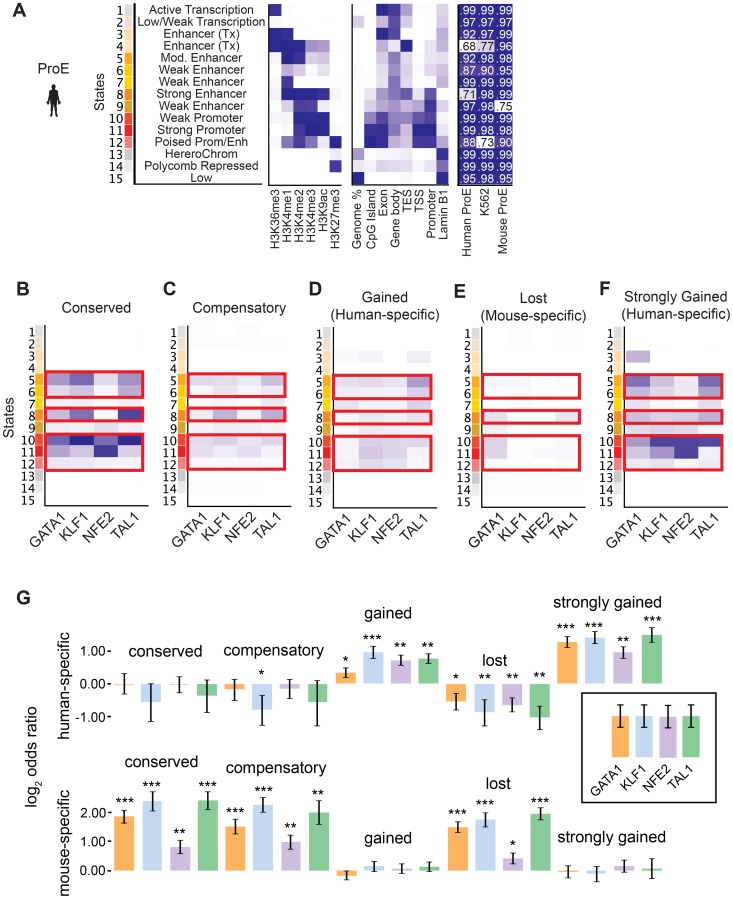
Species-specific and conserved transcription factor occupancy associates with histone modifications. A) Emission states of chromatin structure from ChromHMM. Darker blues correspond to higher percentage of representation in a specific state. In addition to chromatin modifications, states are compared to known genomic regions where darker blues correspond to increased fold enrichment versus expected. Each state was highly correlated (pearson correlation values shown) with at least one state in models learned separately in human ProEs, mouse ProEs, and K562 cells. B)–F) Chromatin state enriched in B) TF occupancy sites conserved between human and mouse, C) compensatory TF occupancy sites that are human-specific and proximal (+/- 5kb) to a lost TF occupancy site during evolution, D) human-specific occupancy sites that are gained during evolution, E) mouse-specific occupancy sites that are lost during evolution, and F) top 10% of human occupancy sites based upon mapped reads. Probability of enrichment is scaled across all peak regions. Overall, promoter and enhancer regulatory regions are decreasingly enriched for conserved, compensatory and human-specific, and finally mouse-specific occupancy sites. G) Human-specific genes are defined as the top 10% of differentially expressed genes in human ProEs and mouse-specific genes are defined as the top 10% of differentially expressed genes in mouse ProEs. For each category of TF occupancy, log2 odds ratios for the frequency of TF occupancy in human- or mouse-specific genes are compared to this frequency in the remaining 90% of genes. *Abbreviations used*: ProEs, pro-erythroblasts.

Considering the divergence in TF occupancy sites between species, we investigated the extent to which underlying chromatin structure was associated with the observed loss or gain of different master TF occupancy sites. We undertook a comprehensive approach to annotate all regions of the genome by utilizing a hidden Markov model (HMM) to infer 15 biologically meaningful chromatin “states” in ProEs and for K562 cells, each comprised of multiple different histone modifications with varying “strengths” (i.e. frequencies) for every 200 bp region across both genomes (Fig. 4A, S7 Fig., S8 Fig., S9 Fig., S5 Table, see materials and methods for details) [38].

To facilitate comparisons across species, master regulator occupancy sites were grouped according to conservation. “Conserved” occupancy sites were defined as occupancy sites present in both mouse and human orthologous genomic regions, “lost” or “mouse-specific” were present in mouse but not in human, “compensatory” were gained in human proximal to a lost occupancy site, “gained” or “human-specific” were present in human but not in mouse, and “strongly gained” sites are the top 10% of human-specific occupancy sites.

We observed that conserved occupancy sites were most significantly enriched for active chromatin states that include strong enhancers and promoters (state 5, 6, 8, 10, 11, 12) (Fig. 4B). These states were also enriched at compensatory, gained, and strongly gained (human-specific) occupancy sites, but not at lost (mouse-specific) occupancy sites (Fig. 4C–F, p<10^−5^ for each comparison versus lost). Importantly, we observed that active regulatory states are more enriched for both conserved and strongly gained TF occupancy sites than for compensatory or all gained sites (Mann-Whitney test, p<10^−5^), but we did not observe a difference in enrichment between conserved and strongly gained sites (p = 0.56) or between compensatory and gained sites (p = 0.54). This pattern of regulatory chromatin enrichment was replicated in K562 cells, which themselves appear to have a similar chromatin state to human ProEs, supporting the functionality of these definitions (S10A–E,G Fig.).

In mouse ProEs, conserved TF occupancy sites were also enriched at active chromatin states and mouse-specific occupancy sites, while no enrichment was observed at strong human-specific sites, suggesting first that conserved TF occupancy sites are functional and preserve strong regulatory chromatin structure across millions of years of evolution (S10F Fig.). However, it also suggests that there is a dramatic change in chromatin structure at orthologous human- and mouse-specific TF occupancy sites (4F Fig.).

To determine if functional changes in transcription are associated with alterations in TF occupancy during the course of evolution, we investigated occupancy near species-specific genes (see materials and methods). We discovered that human-specific genes are significantly enriched for human-specific (gained and strongly gained) TF occupancy (Fig. 4G). Corresponding to this observation, mouse-specific genes are significant enriched for mouse-specific (lost) TF occupancy sites. Surprisingly, these genes are also enriched for conserved and compensatory occupancy sites, a finding that we investigate more thoroughly below.

Although the direction of causality is difficult to determine, we suggest that master TFs partially drive epigenomic evolution at orthologous genomic regions by mediating changes to the underlying chromatin structure. Indeed, it has been shown in corresponding null cell lines that the addition of master TFs, such as GATA1, can remodel chromatin structure to increase transcription of certain genes, but our results suggest that master regulators play a far more important global role in chromatin remodeling during evolution [39]. Alternatively, *de novo* chromatin remodeling may impair the ability of TF complexes to bind, resulting in the transcriptional changes observed.

### Evolutionary divergence of the epigenome drives transcriptional change

We sought to understand the functional consequences of the observed epigenomic differences by quantifying the extent to which changes in chromatin structure and master regulator occupancy explain transcriptional divergence between species during terminal erythroid differentiation. We verified, using time-series RNA-seq data of gene expression, that intra-species transcription is indeed more conserved than inter-species transcription (S11 Fig.) [11]. For example, the gene expression profiles of late stage human OrthEs are more similar to early stage human ProEs than they are to mouse OrthEs (S11 Fig.). We observe that the matching early progenitor states (ProEs and BasoEs) are more similar to their species-specific erythroid cell model, K562 or G1E, than to corresponding stages across species (S11 Fig.).

**Figure 5:**
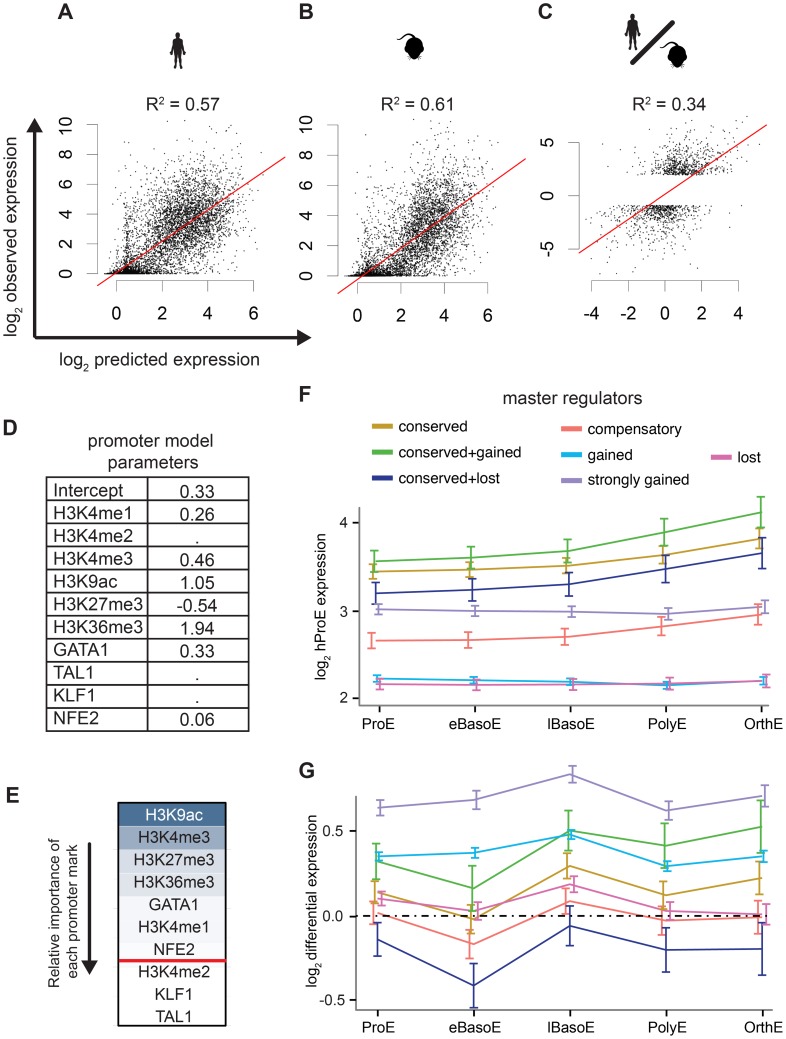
Predictive models of gene expression across species. A)–C) The y-axis is observed values and the x-axis is predicted values. R^2^ values are reported from each consensus model where the lambda “penalty” value is cross-validated ten times and chosen as 1 standard error below the best to prevent over fitting. A) Consensus model predictions from mouse ProE epigenomic marks predict mouse ProE gene expression. B) Consensus model predictions from human ProE epigenomic marks predict human ProE gene expression. C) Applying the consensus model to the difference of human and mouse epigenomic marks is predictive of differences in transcription between the species. D) Coefficients for each retained variable for consensus model (top of A)). E) Relative importance of each epigenomic mark in the consensus model based upon scaled coefficients in the consensus model (un-scaled coefficients are shown in D). F) Gene expression patterns during terminal erythroid differentiation based upon proximity to TF occupancy sites for varying TF occupancy conservation (defined in Fig. 4B–F) across all TFs. G) Similar to F), except shown for changes in gene expression between the two species (human-specific genes correspond to positive values). *Abbreviations used*: ProEs, pro-erythroblasts; eBasoE, early basophilic erythroblasts; BasoE, basophilic erythroblasts; lBasoE, late basophilic erythroblasts; PolyE, polychromatic erythroblasts; OrthE, orthochromatic erythroblasts; FPKM, fragments of aligned reads per kilobase of transcript per million mapped reads.

Intensity of epigenomic marks around TSSs has been shown to explain up to ∼50% of gene expression, providing a simple framework to globally investigate species-specific differences in transcription [40]. We derived a naïve predictive model of transcription in ProEs based upon total epigenomic mark intensity in promoter regions using linear regression with an L_1_ penalty. Our derived models of ProE gene expression learned across both species using six histone modifications and four TFs performed well: without over fitting, these models are able to explain between 58% and 61% of the variation in gene expression for each species based upon the coefficient of determination (R^2^; Fig. 5A-B,D). Models learned independently on each species resulted in similar parameters and were unable to perform better, confirming that transcriptional “rules” are strongly conserved across species (S6 Table). This model remained highly predictive throughout terminal erythroid differentiation, providing further evidence that most epigenetic modifications are dynamically determined at the ProE stage (S6 Table). Interestingly, in this model, chromatin modifications and not TF-occupancy, were most predictive of gene expression (H3K9ac, H3K4me3, H3K27me3, H3K36me3, and GATA1 in order of importance, Fig. 5E).

Having confirmed the biological significance of our model, we applied it to model differences in gene expression between species based upon changes in epigenetic marks. Utilizing this approach, we are able to explain 18% of the changes in gene expression between species. Considering that most genes are not differentially expressed between species, we applied our model to only species-specific expressed genes (see materials and methods). In this case, we are able to explain 34% of the variation in gene expression between species based solely upon promoter epigenetic mark (Fig. 5C).

### TF occupancy site conservation is associated with gene expression

Although our promoter model was highly predictive and elucidated functional biological divergence, we further address the possibility that transcriptional changes are also associated with TF occupancy without restricting our analysis to only promoter regions. Specifically, we investigated the hypothesis that the evolutionary loss or gain of TF occupancy at CRMs is indicative of changes in nearby gene expression. We summarized time-series gene expression profiles for each of the categories of TF conservation that we defined previously (conserved, gained and strongly gained (human-specific), lost (mouse-specific), compensatory, and two additional subcategories of conserved occupancy sites, Fig. 5F and S12 Fig.).

Across all TFs, genes proximally occupied by at least one conserved TF were expressed at significantly higher levels across all cell states, from ProE to orthochromatic erythroblasts (OrthEs) (Fig. 5F and S12 Fig.). The sequential ordering by differential gene expression of groups associated with different TF occupancy (conserved > strongly gained > compensatory > gained > lost) is identical to the ordering of these groups based upon their association with active regulatory states. Furthermore, limited evidence suggests that while gene expression is most similar between groups at terminal stages, loss or gain of master TF occupancy may affect the timing of gene expression, resulting in subtle differences in expression during differentiation (S12 Fig.).

Applying this method to cross species differences in expression, we discover that genes proximally occupied by a strongly gained TF site show human-specific expression during terminal erythroid differentiation (Fig. 5G, S13 Fig.). Interestingly, the genes that show the strongest mouse-specific expression are, first, occupied by one or more TF sites that are conserved across species but, second, have one or more mouse-specific TF occupancy site. These findings suggest that the gains and losses of TF occupancy sites are associated with changes in transcription across species. Moreover, we remark on the observation that even though a single TF may be conserved across species, changes in the occupancy of other TFs at nearby regions may have large functional effects, similar to previously reported results [35].

As a general principle, our observations show that conserved TF occupancy across species is associated with both strong gene expression and active regulatory states. Indeed, while this principle has been shown for conserved GATA1 DNA binding motifs on a small scale, we have confirmed this principle for multiple master regulators with biochemical data across both genomes [41]. Slightly attenuated patterns are observed for species-specific TF occupancy, while orthologous genomic regions of lost TF occupancy show little enrichment for active regulatory states and are indicative of low gene expression. Furthermore, these data suggest that not all species-specific occupancy sites have an immediately observable function: only strong human-specific occupancy was clearly associated with actively transcribed genes.

### Interrogation of specific loci between species

To illustrate specific features of epigenomic conservation and divergence during cellular differentiation, we examined a few well-known regulatory regions involved in erythropoiesis, leveraging our framework to gain further insight into the physiological relevance of these differences. We first describe two regions of general epigenomic conservation with subtle, but important, differences.

**Figure 6:**
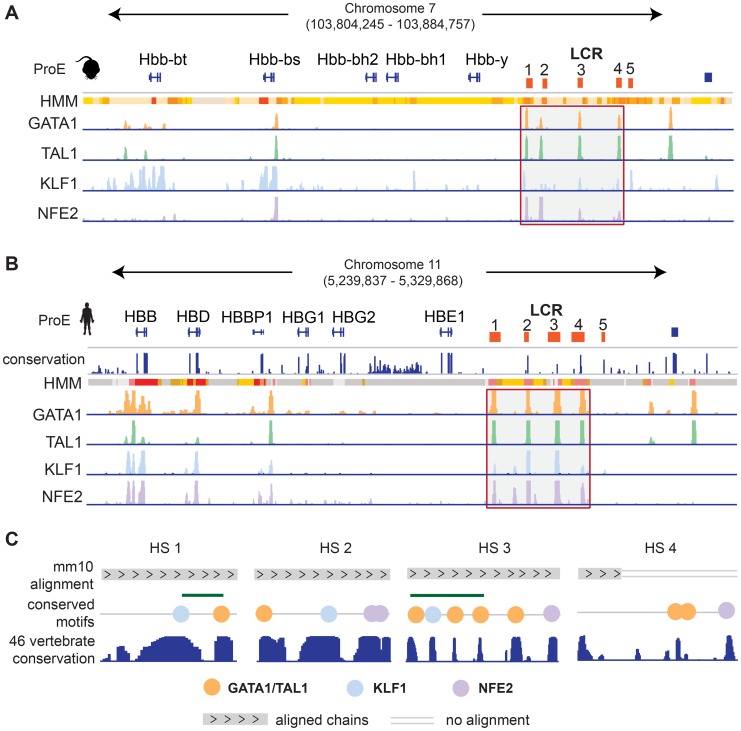
Conservation of the epigenome at the locus control region. A)-B) For both A) mouse and B) human ProEs, chromatin states derived from ChromHMM and TF occupancy profiles for GATA1, TAL1, KLF1, and NFE2 are shown at the globin genes and the locus control region (LCR). The LCR is comprised of 5 HSs. The 1^st^–4^th^ HSs are highlighted for both mouse and human. Chromatin state legend is provided in Fig. 7B. C) Zoomed in views of the 1^st^–4^th^ HSs are shown. Chains of the mouse alignment from Multiz 46-verterbrate alignment are shown as well as PhastCons scores of nucleotide conservation across the 46-vertebrate alignment. HS 1 and HS 3 are both occupied by GATA1, TAL1, and KLF1 strongly conserved elements and canonical occupancy sites present in both the mouse and human genomes under strong selective pressure based upon the 46-vertebrate genome conservation. Only part of the genome underlying the GATA1/TAL1 occupancy site in HS 4 can be mapped in mouse including two canonical GATA1/TAL1 motifs that have been identified as under strong selective pressure in other vertebrates. *Abbreviations used: ProEs, pro-erythroblasts; LCR, locus control region; HS, hypersensitive site.*

We investigated the well studied locus control region (LCR) of the developmentally regulated β-like globin genes [42]. In both species, the LCR consists of 5 closely spaced regulatory regions, termed hypersensitive sites (HSs), directly upstream of the embryonic and adult β-like globin genes (Fig. 6). Each region in the LCR has been shown to loop to developmental stage-specific β-like globin genes to promote high-level gene expression [43], [44]. Here, we investigate the conservation of TF-occupancy and chromatin state assignment at the first four HSs.

We observe that the TF-occupancy profiles of these HSs are strongly conserved across species (Fig. 6A–B). In particular, GATA1 and TAL1 bind strongly to each HS. Canonical DNA motifs conserved across 46 vertebrates and present in both human and mouse genomes can be identified (Fig. 6C). Interestingly, we observe that the 1^st^ and 3^rd^ HSs are two of the highly constrained CRMs co-occupied by GATA1, TAL1, and KLF1, confirming that these HSs are under strict evolutionary constraint. We observe stronger KLF1 intensity at HS1 compared to HS2 in human, but stronger KLF1 intensity at HS2 versus HS1 in mouse. Additionally, we observe increased NFE2 occupancy at HS3 in humans compared to the same region in mouse. Furthermore, the first four HSs in the LCR are associated with strong/poised enhancer states in humans whereas they are associated with strong/weak enhancer states in mice. Finally, although GATA1 and TAL1 occupy HS4 across species, the specific binding sites in this HS appear to be different for human and mouse (Fig. 6C). Overall, chromatin structure and TF occupancy at the β-globin LCR is largely conserved, but subtle differences may have effects on stage-specific transcriptional patterns.

We next turned our focus to the large 2^nd^ intron of *BCL11A* containing an erythroid specific enhancer that, when disrupted in mouse cell lines, reduces *BCL11A* transcription and has been suggested to underlie common genetic variation of this key globin switching factor [45], [46]. This enhancer is occupied by GATA1 and TAL1 in humans and contains a GATA1/TAL1 motif that is partially disrupted by the minor allele of the common polymorphism, rs142707 (degenerative TAL1 motif is CAT for the wildtype and CAG for the minor allele, S14A–C Fig.). Although this binding motif is conserved across species, the guanine minor allele in humans is the ancestral allele present in other primates and mice (S14C Fig.). We observe that GATA1 and TAL1 are also enriched at this site in mouse ProEs, suggestive of a conserved function for this enhancer element (S14A Fig.). Nevertheless, we more broadly observe divergent patterns of TF occupancy, histone modifications, and gene expression across species, suggestive of functional differences at this locus (S14A–B,D Fig.). This finding emphasizes that caution must be applied when investigating and interpreting results from single TF occupancy or HS site data alone rather than a comprehensive approach that includes multiple factors and histone modifications across a broader region.

Next, we focus on two examples that show substantial divergence across the epigenome. Recessive mutations in SEC23B have been implicated in congenital dyserythropoietic anemia type II (CDA II), but an erythroid phenotype could not be recapitulated in mouse models [12]–[14]. One hypothesis for this observation is that while SEC23A is not expressed in similar human cell-types, Sec23a is expressed in mouse and is functionally able to compensate for the absence of Sec23b, resulting in the absence of a phenotype in *Sec23b* knockout mice [10], [47]. We therefore investigated these potential differences in transcriptional regulation.

**Figure 7:**
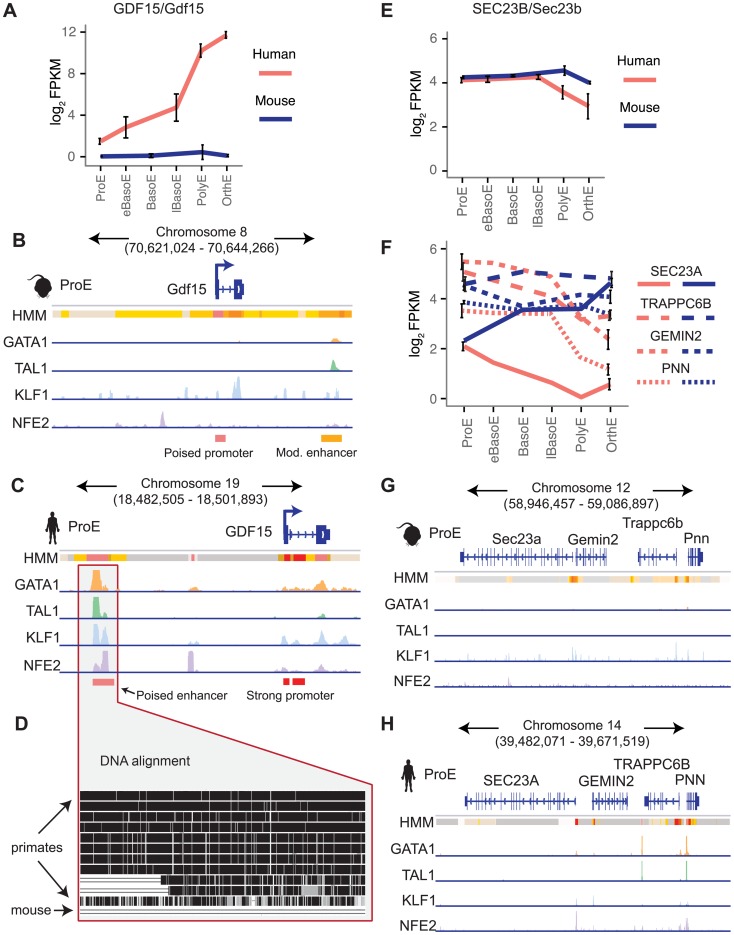
Species-specific expression of *GDF15* is driven by a human-specific element and the region around *SEC23A* in humans, but not mouse, is repressed. A) Gene expression of *GDF15* in human ProEs and *Gdf15* in mouse ProEs during terminal erythroid differentiation. Error bars represent the mean +/- the standard deviation. Mouse *Gdf15* is expressed at very low levels, while human *GDF15* is expressed at increasingly high levels during differentiation. B) Human GDF15 in ProEs has a strong promoter and is proximal to a poised enhancer (based on HMM) which is co-occupied by high levels of GATA1, TAL1, KLF1, and NFE2. C) In juxtaposition to human *GDF15*, mouse *Gdf15* has a poised promoter and is proximal to a moderately active promoter that contains GATA1 and TAL1 occupancy, but not KLF1 or NFE2. D) Multiple alignment of 46 vertebrates shows that the underlying genomic sequence of the poised enhancer element near *GDF15* is conserved across most primates but absent from the mouse genome. E) *SEC23B/Sec23b* are similarly expressed in both species. F) The SEC23B/Sec23b paralog, SEC23A/Sec23a, is differentially expressed across species. Three nearby homologous genes (TRAPPC6B, GEMIN2, PNN) show similar species-specific gene expression patterns. G) The genomic region surrounding human *SEC23A* is generally in a state of heterochromatin or polycomb repression. H) Relative to the orthologous human region, the region surrounding *Sec23a* is permissive of transcription. *Abbreviations used: ProE, pro-erythroblast; FPKM, fragments per kilobase per million.*

We observed no clear differences in TF occupancy or histone state at *SEC23B*, and this gene is similarly expressed between species (Fig. 7E, S15A–B Fig.). Thus, we focused on *SEC23A*. While we observed some small differences in TF occupancy between species, the most striking difference is that the local region surrounding human *SEC23A* is in a general state of heterochromatin (state 13) or polycomb repression (state 14), whereas the region around mouse *Sec23a* is comparatively open for transcription (Fig. 7G–H). Expanding out to a small region around *SEC23A*, three homologous genes are present in both species and exhibit similar species-specific chromatin states as well as similar gene expression pattern corresponding to their matching *SEC23A/Sec23a* gene (Fig. 7F–H). This suggests that transcription in the local region around *SEC23A* is repressed in humans whereas the homologous region around mouse *Sec23a* is significantly more transcriptionally permissive. This finding not only provides evidence for why knockout of *Sec23b* in mouse does not recapitulate the human disease phenotype, but also highlights a principle of epigenomic divergence: in concordance with our simplified promoter model (Fig. 5A–C), the local genomic region has transitioned during evolution from a low/active state in mouse to a repressed state in humans, and transcription has been blunted as a result. Alternatively, the reverse possibility may have occured: for an unknown reason transcription has decreased in this region, driving the chromatin changes that are observed.

Finally, we investigated a locus of interest where a gain of TF occupancy in a non-homologous genomic region is associated with species-specific gene expression (observed globally in Fig. 5F–G). Growth differentiation factor 15 (*GDF15*) is one of the most highly expressed genes in human differentiating erythroblasts but is absent in mouse erythroblasts (Fig. 7A) [11], [48]. GDF15 has been suggested to play an important role in the regulation of iron homeostasis as a result of changes in the extent and effectiveness of erythropoiesis [49]. Patients with β-thalassemia and other diseases characterized by ineffective erythropoiesis show increased levels of GDF15 expression [50]. By analyzing epigenomic patterns at this locus, we identified a species-specific difference in chromatin structure at the *GDF15* locus: human *GDF15* in ProEs has a strong promoter, whereas mouse *Gdf15* in ProEs has a poised promoter (Fig. 7B–C). Most importantly, while we identified some TF occupancy near mouse *Gdf15*, we identified a novel, putative CRM occupied by GATA1, TAL1, KLF1, and NFE2 upstream of human *GDF15* that is absent from the larger region that encompasses mouse *Gdf15* (Fig. 7B–D). Comparing the underlying genomic sequence of this putative enhancer across species, we found that this region is highly conserved in primates, but not in mice (Fig. 7D), suggesting that human *GDF15* expression may be driven by this element that is absent from mouse.

## Discussion

While numerous studies have been performed to understand how epigenomic modifications play a role in mediating cellular differentiation, only a limited number of studies have examined how these modifications have been altered during the course of evolution [29], [32], [34], [51]–[53]. Here, we have used erythropoiesis as a model of cellular differentiation to study how epigenomic modifications can underlie evolutionary changes in gene expression. We performed a systematic comparative analysis of occupancy for six histone modifications and four master TFs in both human and mouse primary ProEs as well as in erythroid cell lines, integrating our results with high quality gene expression data. Models based upon promoter marks were highly predictive of gene expression and were nearly identical for both human and mouse ProEs. While we observed that chromatin modifications, at least in promoter regions, were generally conserved across species, subtle differences in H3K9ac, H3K4me3, H3K27me3, and H3K9ac were associated with differential gene expression between species. This finding partially accounts for the previously reported divergence in gene expression during terminal erythroid differentiation across species [10], [11].

We found that only ∼25% of GATA1, TAL1, and NFE2 occupancy sites present in mouse ProEs were conserved in human ProEs; however, the loss of these sites is often offset by the acquisition of nearby compensatory TF occupancy sites. To some degree, compensatory sites appear to buffer transcriptional changes that occur from the original loss. This finding is consistent with the reported conservation and compensatory action of master regulators in other cell types that are found between closely related species [33], [34], [53]. In juxtaposition to other master regulators, KLF1 occupancy was highly conserved between human and mouse, approaching the conservation rates of TFs in closely related species of insects [52], [53]. Acting in combinatorial fashion with GATA1 and TAL1, we show that CRMs co-occupied by these three TFs are under strong evolutionary constraint and localize to genes that play a key role in defining erythroid cell state.

The critical role of these TFs in defining erythroid cell state is highlighted by human genetic studies that have identified causal mutations for various forms of anemia in *GATA1* and *KLF1* [54]–[58]. We suggest that disruption of these modules in either human or mouse progenitor cells would severely compromise terminal erythroid differentiation, and thus these regions may harbor non-coding polymorphisms in humans that underlie human erythroid disorders. In particular, polymorphisms that disrupt a GATA1, TAL1, or KLF1 binding motif in conserved or strong species-specific occupancy sites would be leading candidates for causal mutations in these disorders. For example, we identified that GATA1 and TAL1 co-occupy the first intron of *UROS* in both human and mouse. While coding mutations in *UROS* have been identified in over 50% of patients with congenital erythropoietic porphyria, rare mutations that disrupt a constrained GATA1 binding site in the first intron of *UROS* have been found in patients lacking a putative coding mutation [59]. Indeed, in an era when whole genomes of patients with various diseases can be readily sequenced, identification of causal mutations is frequently a difficult problem [60], [61], and results from this study could help prioritize variants identified by such approaches. Furthermore, in Mendelian diseases where a pathogenic coding variant is not immediately identifiable, targeted sequencing of conserved TF occupancy sites near causal genes could prove useful as an inexpensive and likely high-yield approach in comparison to whole genome sequencing.

In contrast to mouse ProEs, we found that TF occupancy in K562 cells is strongly conserved with human ProEs. We suggest that for certain erythroid disorders, K562 cells may more faithfully recapitulate features of the disease than primary mouse cells, particularly in cases where epigenetic or transcriptional regulation may be disrupted. The framework we have created provides an opportunity to prospectively ascertain the extent of conservation between mice and humans for various aspects of the transcriptional landscape underlying erythroid differentiation.

In this study, we confirmed and uncovered multiple principles of epigenomic conservation. We found that conserved TF occupancy between species is strongly associated with active regulatory regions and strong transcriptional activity during terminal erythroid differentiation. Similarly, the strongest human-specific TF occupancy sites were also associated with regions of active regulation and strong transcription. When extrapolating on information gained from TF occupancy in mouse ProEs, it is important to consider not only that ∼75% of regions are not conserved in humans, but also that regions of lost TF occupancy exhibit reduced regulatory modifications as well as, on average, reduced transcription of genes across all stages of terminal erythroid differentiation. As a result, we emphasize the importance of using such a comparative framework when examining whether findings from mouse models of erythropoiesis may have relevance to human blood production.

We have used our framework to interrogate specific regulatory regions as well as genes important in erythropoiesis to illustrate and provide vignettes for the principles that we identified on a more global scale. In particular, the results we present provide evidence that human *GDF15* is actively transcribed and contains unique CRMs not found near mouse *Gdf15*, consistent with its reduced expression in mouse erythroid cells. This finding is important when interpreting the role of Gdf15 in mouse models, and further investigation on the epigenetic regulation of *GDF15* may help explain variation in iron and erythroid homeostasis between mice and humans. In other cases the epigenomic landscape is more conserved, such as at the β-globin or *BCL11A* gene loci, although subtle variation may explain the species-divergent gene expression patterns that are observed.

Some limitations should be considered when interpreting the results of our study. First, while we included over 50 ChIP-seq datasets in our analysis, there are other histone modifications (e.g. H3K27ac), TFs (e.g. ZFPM1 and SPI1/PU.1) non-coding RNAs, and methylation patterns that may be important for understanding species-specific differences in erythropoiesis. Furthermore, we cannot exclusively rule out the possibility that certain peaks are “hyper-ChIPable” due to a lack of IgG control datasets, although recent work provides convincing evidence that this consideration, while critical in yeast, is far less of a concern in complex metazoans [62]–[64]. Finally, although the ProE stage is ideal to investigate epigenetic changes that occur to mediate alterations in the transcriptional landscape [19], [20], [26], [27], we did not investigate temporal changes in epigenomic marks during earlier or more terminal stages of differentiation where species-specific differences may be more pronounced [34].

We have made all of our results publically available as filetypes that are quickly loaded into standard genome browsers (IGV and UCSC Genome Browser). These data could guide investigators in choosing appropriate model systems for studying blood diseases or other aspects of erythropoiesis as well as aid in the interpretation of their results. Overall, our comparative epigenomics approach has successfully explained a significant portion of the transcriptional divergence observed during erythroid differentiation in mice and humans.

## Materials and Methods

### Reference genomes and annotations

Hg19 and mm10 were used throughout the entire analysis as reference genomes for all human and mouse cell types, respectively. Orthologous genes were defined using Ensembl mouse to human ortholog matching and were downloaded from BioMart; genes which matched one: many were excluded from all analyses, resulting in 15506 one: one orthologous genes used for analysis. A smaller subset of orthologous genes (6596) with well-defined canonical transcripts from RefSeq was used for all quantitative promoter analyses. To compare mm10 to hg19, the UCSC liftOver tool was used to lift coordinates over from one genome to another with one: one matching and 10% sequence conservation required. PAVIS was also used to annotate genomic regions such as TF-occupancy peaks based upon proximity to known genes [65].

### ChIP-seq data and statistical analyses

ChIP-seq datasets were either downloaded from NCBI GEO or from the ENCODE project's homepage (S1 Fig.). SRA files were transformed to FASTQ using FASTQ -dump from the NCBI SRA toolkit (https://www.ncbi.nlm.nih.gov/books/NBK158900/). Raw reads were aligned to the hg19 and mm10 genomes using Bowtie v0.12.9 with options “-v 2 -m 3 —strata –best” [66]. The BEDTools suite was used for multiple operations, comparisons, and intersections of all resultant BED files [67]. Reads were extended to a fragment length of 200bps, normalized to million-mapped-reads, and control input (in million-mapped-reads) were subtracted. In all quantitative analyses, reads were log_2_ scaled and read into R 3.0. NGSplot was used to plot normalized average intensity curves across 15506 orthologous genes (-2000 from TSS to +2000 after TES) for all ChIP-seq datasets [68].

TF-occupancy peaks were initially called using MACS 1.4 to estimate fragment size [69]. When replicates were present (e.g. GATA1 and TAL1, S1 Fig.), MM-ChIP was used to combine and robustly call peaks from datasets across multiple laboratories and technical conditions to create sets of high-quality peaks [70]. The top n-percentile of each set of peaks was defined based upon the total number of mapped reads present in the peak region. When regions/peaks were lifted over from one species to another, the denominator used was always the number of regions which mapped to the new genome successfully from the original genome, while the numerator was the number of mapped regions that overlapped with the target region in the new genome. If peaks from a single TF were lifted across genomes, only a narrow region (+/− 50 bps around summit) was mapped to reduce the probability of incorrect mappings due to non-functional decrease in sequence similarity at the far edges of peak regions called by MACS. DNA motif enrichment was performed using MEME-ChIP in the MEME Suite with standard options [71]. E-values are reported as corrected p-values in all figures. Enrichment of combinatorial TF occupancy was assessed using 100,000 permutations across the genome with the Genomic Association Tester [72]. Chromatin states were estimated for 200 bp bins spanning both genomes using a Hidden Markov Model (ChromHMM) [38], [73]. We settled on a 15 state model learned on all three cell types together, although we examined models ranging from 12 to 20 states (Fig. 4A and S7 Fig.). Biological relevance for each state was assigned based upon frequency of chromatin marks and functional enrichments similar to previous studies [73]. For example, the “Active Transcription” state is marked exclusively by H3K36me3 and is enriched primarily for exonic regions whereas the “Strong Promoter” state is marked by H3K4me2 and H3K4me3 but not H3K4me1 and is enriched for TSSs. The final model was highly conserved between models derived exclusively from mouse ProEs, human ProEs, and K562 cells (Fig. 4A, S8 Fig., S9 Fig.). Enrichment of chromatin states across regions of TF occupancy was compared using the “OverlapEnrichment” command in ChromHMM.

We performed multiple analyses to address the possibility that ChIP protocol differences may underlie the TF occupancy differences observed in analyses such as promoter differences and peak calling. First, we note that we were able to recover large sets of peaks (>5000 peaks) for each TF in each species, suggesting that immunoprecipitation was nominally successful. Indeed, western blots in human and mouse cell types suggest that these antibodies are specific for both human and mouse TFs (http://genome.cse.ucsc.edu/cgi-bin/hgEncodeVocab?ra=encode%2Fcv.ra&term=%22GATA1_(SC-266)%22 and 2TAL1_(SC-12984)%22). Importantly, these peaks were all significantly enriched for the TF canonical motif (Fig. 2D). This result is not surprising, given that human and mouse GATA1 and TAL1 share >86% similarity in amino acids based upon the Ensembl database. Furthermore, when we investigated occupancy site conservation between species by performing a sensitivity analysis on only the strongest 25% of peaks, the conservation rate of the least conserved TF (TAL1) showed a similar increase to that of the most conserved TF (KLF1), suggesting limited bias between species (S5 Fig.). Based upon this evidence, we believe that the most likely reason for the observed difference in absolute occupancy between species is that certain aspects of the protocols used vary between species and introduce bias such that weaker peaks in mouse ProEs may not be as readily observed in these cases. Alternatively, the difference in the number of occupancy sites could be a true biological observation. Regardless of the case, our estimates for conservation of TF occupancy scale with the absolute number of peaks called in mouse. In other analyses, we normalized between human and mouse to account for these differences.

### RNA-seq data and statistical analyses

Single-end RNA-seq datasets of primary erythroblasts were downloaded from NCBI GEO (GSE53983) [11]. K562 and G1E RNA-seq data was publically available from ENCODE and was also downloaded NCBI GEO (GSE40522) and from the ENCODE website (http://genome.ucsc.edu/ENCODE/). Five human time points (ProE, early BasoE, late BasoE, PolyE, OrthoE) and four mouse time points (ProE, BasoE, PolyE, OrthoE) with three replicates each were used in this analysis. Similar to ChIP-seq data processing, SRA files were transformed to FASTQ using FASTQ-dump. RNA-seq data was processed with the Tuxedo Tools Suite using the same options as recent protocols, except CuffQuant and CuffNorm were used to derive normalized (FPKM; fragments per kilobase of transcript per million mapped reads) and raw count data for each transcript [74]. In particular, raw reads were aligned to the genome using TopHat v2.0.10. Cufflinks v2.1.0 was used to assemble transcripts, CuffMerge was used to merge all annotations (separated by species), and CuffQuant and CuffNorm were used to output data at the gene, TSS, and promoter level that was then imported to R. All Tuxedo Suite tools were run using standard options as indicated [74].

For predictive analyses of ProE gene expression, we first quantile normalized our epigenomic profiles to account for any species-specific biases in intensity and integrated this dataset with stage-matched RNA-seq data. In order to derive predictive models of transcription for each species without over fitting parameters, we performed standard linear regression with L_1_-penalization (i.e. lasso regression, [75]). In this model, the β value of each predictor is shrunk towards zero until an optimum solution is reached; variables that add little predictive value are excluded (β = 0). Subsequently, 100-fold cross validation is performed for different lambda “penalization” values and a lambda one standard error lower than the best model was chosen to prevent over-fitting. In R, glmnet was used to perform L_1_-penalized linear regression [75].

### Analysis of specific loci

We used the Integrative Genomics Viewer (IGV) to view epigenomic mark intensity files [76]. Bed files of aligned reads were extended to a fragment size of 200 bps and intensity files (bigwig) were created using UCSC Genome Browser tools. Enrichments were shown on a log scale unless otherwise noted and a cut-off of 20 bps was used as a lower bound in all representations unless otherwise noted. Phastcons nucleotide substitution rate score (0 to 1) and the primate and mouse (mm10) to human (hg19) alignments from the multiz 46-vertebrate multi-alignment were also used as available for import from the IGV servers [77], [78].

### Data access

We have made aligned and processed ChIP-seq data for all six histone modifications, four transcription factors, and derived chromatin states for all cell-types available on GEO at GSE59801. Gene expression data processed in our pipeline is also available as aligned reads and as FPKM and counts for each gene. Furthermore, robustly defined transcription factor occupancy peaks and information regarding conservation, gain, or loss across species as discussed in the analyses is also made available in the same location.

## Supporting Information

S1 Fig **Conservation of histone modifications in orthologous promoters continued from** **Fig. 1** **.** A)–B) Corresponding plots for A) H3K4me1 and B) H3K36me3. *Left*: Average curves of normalized log_2_ fold changes across 15506 orthologous genes for each histone mark. The size of each gene is normalized in order to represent the average shape of histone mark intensity. *Middle*: Heatmaps are clustered by the similarity of the pearson r for histone mark intensities between all cell-types. hProEs are CD71+ mProEs are Ter119+, K562 cells are a human erythroid cell line, and G1E/G1E-ER cells are a mouse erythroid cell line. Replicates are included as independent observations. *Right*: For each category shown (e.g. Mouse ProEs, K562, and G1E/G1E-ER), the average pearson correlation between each replicate of that type and each replicate of human ProEs is presented as boxplots. *Abbreviations used*: hProE, human pro-erythroblast; mProE, mouse pro-erythroblast.
(PDF)

S2 Fig **H3K4me3 and H3K27me3 associate with expressed and repressed genes, respectively.** A) Cumulative density function of H3K4me3 and H3K27me3 intensity across promoter regions. H3K4me3 is enriched at about ∼50% of orthologous genes while H3K27me3 is enriched at ∼20% of orthologous genes. These two marks are found together at about 2% of orthologous genes. B) Genes with H3K4me3 (>1 input normalized rpm) are highly expressed while genes with H3K27me3 (0.5 input normalized rpm) are repressed. Genes with both marks show a slight increase in expression compared to H3K27me3 alone. *Abbreviations used*: rpm, reads per million.
(PDF)

S3 Fig **Patterns of transcription factor occupancy across promoters.** A)–D) *Left*: Average curves of normalized log_2_ fold changes across 15506 orthologous genes for each TF occupancy. The size of each gene is normalized in order to represent the average shape of histone mark intensity. *Right*: Example heatmaps are ordered by intensity of aligned reads per million for each species. *Abbreviations used*: hProE, human pro-erythroblast; mProE, mouse pro-erythroblast.
(PDF)

S4 Fig **Divergence of transcription factor intensity in orthologous promoters.** A)–D) *Left*: Heatmaps are clustered by the similarity of the pearson r for TF occupancy intensities between all cell-types. hProEs are CD71+ cells, peripheral blood derived erythroblasts, or CD36+ mProEs are Ter119+ or FDCPmix cells, K562 cells are a human erythroid cell line, and G1E/G1E-ER cells are a mouse erythroid cell line. Replicates are included as independent observations. *Right*: For each category shown (e.g. Mouse ProEs, K562, and G1E/G1E-ER), the average spearman correlation between each replicate of that type and each replicate of human ProEs is presented as boxplots. *Abbreviations used*: hProE, human pro-erythroblast; mProE, mouse pro-erythroblast.
(PDF)

S5 Fig **Alternate estimates of transcription factor occupancy conservation.** A) For each TF (GATA1, TAL1, KLF1, and NFE2), the estimated conservation rate (0–100%) of TF peaks between human and mouse ProEs are displayed. The lower bound represents the percentage of human peaks in orthologous genomic regions that are also present in mouse (left box). The middle estimate is the percentage of mouse peaks in orthologous genomic regions also present in human (middle box). The upper estimate is the same as the middle estimate, except that we first restricted to only the top 25% of peaks in mouse (left box). Conserved peaks are peaks that overlap in orthologous genomic regions, and compensatory peaks are peaks that are within +/- 5 kbs of the original peak. We expected to observe at least a small increase in the total percentage of conserved peaks for each TF, since stronger peak signals may be more indicative of function. Indeed, we observed a moderate increase (average increase of 18%) in percentage of conservation across each TF but also a significant drop (average 2.5-fold decrease) in the total number of conserved peaks. B) Similar to A), except that that the estimated number of conserved peaks are displayed instead of the percentage. Please note that this is the total number of overlapping mapped peaks, and not the total number of mapped peaks. *Abbreviations used*: ProE, pro-erythroblast.
(PDF)

S6 Fig **Graphical depiction of TF occupancy lift over and comparison.** A) Briefly, for each TF occupancy peak (restricted to GATA1, TAL1, KLF1), we determined if it is co-occupied (+/- 1000 bps) by any of the other TFs resulting in 7 different combinatorial groups (e.g. GATA1; TAL1; KLF1; GATA1 and TAL1; GATA1 and KLF1; TAL1 and KLF1; and GATA1, TAL1, and KLF1). A narrow region (+/- 50bp) around the summit of each of these TF occupancy peaks is mapped from mouse ProEs (mm10) to hg19 and its corresponding group (including no overlap) is computed similarly in human ProEs. ‘n’ is the total number of mapped peaks for each group and ‘x_i_’ is the number of mapped peaks that fall into the ‘i’th group. *Abbreviations used*: ProE, pro-erythroblast.
(PDF)

S7 Fig **Transition matrix of chromatin state HMM.** A) Transition matrix of the consensus HMM for each state. Blue represents increased probability of transition between states. *Abbreviations used*: HMM, hidden markov model.
(PDF)

S8 Fig **Fold enrichments of genomic regions for consensus HMM.** A), C), E) Fold enrichments for hProEs, K562 cells, and mProEs on the consensus model derived across all cell types. B), D), F) Fold enrichment in 200bp bins around the TSS for each cell state. Blue represents increased fold enrichment versus expected. *Abbreviations used*: hProE, human pro-erythroblast; mProE, mouse pro-erythroblast; HMM, hidden markov model; TSS, transcription start site.
(PDF)

S9 Fig **Individual cell-type chromatin state HMMs.** A)–C) From left to right: emission probability matrix, fold enrichment matrix, fold enrichment around the TSS, and transition probability matrix for HMMs derived solely for each cell type (hProE, A), K562 cells, B), and mProE, C)). Darker blue represents increased probability (for emission probability matrix and transition probability matrix) or increased fold enrichment versus expected (for enrichment in genomic regions and around the TSS). *Abbreviations used*: hProE, human pro-erythroblast; mProE, mouse pro-erythroblast; HMM, hidden markov model; TSS, transcription start site.
(PDF)

S10 Fig **Chromatin state fold enrichment versus expected in K562 cells.** Identical regions as investigated in Fig. 4B–F. Specifically, the regions are A) TF occupancy sites conserved between human and mouse, B) compensatory TF occupancy sites that are human-specific and proximal (+/- 5 kb) to a lost TF occupancy site during evolution, C) human-specific occupancy sites that are gained during evolution, D) top 10% of human occupancy sites based upon mapped reads, and E) mouse-specific occupancy sites that are lost during evolution. Overall, promoter and enhancer regulatory regions are decreasingly enriched for conserved, compensatory and human-specific, and finally mouse-specific occupancy sites in K562 cells. F) Similar to A), C), and E) except enrichment is calculated for mouse ProEs. An opposite pattern of enrichment is observed. G) Comparison of K562 cell chromatin states to human ProE chromatin states. Generally, states are highly conserved, although enhancer states/polycomb repressed regions transition to each other between cell-types more than expected by chance. *Abbreviations used*: ProEs, pro-erythroblasts.
(PDF)

S11 Fig **Inter- and intra-species gene expression.** Spearman correlations for gene expression profiles between mouse and human primary ProEs, BasoEs (early and late, eBaso and lBaso), PolyEs, and OrthEs as well as erythroid cell lines, K562 and G1E, represented as a heatmap. The histogram in the legend represents the number of cells in the heatmap with a given correlation. Across all cell types, human and mouse gene expression profiles are more similar within- than between-species. *Abbreviations used*: ProEs, pro-erythroblasts; eBasoE, early basophilic erythroblasts; BasoE, basophilic erythroblasts; lBasoE, late basophilic erythroblasts; PolyE, polychromatic erythroblasts; OrthE, orthochromatic erythroblasts.
(PDF)

S12 Fig **Gene expression for transcription factor conservation classes in human terminal erythroid differentiation.** A)–D) Gene expression patterns during terminal erythroid differentiation based upon proximity to TF occupancy sites for varying TF occupancy conservation (defined in Fig. 4B–F and Fig. 5F–G) for each TF individually. *Abbreviations used*: ProEs, pro-erythroblasts; eBasoE, early basophilic erythroblasts; BasoE, basophilic erythroblasts; lBasoE, late basophilic erythroblasts; PolyE, polychromatic erythroblasts; OrthE, orthochromatic erythroblasts; FPKM, fragments of aligned reads per kilobase of transcript per million mapped reads.
(PDF)

S13 Fig **Cross species gene expression for transcription factor conservation classes.** A)–D) Cross species gene expression patterns during terminal erythroid differentiation based upon proximity to TF occupancy sites for varying TF occupancy conservation (defined in Fig. 4B–F) for each TF individually. Positive corresponds to human-specific expression and negative correspond to mouse-specific expression. *Abbreviations used*: ProEs, pro-erythroblasts; eBasoE, early basophilic erythroblasts; BasoE, basophilic erythroblasts; lBasoE, late basophilic erythroblasts; PolyE, polychromatic erythroblasts; OrthE, orthochromatic erythroblasts; FPKM, fragments of aligned reads per kilobase of transcript per million mapped reads.
(PDF)

S14 Fig **Applying the comparative epigenomics framework to** *BCL11A* **.** A) Chromatin states learned from the HMM and TF intensities are shown for *BCL11A* in human ProEs. B) Chromatin states learned from the HMM and TF intensities are shown for *Bcl11a* in mouse ProEs. C) Genomic conservation of a GATA1/TAL1 binding site in an erythroid-specific enhancer across species. D) Gene expression patterns during terminal erythroid differentiation are shown for *BCL11A* by species. Error bars represent the mean +/- the standard deviation. *Abbreviations used:* ProE, pro-erythroblast; HMM, hidden markov model.
(PDF)

S15 Fig **Comparative epigenomics of the** *SEC23B* **locus.** Chromatin states learned from the HMM and TF intensities are shown for A) *SEC23B* in human and B) *Sec23b* in mouse. Legend for the HMM is shown in Fig. 4A. Some differences are observed between TF occupation and chromatin state, but gene expression across the orthologs is similar as shown in Fig. 7E. *Abbreviations used*: ProE, pro-erythroblast; HMM, hidden markov model.
(PDF)

S1 Table **Raw ChIP-seq datasets.** ChIP-seq datasets for each cell-type analyzed in this manuscript. Raw data was available on either NCBI GEO or the ENCODE website. *Abbreviations used*: ChIP-seq, chromatin immunoprecipitation high-throughput sequencing.
(PDF)

S2 Table **ChIP-seq summary statistics.** Summary statistics for each ChIP-seq dataset used in the analysis. *Abbreviations used*: none.
(PDF)

S3 Table **Values for correlation matrices.** Spearman correlation matrices for H3K4me3, H3K27me3, and H3K4me1. Matrices shown examples of biological replicate correlations for each mark as well as low correlations between K562 and G1E cell types. *Abbreviations used*: ChIP-seq, chromatin immunoprecipitation followed by high-throughput sequencing.
(PDF)

S4 Table **GATA1, TAL1, and KLF1 conserved regions**. Coordinates in hg19 for human ProEs. Regions are centered around KLF1 peaks lifted over from mouse ProEs. *Abbreviations used*: ProE, pro-erythroblast.
(PDF)

S5 Table **Chromatin state functional enrichment for HMM.** Values corresponding to the heatmaps shown in Fig. 4A. *Abbreviations used*: none.
(PDF)

S6 Table **Predictive promoter models of transcription.** Both the species-specific and consensus models applied to promoter marks are applied to gene expression across terminal erythroid differentiation. *Abbreviations used*: none.
(PDF)

S1 Dataset **Mouse proerythroblast TAL1 motif discovery results.** Raw motif results. *Abbreviations used*: none.
(ZIP)

S2 Dataset **Mouse proerythroblast NFE2 motif discovery results.** Raw motif results. *Abbreviations used*: none.
(ZIP)

S3 Dataset **Mouse proerythroblast KLF1 motif discovery results.** Raw motif results. *Abbreviations used*: none.
(ZIP)

S4 Dataset **Mouse proerythroblast GATA1 motif discovery results.** Raw motif results. *Abbreviations used*: none.
(ZIP)

S5 Dataset **Human proerythroblast TAL1 motif discovery results.** Raw motif results. *Abbreviations used*: none.
(ZIP)

S6 Dataset **Human proerythroblast NFE2 motif discovery results.** Raw motif results. *Abbreviations used*: none.
(ZIP)

S7 Dataset **Human proerythroblast KLF1 motif discovery results.** Raw motif results. *Abbreviations used*: none.
(ZIP)

S8 Dataset **Human proerythroblast GATA1 motif discovery results.** Raw motif results. *Abbreviations used*: none.
(ZIP)
