# Supplementary figures and images for: Altered Chromatin Occupancy of Master Regulators Underlies Evolutionary Divergence in the Transcriptional Landscape of Erythroid Differentiation
Source: PLoS Genet. 2014 Dec 18;10(12):e1004890. doi: 10.1371/journal.pgen.1004890 (PMC4270484; doi:10.1371/journal.pgen.1004890)

**A**

**H3K4me1**

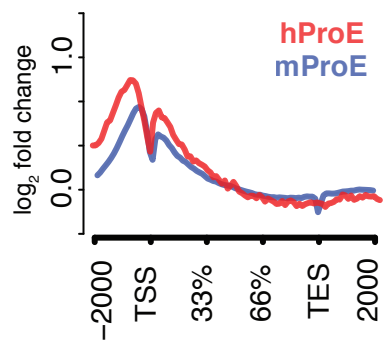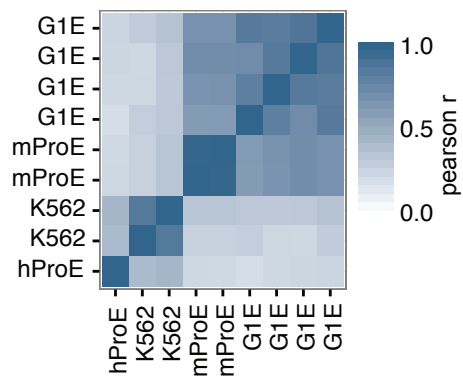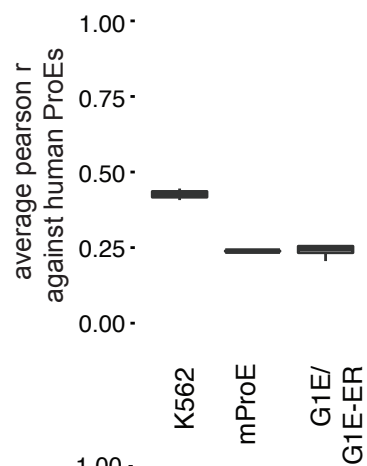

**B**

**H3K36me3**

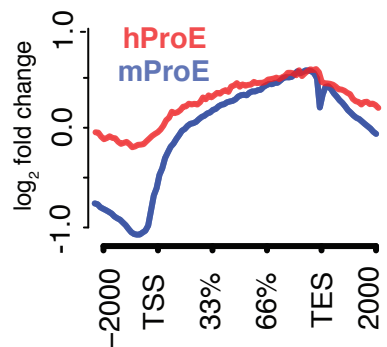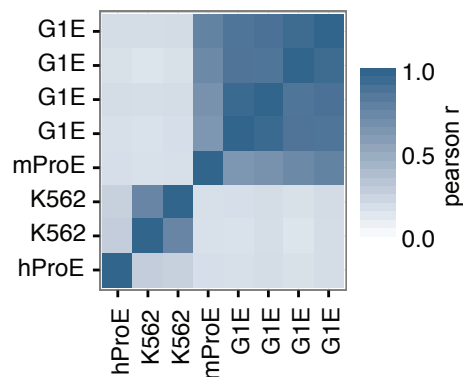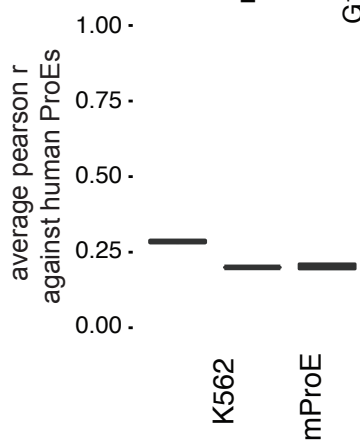

Supplement: S1 Fig — Conservation of histone modifications in orthologous promoters continued from Fig. 1 . A)–B) Corresponding plots for A) H3K4me1 and B) H3K36me3. Left: Average curves of normalized log2 fold changes across 15506 orthologous genes for each histone mark. The size of each gene is normalized in order to represent the average shape of histone mark intensity. Middle: Heatmaps are clustered by the similarity of the pearson r for histone mark intensities between all cell-types. hProEs are CD71+ mProEs are Ter119+, K562 cells are a human erythroid cell line, and G1E/G1E-ER cells are a mouse erythroid cell line. Replicates are included as independent observations. Right: For each category shown (e.g. Mouse ProEs, K562, and G1E/G1E-ER), the average pearson correlation between each replicate of that type and each replicate of human ProEs is presented as boxplots. Abbreviations used: hProE, human pro-erythroblast; mProE, mouse pro-erythroblast. (PDF) [file pgen.1004890.s001.pdf]

**A**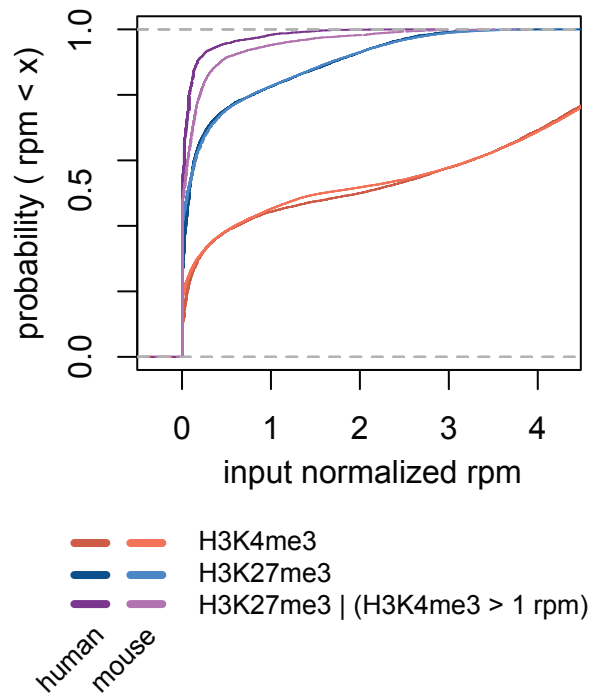**B**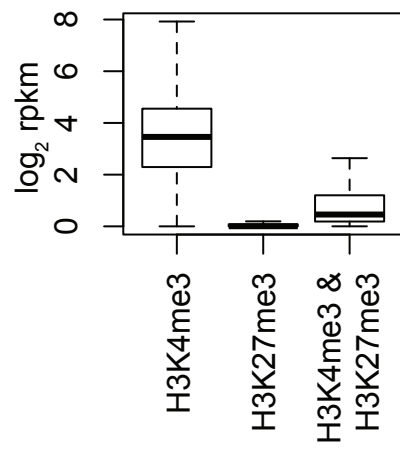

Supplement: S2 Fig — H3K4me3 and H3K27me3 associate with expressed and repressed genes, respectively. A) Cumulative density function of H3K4me3 and H3K27me3 intensity across promoter regions. H3K4me3 is enriched at about ∼50% of orthologous genes while H3K27me3 is enriched at ∼20% of orthologous genes. These two marks are found together at about 2% of orthologous genes. B) Genes with H3K4me3 (>1 input normalized rpm) are highly expressed while genes with H3K27me3 (0.5 input normalized rpm) are repressed. Genes with both marks show a slight increase in expression compared to H3K27me3 alone. Abbreviations used: rpm, reads per million. (PDF) [file pgen.1004890.s002.pdf]

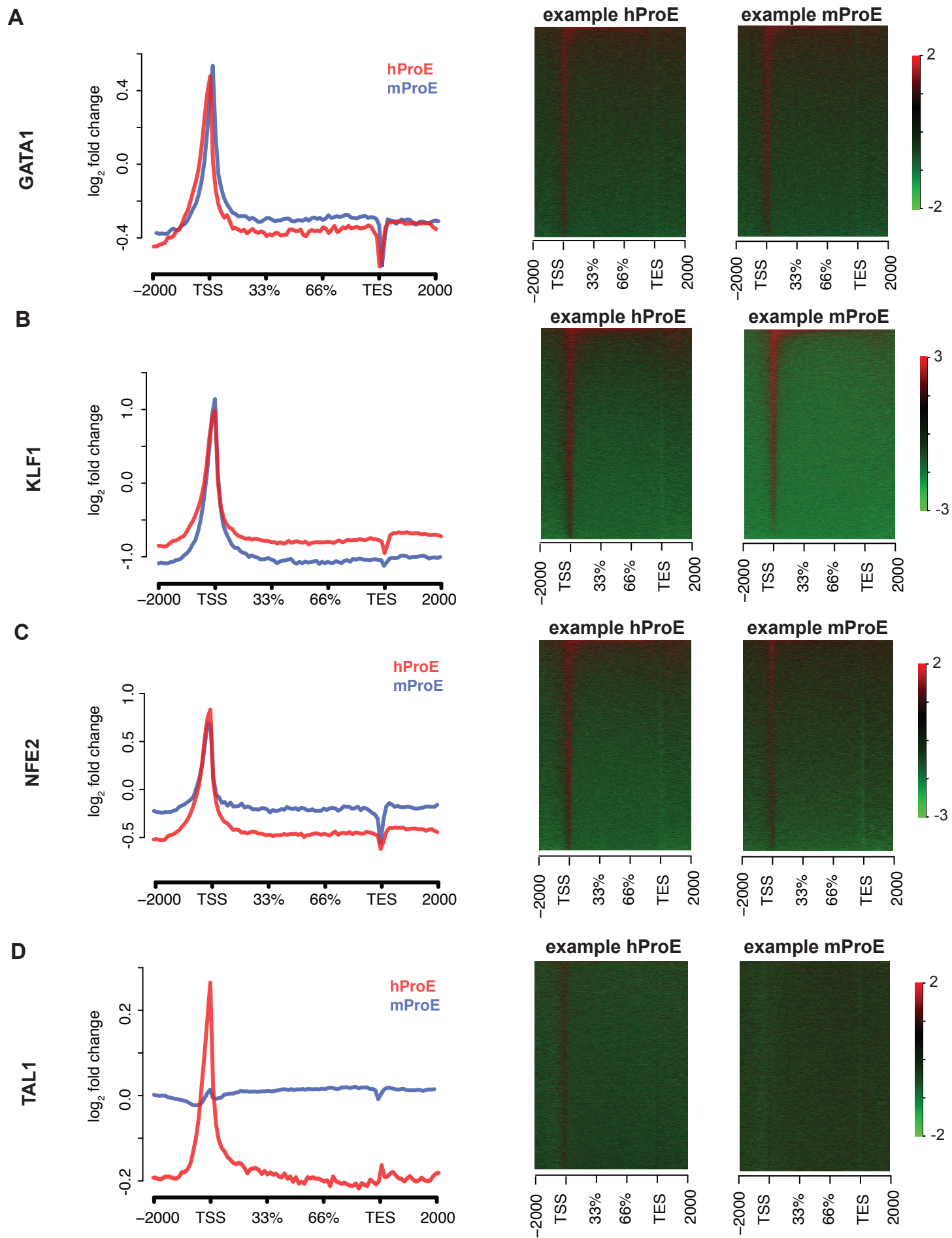

Supplement: S3 Fig — Patterns of transcription factor occupancy across promoters. A)–D) Left: Average curves of normalized log2 fold changes across 15506 orthologous genes for each TF occupancy. The size of each gene is normalized in order to represent the average shape of histone mark intensity. Right: Example heatmaps are ordered by intensity of aligned reads per million for each species. Abbreviations used: hProE, human pro-erythroblast; mProE, mouse pro-erythroblast. (PDF) [file pgen.1004890.s003.pdf]

A

GATA1

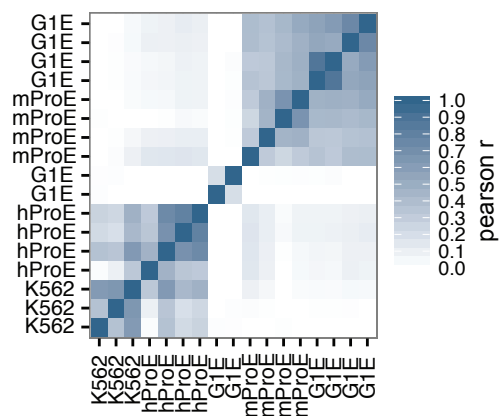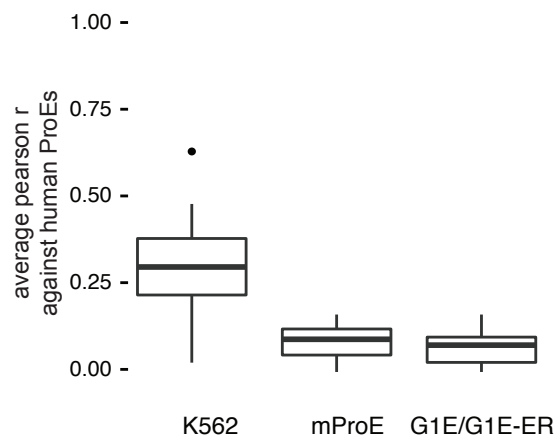

B

TAL1

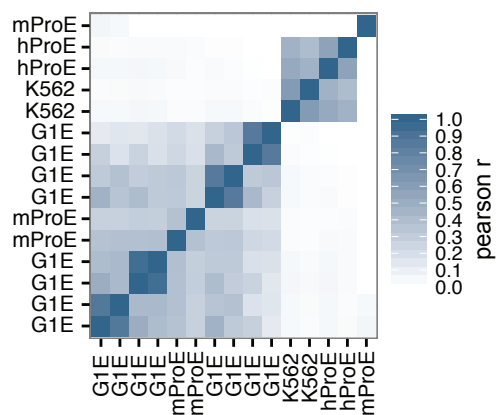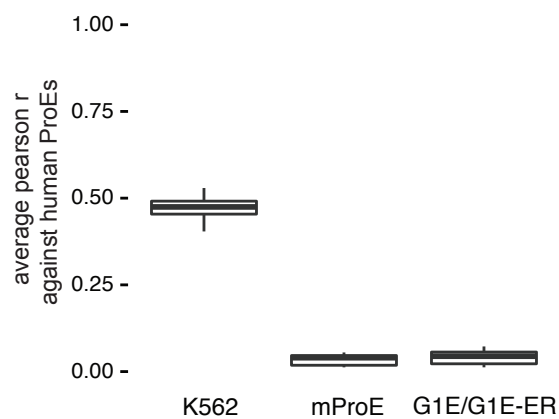

C

NFE2

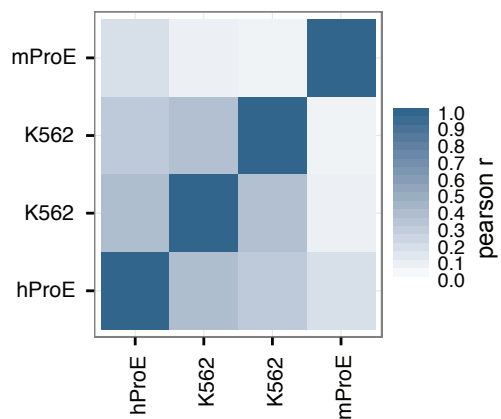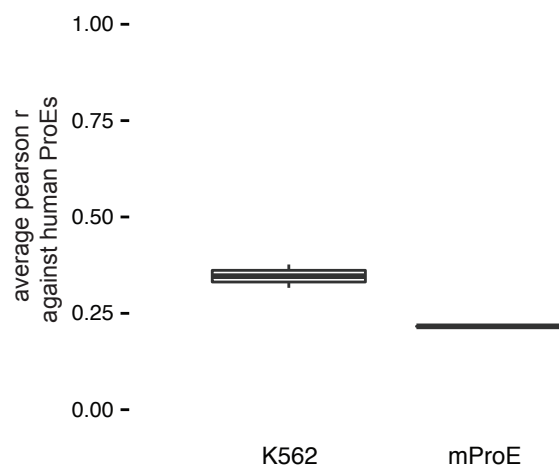

D

KLF1

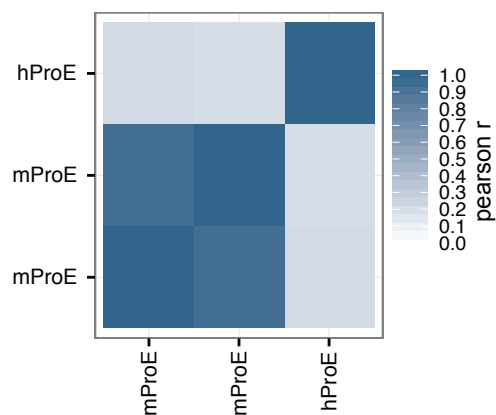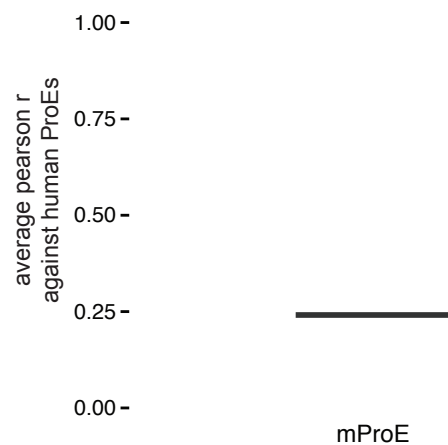

Supplement: S4 Fig — Divergence of transcription factor intensity in orthologous promoters. A)–D) Left: Heatmaps are clustered by the similarity of the pearson r for TF occupancy intensities between all cell-types. hProEs are CD71+ cells, peripheral blood derived erythroblasts, or CD36+ mProEs are Ter119+ or FDCPmix cells, K562 cells are a human erythroid cell line, and G1E/G1E-ER cells are a mouse erythroid cell line. Replicates are included as independent observations. Right: For each category shown (e.g. Mouse ProEs, K562, and G1E/G1E-ER), the average spearman correlation between each replicate of that type and each replicate of human ProEs is presented as boxplots. Abbreviations used: hProE, human pro-erythroblast; mProE, mouse pro-erythroblast. (PDF) [file pgen.1004890.s004.pdf]

**A**

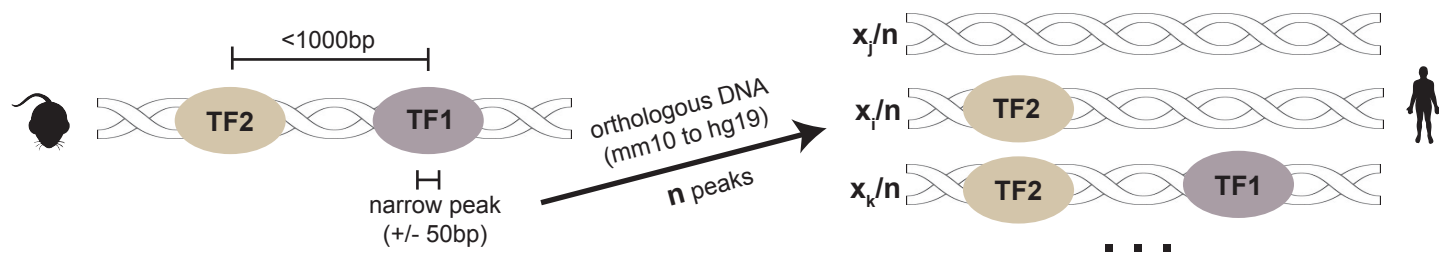

Supplement: S6 Fig — Graphical depiction of TF occupancy lift over and comparison. A) Briefly, for each TF occupancy peak (restricted to GATA1, TAL1, KLF1), we determined if it is co-occupied (+/- 1000 bps) by any of the other TFs resulting in 7 different combinatorial groups (e.g. GATA1; TAL1; KLF1; GATA1 and TAL1; GATA1 and KLF1; TAL1 and KLF1; and GATA1, TAL1, and KLF1). A narrow region (+/- 50bp) around the summit of each of these TF occupancy peaks is mapped from mouse ProEs (mm10) to hg19 and its corresponding group (including no overlap) is computed similarly in human ProEs. ‘n’ is the total number of mapped peaks for each group and ‘xi’ is the number of mapped peaks that fall into the ‘i’th group. Abbreviations used: ProE, pro-erythroblast. (PDF) [file pgen.1004890.s006.pdf]

# A

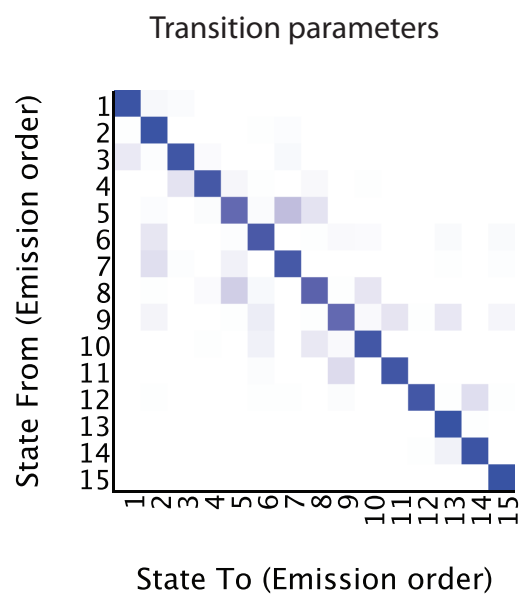

Supplement: S7 Fig — Transition matrix of chromatin state HMM. A) Transition matrix of the consensus HMM for each state. Blue represents increased probability of transition between states. Abbreviations used: HMM, hidden markov model. (PDF) [file pgen.1004890.s007.pdf]

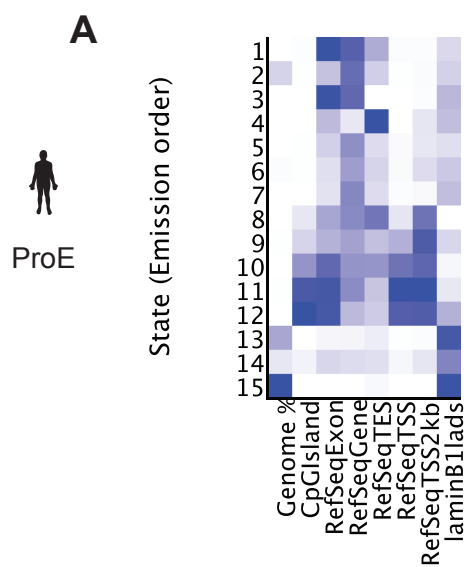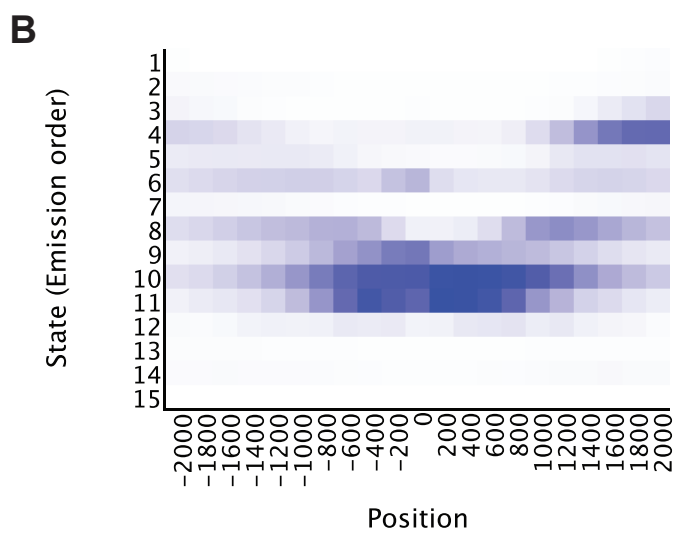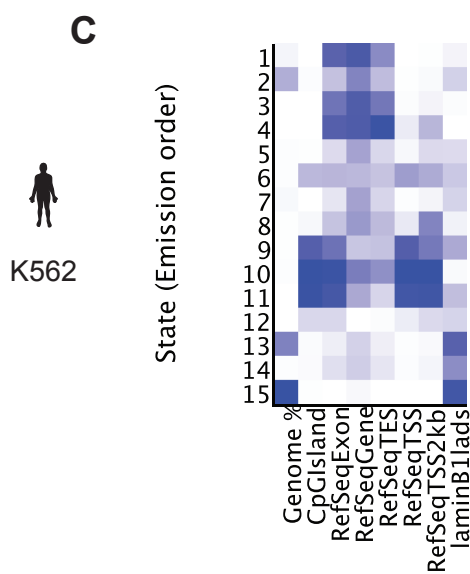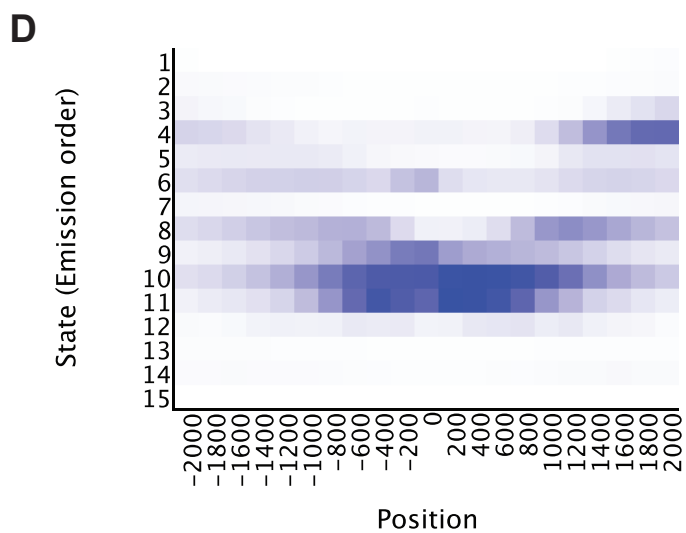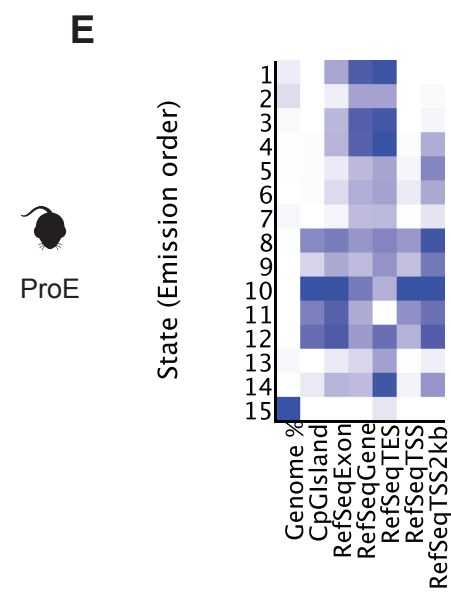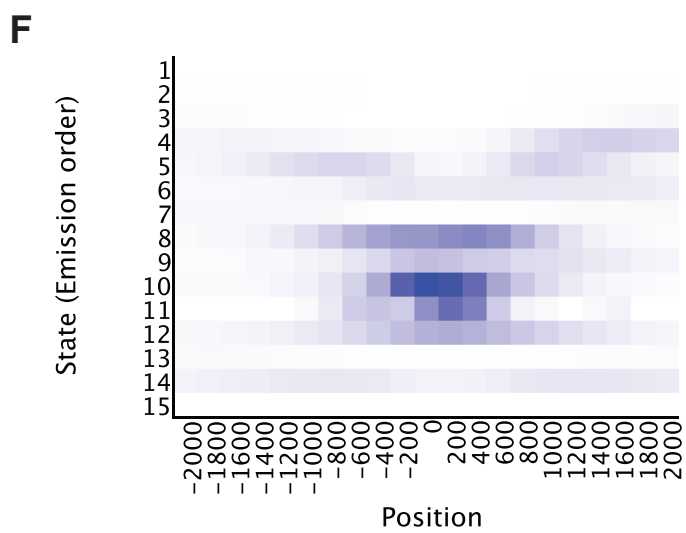

Supplement: S8 Fig — Fold enrichments of genomic regions for consensus HMM. A), C), E) Fold enrichments for hProEs, K562 cells, and mProEs on the consensus model derived across all cell types. B), D), F) Fold enrichment in 200bp bins around the TSS for each cell state. Blue represents increased fold enrichment versus expected. Abbreviations used: hProE, human pro-erythroblast; mProE, mouse pro-erythroblast; HMM, hidden markov model; TSS, transcription start site. (PDF) [file pgen.1004890.s008.pdf]

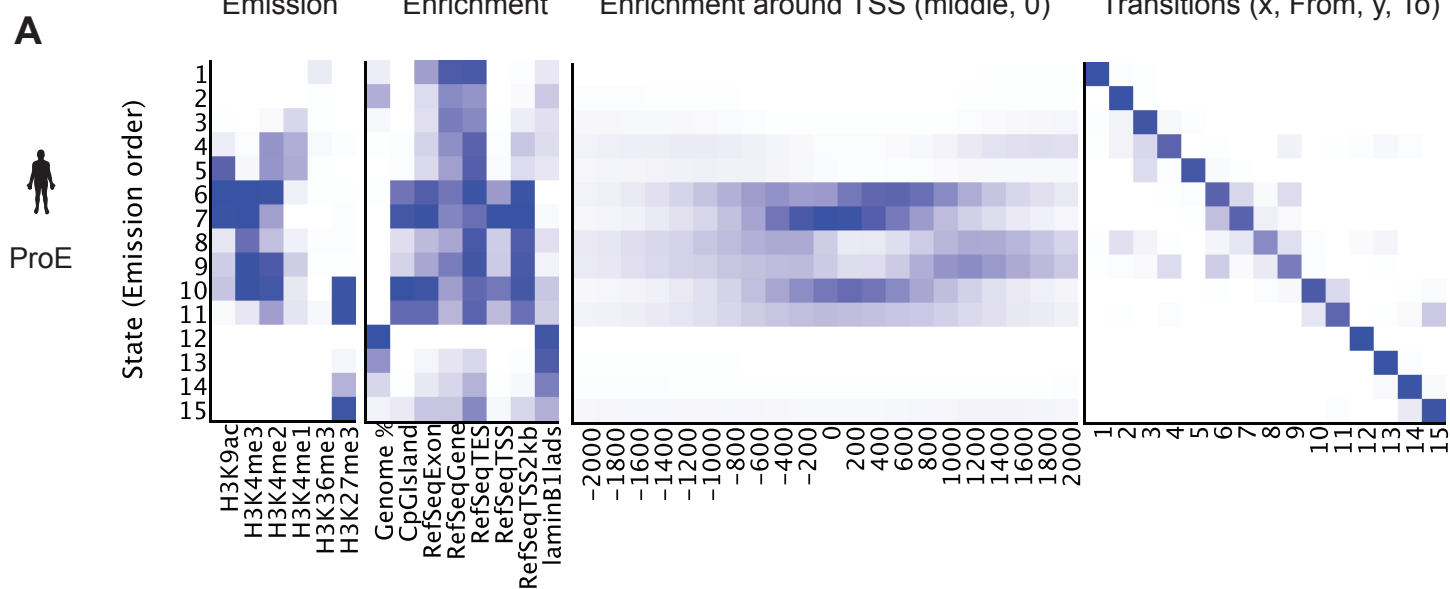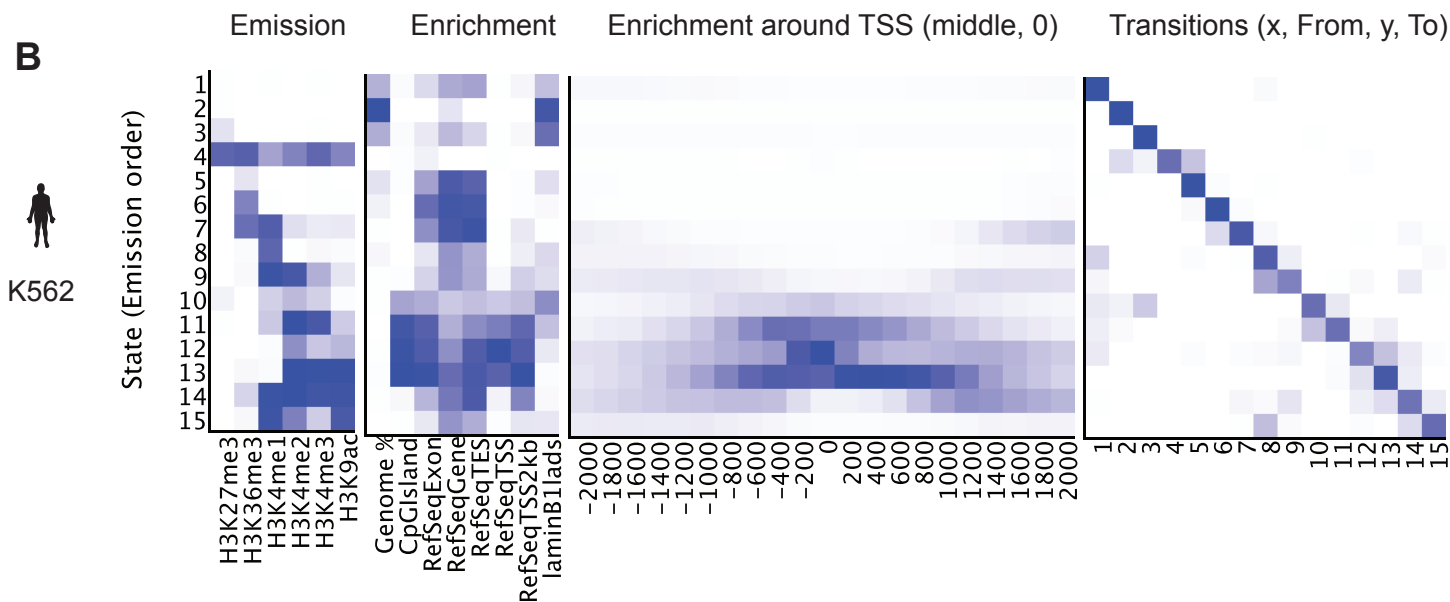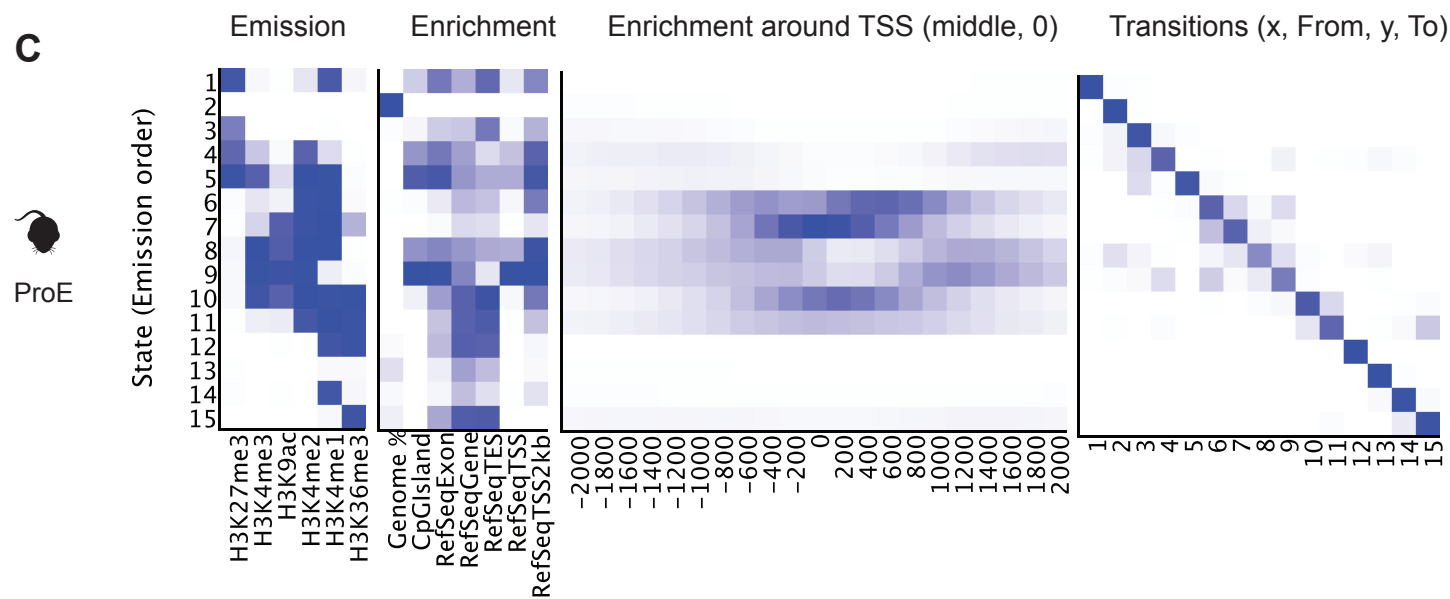

Supplement: S9 Fig — Individual cell-type chromatin state HMMs. A)–C) From left to right: emission probability matrix, fold enrichment matrix, fold enrichment around the TSS, and transition probability matrix for HMMs derived solely for each cell type (hProE, A), K562 cells, B), and mProE, C)). Darker blue represents increased probability (for emission probability matrix and transition probability matrix) or increased fold enrichment versus expected (for enrichment in genomic regions and around the TSS). Abbreviations used: hProE, human pro-erythroblast; mProE, mouse pro-erythroblast; HMM, hidden markov model; TSS, transcription start site. (PDF) [file pgen.1004890.s009.pdf]

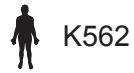

K562

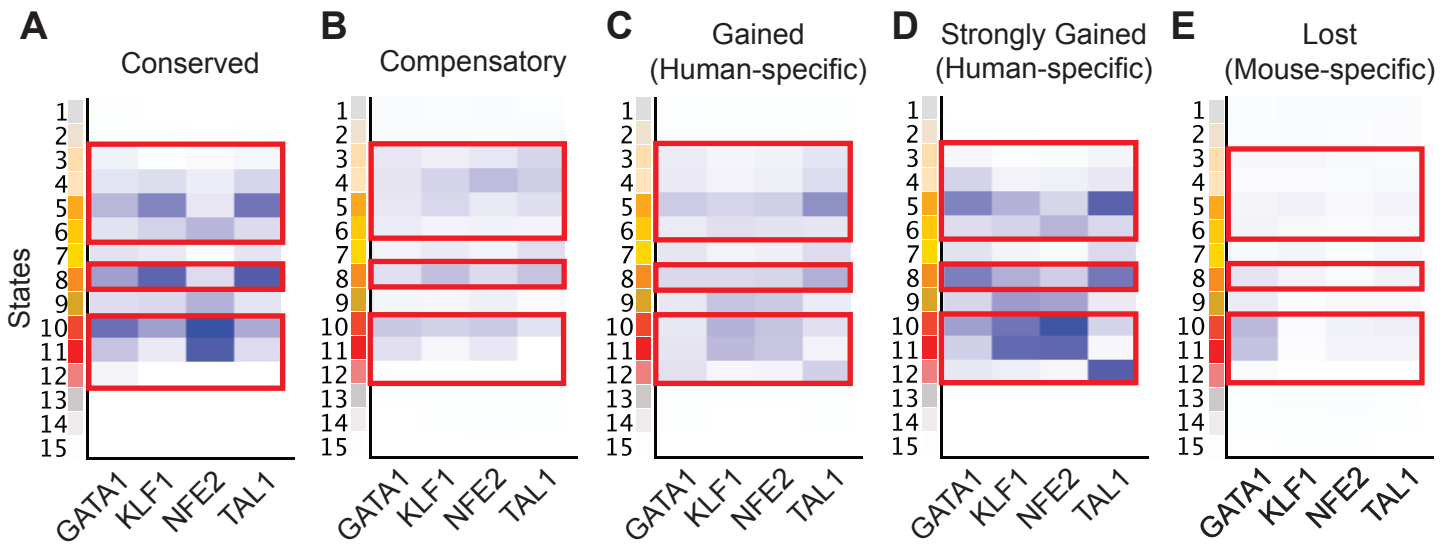

**F**

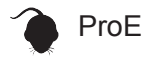

ProE

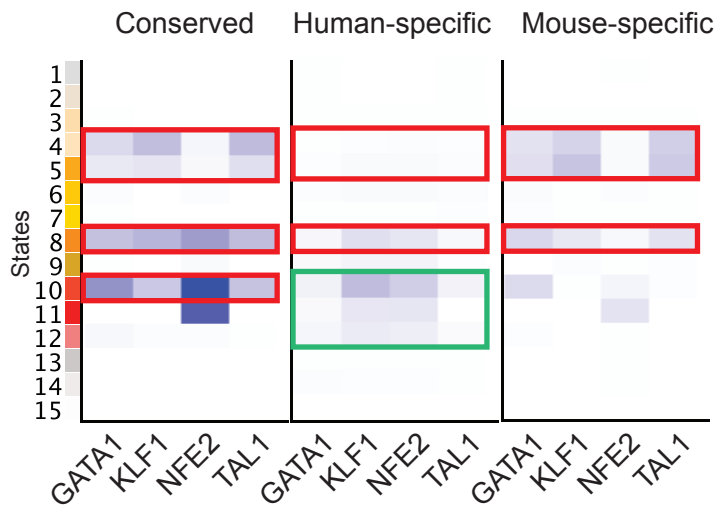

**G**

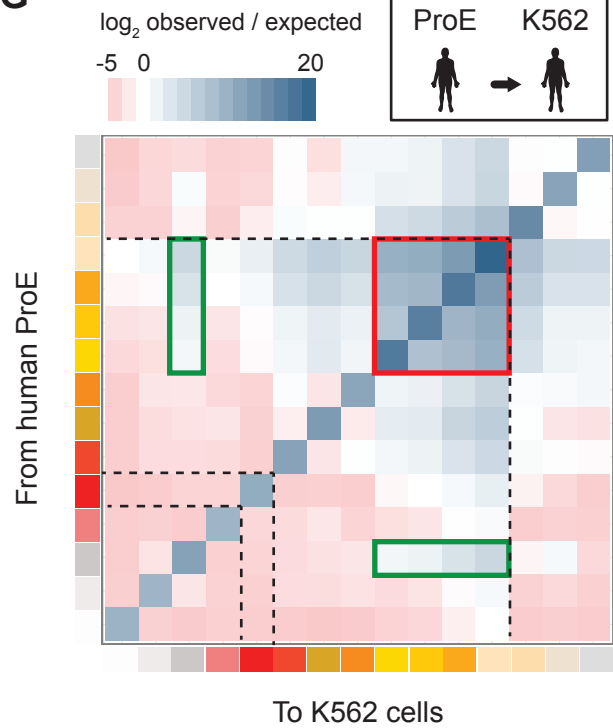

Supplement: S10 Fig — Chromatin state fold enrichment versus expected in K562 cells. Identical regions as investigated in Fig. 4B–F. Specifically, the regions are A) TF occupancy sites conserved between human and mouse, B) compensatory TF occupancy sites that are human-specific and proximal (+/- 5 kb) to a lost TF occupancy site during evolution, C) human-specific occupancy sites that are gained during evolution, D) top 10% of human occupancy sites based upon mapped reads, and E) mouse-specific occupancy sites that are lost during evolution. Overall, promoter and enhancer regulatory regions are decreasingly enriched for conserved, compensatory and human-specific, and finally mouse-specific occupancy sites in K562 cells. F) Similar to A), C), and E) except enrichment is calculated for mouse ProEs. An opposite pattern of enrichment is observed. G) Comparison of K562 cell chromatin states to human ProE chromatin states. Generally, states are highly conserved, although enhancer states/polycomb repressed regions transition to each other between cell-types more than expected by chance. Abbreviations used: ProEs, pro-erythroblasts. (PDF) [file pgen.1004890.s010.pdf]

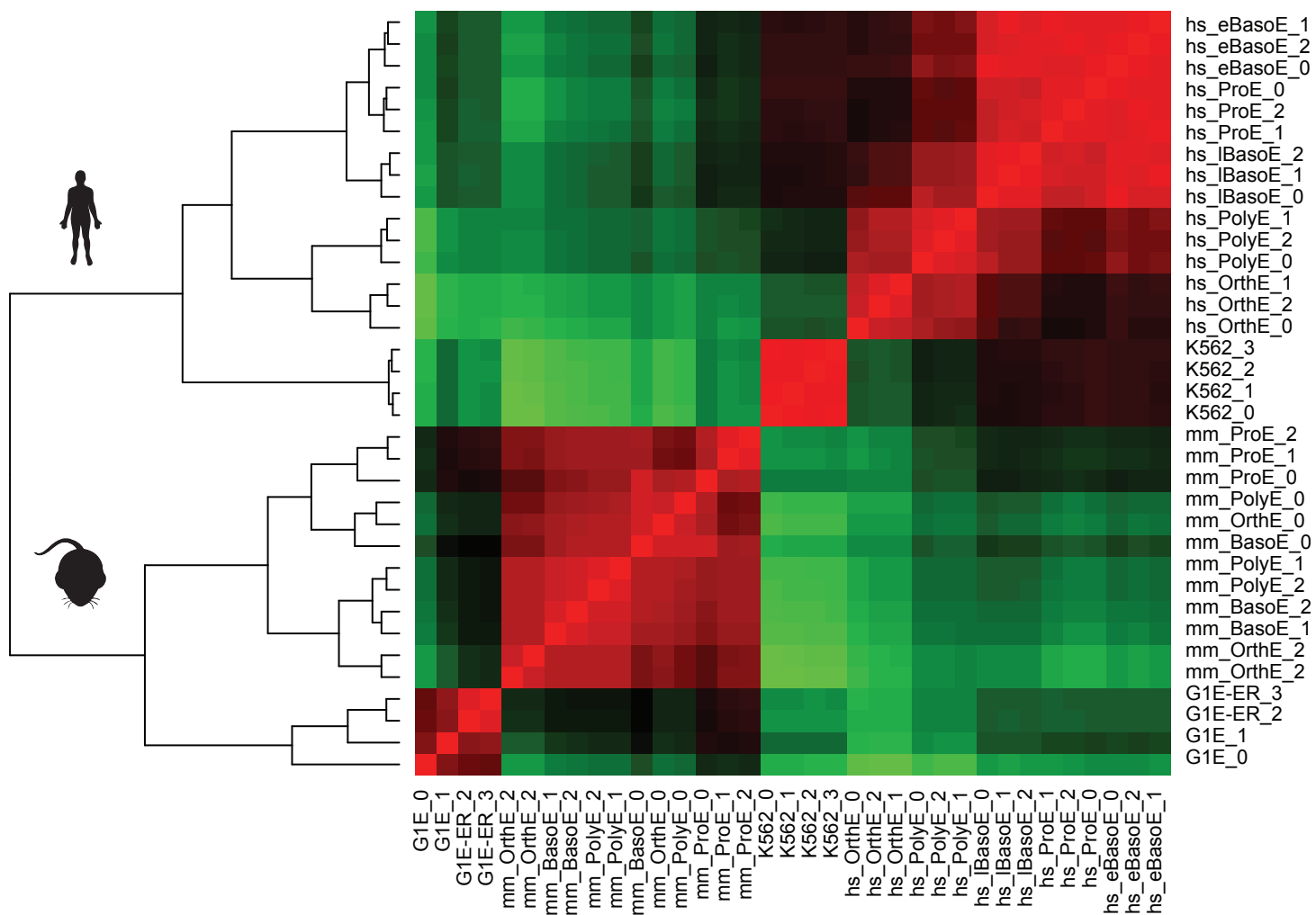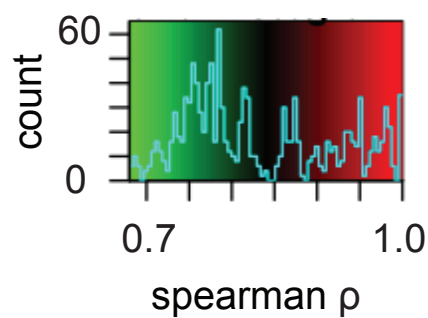

Supplement: S11 Fig — Inter- and intra-species gene expression. Spearman correlations for gene expression profiles between mouse and human primary ProEs, BasoEs (early and late, eBaso and lBaso), PolyEs, and OrthEs as well as erythroid cell lines, K562 and G1E, represented as a heatmap. The histogram in the legend represents the number of cells in the heatmap with a given correlation. Across all cell types, human and mouse gene expression profiles are more similar within- than between-species. Abbreviations used: ProEs, pro-erythroblasts; eBasoE, early basophilic erythroblasts; BasoE, basophilic erythroblasts; lBasoE, late basophilic erythroblasts; PolyE, polychromatic erythroblasts; OrthE, orthochromatic erythroblasts. (PDF) [file pgen.1004890.s011.pdf]

**A****GATA1**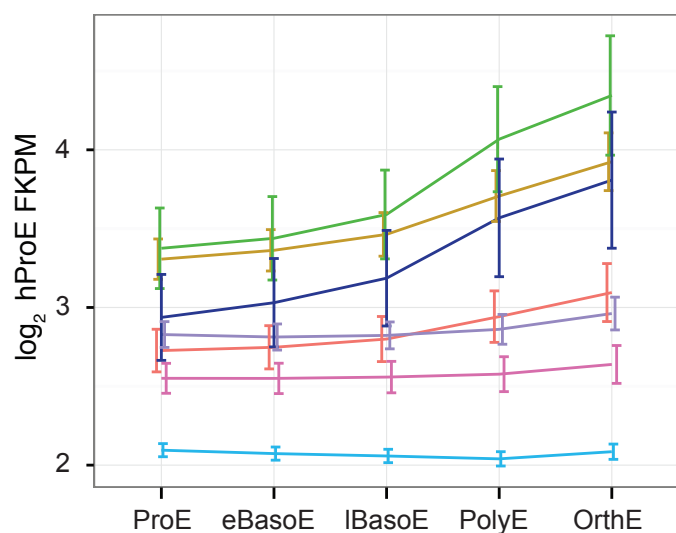**B****TAL1**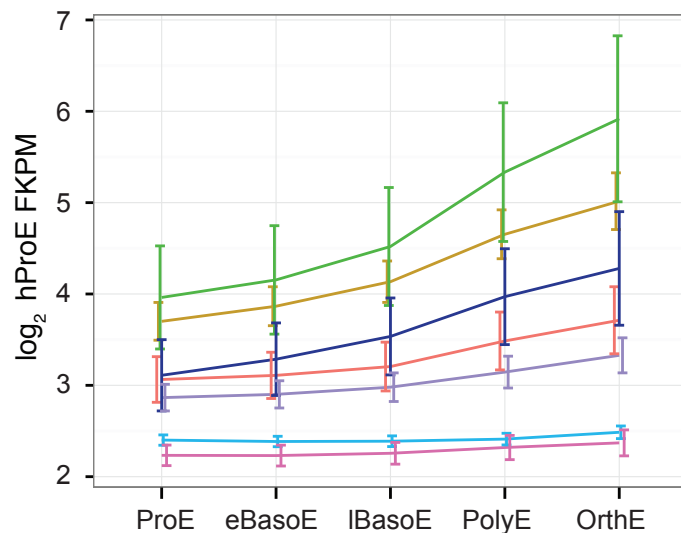**C****KLF1**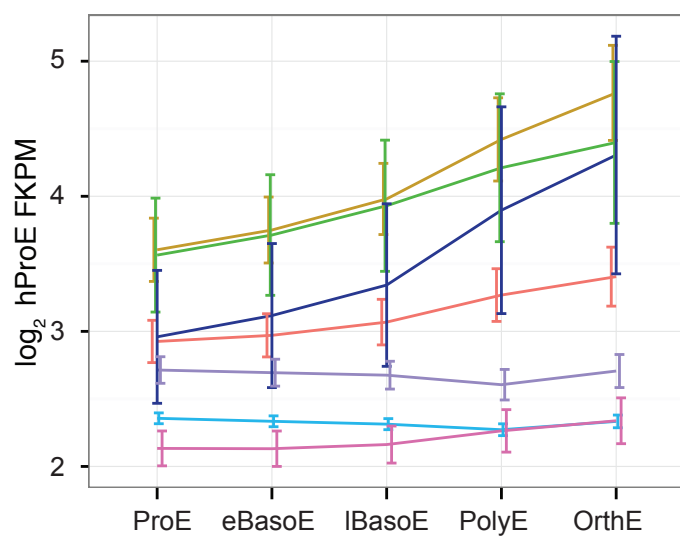**D****NFE2**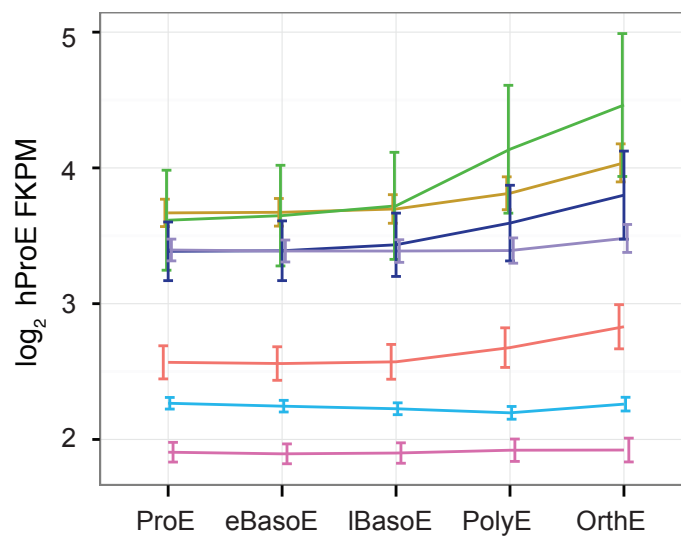**Legend**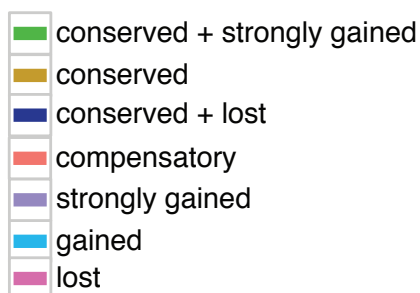

Supplement: S12 Fig — Gene expression for transcription factor conservation classes in human terminal erythroid differentiation. A)–D) Gene expression patterns during terminal erythroid differentiation based upon proximity to TF occupancy sites for varying TF occupancy conservation (defined in Fig. 4B–F and Fig. 5F–G) for each TF individually. Abbreviations used: ProEs, pro-erythroblasts; eBasoE, early basophilic erythroblasts; BasoE, basophilic erythroblasts; lBasoE, late basophilic erythroblasts; PolyE, polychromatic erythroblasts; OrthE, orthochromatic erythroblasts; FPKM, fragments of aligned reads per kilobase of transcript per million mapped reads. (PDF) [file pgen.1004890.s012.pdf]

**A**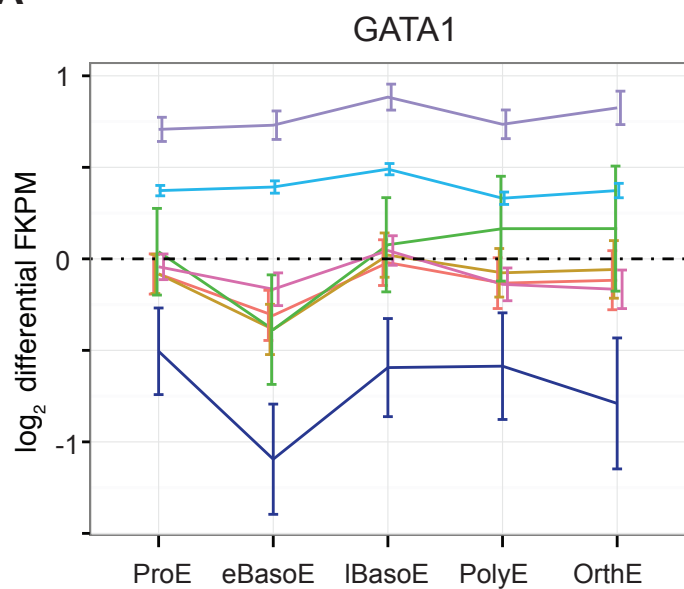**B**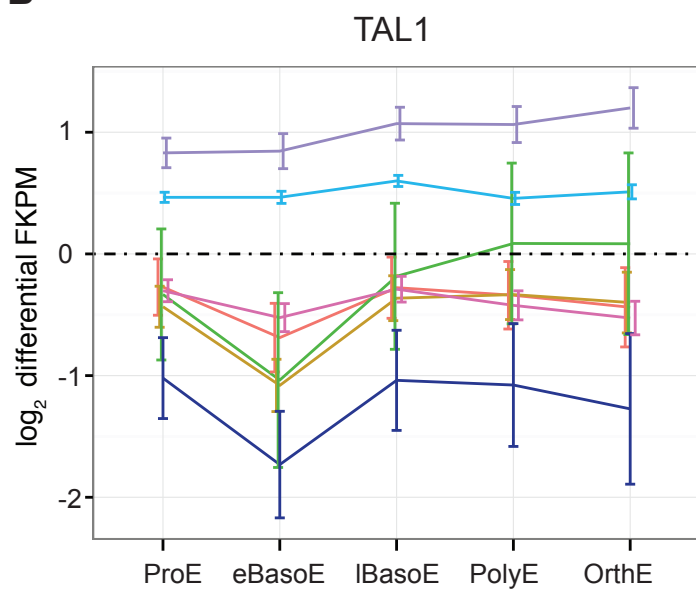**C**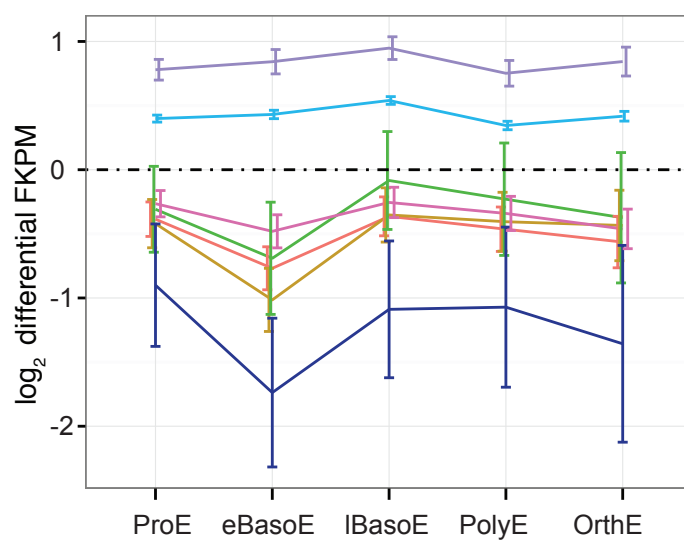**D**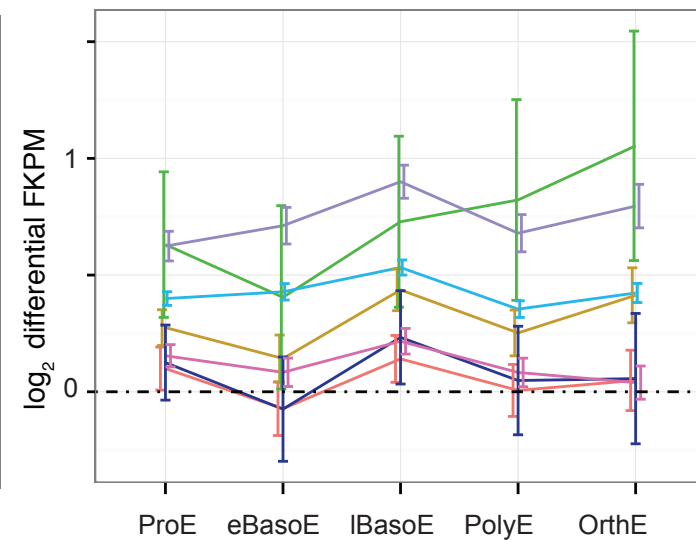

Legend

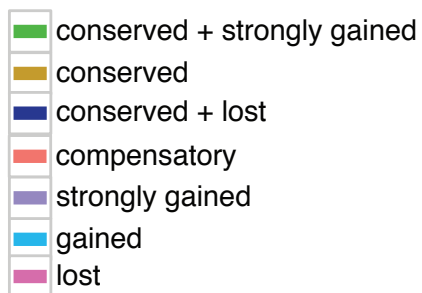

Supplement: S13 Fig — Cross species gene expression for transcription factor conservation classes. A)–D) Cross species gene expression patterns during terminal erythroid differentiation based upon proximity to TF occupancy sites for varying TF occupancy conservation (defined in Fig. 4B–F) for each TF individually. Positive corresponds to human-specific expression and negative correspond to mouse-specific expression. Abbreviations used: ProEs, pro-erythroblasts; eBasoE, early basophilic erythroblasts; BasoE, basophilic erythroblasts; lBasoE, late basophilic erythroblasts; PolyE, polychromatic erythroblasts; OrthE, orthochromatic erythroblasts; FPKM, fragments of aligned reads per kilobase of transcript per million mapped reads. (PDF) [file pgen.1004890.s013.pdf]

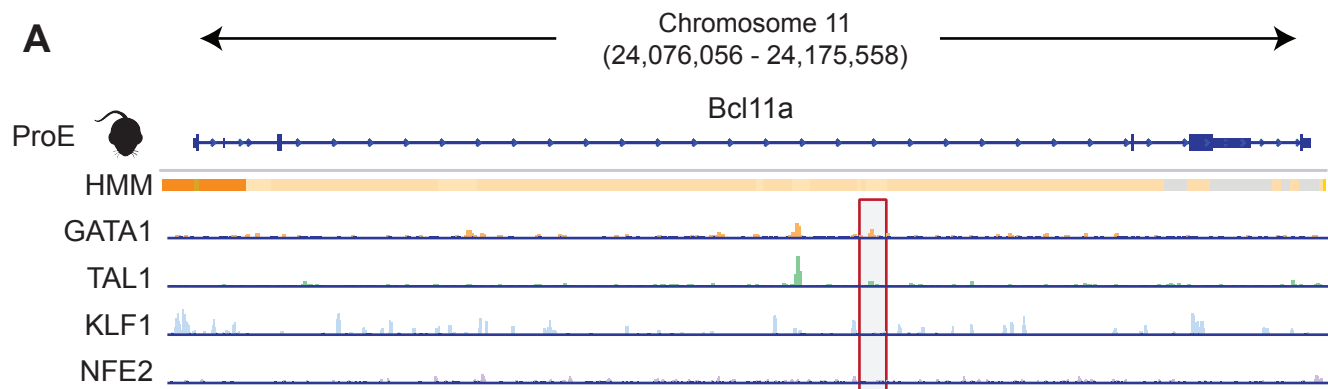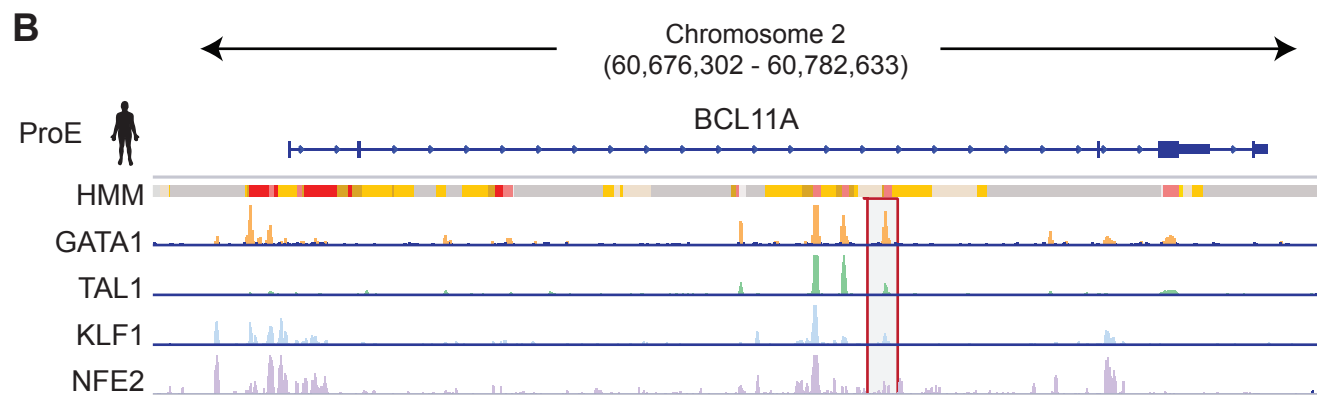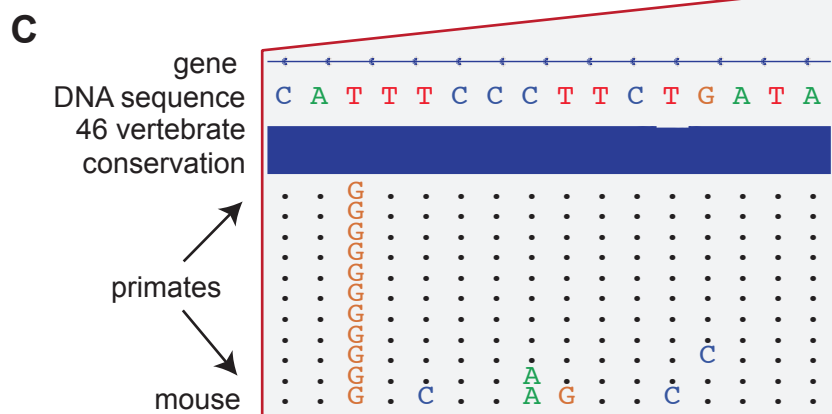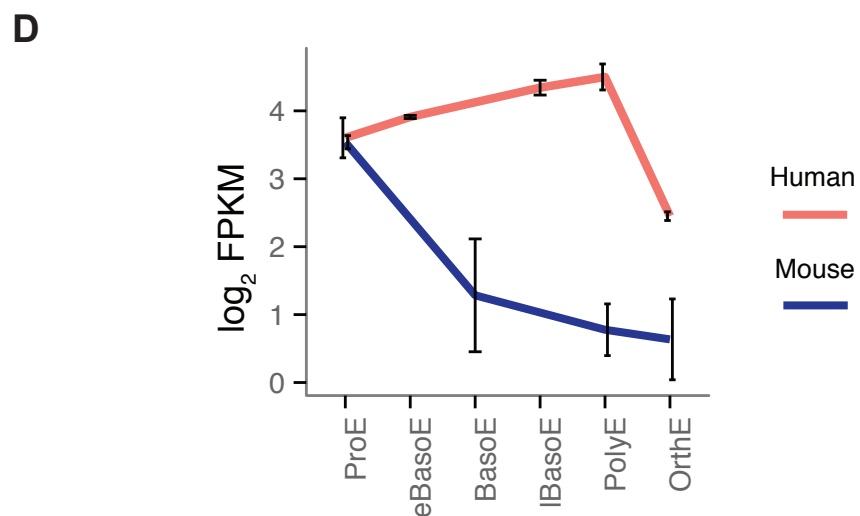

Supplement: S14 Fig — Applying the comparative epigenomics framework to BCL11A . A) Chromatin states learned from the HMM and TF intensities are shown for BCL11A in human ProEs. B) Chromatin states learned from the HMM and TF intensities are shown for Bcl11a in mouse ProEs. C) Genomic conservation of a GATA1/TAL1 binding site in an erythroid-specific enhancer across species. D) Gene expression patterns during terminal erythroid differentiation are shown for BCL11A by species. Error bars represent the mean +/- the standard deviation. Abbreviations used: ProE, pro-erythroblast; HMM, hidden markov model. (PDF) [file pgen.1004890.s014.pdf]

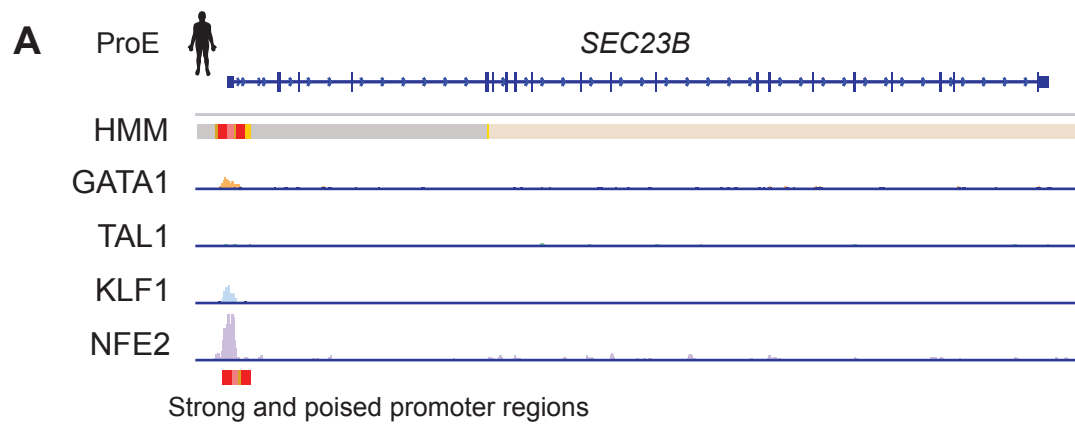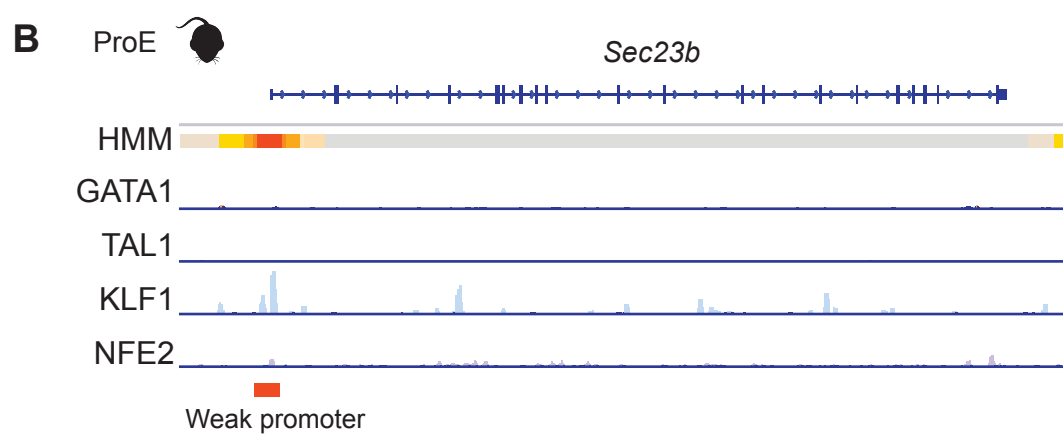

Supplement: S15 Fig — Comparative epigenomics of the SEC23B locus. Chromatin states learned from the HMM and TF intensities are shown for A) SEC23B in human and B) Sec23b in mouse. Legend for the HMM is shown in Fig. 4A. Some differences are observed between TF occupation and chromatin state, but gene expression across the orthologs is similar as shown in Fig. 7E. Abbreviations used: ProE, pro-erythroblast; HMM, hidden markov model. (PDF) [file pgen.1004890.s015.pdf]
