# Supplementary material for: Altered Chromatin Occupancy of Master Regulators Underlies Evolutionary Divergence in the Transcriptional Landscape of Erythroid Differentiation
Source: PLoS Genet. 2014 Dec 18;10(12):e1004890. doi: 10.1371/journal.pgen.1004890 (PMC4270484; doi:10.1371/journal.pgen.1004890)
Supplement: S5 Fig — Alternate estimates of transcription factor occupancy conservation. A) For each TF (GATA1, TAL1, KLF1, and NFE2), the estimated conservation rate (0–100%) of TF peaks between human and mouse ProEs are displayed. The lower bound represents the percentage of human peaks in orthologous genomic regions that are also present in mouse (left box). The middle estimate is the percentage of mouse peaks in orthologous genomic regions also present in human (middle box). The upper estimate is the same as the middle estimate, except that we first restricted to only the top 25% of peaks in mouse (left box). Conserved peaks are peaks that overlap in orthologous genomic regions, and compensatory peaks are peaks that are within +/- 5 kbs of the original peak. We expected to observe at least a small increase in the total percentage of conserved peaks for each TF, since stronger peak signals may be more indicative of function. Indeed, we observed a moderate increase (average increase of 18%) in percentage of conservation across each TF but also a significant drop (average 2.5-fold decrease) in the total number of conserved peaks. B) Similar to A), except that that the estimated number of conserved peaks are displayed instead of the percentage. Please note that this is the total number of overlapping mapped peaks, and not the total number of mapped peaks. Abbreviations used: ProE, pro-erythroblast. (PDF) [file pgen.1004890.s005.pdf]

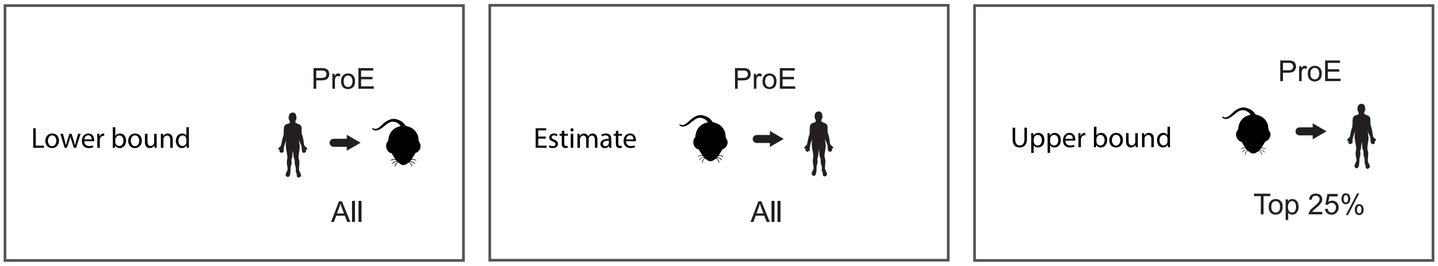

A

	conserved			compensatory		
	Lower bound	Estimate	Upper bound	Lower bound	Estimate	Upper bound
GATA1	4.9%	25.5%	36.9%	11.8%	48.9%	58.0%
TAL1	6.6%	21.7%	40.7%	11.6%	33.7%	50.2%
KLF1	22.2%	60.4%	82.1%	26.1%	78.2%	92.0%
NFE2	7.5%	19.6%	43.7%	18.5%	37.3%	64.2%

B

	Lower bound	Estimate	Upper bound	Lower bound	Estimate	Upper bound
GATA1	1483	1747	669	3683	3216	1052
TAL1	711	730	382	1246	1135	471
KLF1	7616	7184	2541	12132	9302	2849
NFE2	2194	2079	1129	6586	3958	1659
