# Supplementary material for: Altered Chromatin Occupancy of Master Regulators Underlies Evolutionary Divergence in the Transcriptional Landscape of Erythroid Differentiation
Source: PLoS Genet. 2014 Dec 18;10(12):e1004890. doi: 10.1371/journal.pgen.1004890 (PMC4270484; doi:10.1371/journal.pgen.1004890)
Supplement: S1 Table — Raw ChIP-seq datasets. ChIP-seq datasets for each cell-type analyzed in this manuscript. Raw data was available on either NCBI GEO or the ENCODE website. Abbreviations used: ChIP-seq, chromatin immunoprecipitation high-throughput sequencing. (PDF) [file pgen.1004890.s016.pdf]

	Mouse		Human	
	ProE	G1E	ProE	K562
**GATA1**	2,3,4,10	4,8,10	1,6,9,13	9,11
**TAL1**	4,10	4,10	1,6,13	9
**KLF1**	5		6	9
**NFE2**	2		6	9
**H3K4me3**	4,12	4,10	6	9
**H3K27me3**	4,12	4,10	1	9
**H3K4me1**	4	4	7	9
**H3K4me2**	12		7	9
**H3K36me3**	12	4	7	9
**H3K9ac**	12		7	9

#	Citation	GEO ID
1	Pinello et al, PNAS 2014	GSE52924
2	Hughes et al, Nature Genetics 2014	GSE47492
3	May et al, Cell Stem Cell 2013	GSE50406
4	Mouse ENCODE	GSE36028
5	Pilon et al, Blood 2011	GSE48020
6	Su et al, J Biol Chem 2013	GSE43626
7	Xu et al., Dev Cell 2012	GSE36985
8	Kadauke et al., Cell 2012	GSE36589
9	ENCODE	GSE31477
10	Wu et al., Genome Research 2011	GSE30142
11	Fujiwara et al., Mol Cell 2009	GSE18868
12	Wong et al., Blood 2011	GSE27893
13	Hu et al., Genome Research 2011	GSE26501
