# Supplementary material for: Altered Chromatin Occupancy of Master Regulators Underlies Evolutionary Divergence in the Transcriptional Landscape of Erythroid Differentiation
Source: PLoS Genet. 2014 Dec 18;10(12):e1004890. doi: 10.1371/journal.pgen.1004890 (PMC4270484; doi:10.1371/journal.pgen.1004890)
Supplement: S2 Table — ChIP-seq summary statistics. Summary statistics for each ChIP-seq dataset used in the analysis. Abbreviations used: none. (PDF) [file pgen.1004890.s017.pdf]

Identifier	Cell type	Factor	Replicate	Raw reads	Aligned reads	Number of peaks*
ENCODE	K562	GATA1	Rep 1	7855375	5628646	24578
ENCODE	K562	GATA1	Rep 2	12668283	10330308	
GSE18868	K562	GATA1	Rep 1	1478403	868302	
GSE18868	K562	GATA1	Rep 2	12668283	10330308	
GSE18868	K562	GATA1	Rep 3	2307595	1671964	
GSE18868	K562	GATA1	Rep 4	4069377	3088380	
ENCODE	K562	TAL1	Rep 1	32146960	26541327	25309
ENCODE	K562	TAL1	Rep 2	21844596	17764539	
ENCODE	K562	NFE2	Rep 1	6808336	5359885	6008
ENCODE	K562	NFE2	Rep 2	6395318	5099213	
GSE26320	K562	H3K4me1	Rep 1	17848560	8157362	
GSE26320	K562	H3K4me1	Rep 2	18105919	6319250	
GSE26320	K562	H3K4me2	Rep 1	16027507	11235270	
GSE26320	K562	H3K4me2	Rep 2	18105919	6319250	
ENCODE	K562	H3K4me3	Rep 1	14782249	7239860	
ENCODE	K562	H3K4me3	Rep 2	25272278	14033165	
ENCODE	K562	H3K27me3	Rep 1	19297190	10247229	
ENCODE	K562	H3K27me3	Rep 2	22830589	10515543	
GSE26320	K562	H3K36me3	Rep 1	26973698	12064926	
GSE26320	K562	H3K36me3	Rep 2	17501267	8931276	
GSE26320	K562	H3K9ac	Rep 1	23461826	13425993	
GSE26320	K562	H3K9ac	Rep 2, Read 1	11105594	2960101	
GSE26320	K562	H3K9ac	Rep 2, Read 2	13453306	6998259	
ENCODE	hProE	GATA1	Rep 1	20358537	15052483	50543
ENCODE	hProE	GATA1	Rep 1	22918252	13389066	
GSE26501	hProE	GATA1	Rep 1	18201969	14557864	
GSE52924	hProE	GATA1	Rep 1	125754149	103668057	
GSE43626	hProE	GATA1	Rep 1	27642243	23331425	
GSE26501	hProE	TAL1	Rep 1	15373663	11695167	17827
GSE52924	hProE	TAL1	Rep 1	129960043	83604587	
GSE43626	hProE	KLF1	Rep 1	31419057	27162194	48429
GSE43626	hProE	NFE2	Rep 1	26992380	23170446	42435
GSE36985	hProE	H3K4me1	Rep 1	8691775	2315420	
GSE36985	hProE	H3K4me2	Rep 1	10990840	3388775	
GSE43626	hProE	H3K4me3	Rep 1	28380798	22727258	
GSE52924	hProE	H3K27me3	Rep 1	155656072	143818225	
GSE36985	hProE	H3K36me3	Rep 1	6421505	2576254	
GSE36985	hProE	H3K9ac	Rep 1	14048449	4695040	
GSE30142	G1E	TAL1	Rep 2	24252830	19778393	
Mouse ENCODE	G1E	TAL1	Rep 1	14965148	11574453	
Mouse ENCODE	G1E	TAL1	Rep 2	24252830	19778393	
Mouse ENCODE	G1E	H3K4me1	Rep 1	34088173	30203973	
Mouse ENCODE	G1E	H3K4me1	Rep 2	49615724	42092797	
GSE30142	G1E	H3K4me3	Rep 1, Read 1	17566489	15687368	
GSE30142	G1E	H3K4me3	Rep 1, Read 2	17767708	15868604	
GSE30142	G1E	H3K4me3	Rep 2	95699196	73827183	
GSE30142	G1E	H3K27me3	Rep 1, Read 1	11128155	8417401	
GSE30142	G1E	H3K27me3	Rep 1, Read 2	11195913	8504046	
GSE30142	G1E	H3K27me3	Rep 2	16593566	14331684	
Mouse ENCODE	G1E	H3K36me3	Rep 1	116403361	104048347	
Mouse ENCODE	G1E	H3K36me3	Rep 2	86998117	76296872	
Mouse ENCODE	G1E-ER	GATA1	Rep 1	36627078	30056277	
Mouse ENCODE	G1E-ER	GATA1	Rep 2	125843327	93523439	
Mouse ENCODE	G1E-ER	GATA1	Rep 3	33124216	26059529	
Mouse ENCODE	G1E-ER	GATA1	Rep 4	120431030	94419653	
GSE36589	G1E-ER	GATA1	Rep 1	33124216	26059529	
GSE30142	G1E-ER	GATA1	Rep 1	120431030	94419653	
GSE30142	G1E-ER	TAL1	Rep 1, Read 1	6009741	2016973	
GSE30142	G1E-ER	TAL1	Rep 1, Read 2	8551081	2547387	
GSE30142	G1E-ER	TAL1	Rep 1, Read 3	9358292	2867385	
GSE30142	G1E-ER	TAL1	Rep 2	11280758	9175665	
Mouse ENCODE	G1E-ER	TAL1	Rep 1	23919114	7431745	
Mouse ENCODE	G1E-ER	TAL1	Rep 2	11280758	9175665	
Mouse ENCODE	G1E-ER	H3K4me1	Rep 1	24839073	22108130	
Mouse ENCODE	G1E-ER	H3K4me1	Rep 2	115103341	95437238	

GSE30142	G1E-ER	H3K4me3	Rep 1	11447979	9999421	
GSE30142	G1E-ER	H3K4me3	Rep 2	103282886	78798006	
GSE30142	G1E-ER	H3K27me3	Rep 1, Read 1	9280578	7304020	
GSE30142	G1E-ER	H3K27me3	Rep 1, Read 2	12701110	9849294	
GSE30142	G1E-ER	H3K27me3	Rep 2	115996679	96939539	
Mouse ENCODE	G1E-ER	H3K36me3	Rep 1	95902219	86896610	
Mouse ENCODE	G1E-ER	H3K36me3	Rep 2	100910289	86401428	
GSE50406	mProE	GATA1	Rep 1	25021545	19847097	10035
GSE30142	mProE	GATA1	Rep 1	37667511	28933586	
GSE30142	mProE	GATA1	Rep 2	91981872	68232667	
GSE47492	mProE	GATA1	Rep 1	37251582	16215040	
GSE30142	mProE	TAL1	Rep 1	37247380	30722965	5187
GSE30142	mProE	TAL1	Rep 2	119633592	85201789	
Mouse ENCODE	mProE	TAL1	Rep 1	28646	23780	
Mouse ENCODE	mProE	TAL1	Rep 2	2349774	1673228	
Mouse ENCODE	mProE	TAL1	Rep 3	20418391	15956785	
GSE48020	mProE	KLF1	Rep 1	17941425	12568777	13496
GSE48020	mProE	KLF1	Rep 2	17167234	12536688	
GSE47492	mProE	NFE2	Rep 1	36111826	17851636	23445
Mouse ENCODE	mProE	H3K4me1	Rep 1	90552604	76730939	
Mouse ENCODE	mProE	H3K4me1	Rep 2	106280129	91773476	
GSE27893	mProE	H3K4me2	Rep 1	19404356	12292023	
GSE27893	mProE	H3K4me3	Rep 2	3065525	2684815	
GSE27893	mProE	H3K27me3	Rep 1	3584413	2999528	
Mouse ENCODE	mProE	H3K36me3	Rep 1	91149357	77249035	
Mouse ENCODE	mProE	H3K36me3	Rep 2	103228393	91410435	
GSE27893	mProE	H3K36me3	Rep 1	21214582	5042176	
GSE27893	mProE	H3K9ac	Rep 1	2629538	2083420	

*Peaks were determined by pooling all replicates
