# Supplementary material for: Altered Chromatin Occupancy of Master Regulators Underlies Evolutionary Divergence in the Transcriptional Landscape of Erythroid Differentiation
Source: PLoS Genet. 2014 Dec 18;10(12):e1004890. doi: 10.1371/journal.pgen.1004890 (PMC4270484; doi:10.1371/journal.pgen.1004890)
Supplement: S3 Table — Values for correlation matrices. Spearman correlation matrices for H3K4me3, H3K27me3, and H3K4me1. Matrices shown examples of biological replicate correlations for each mark as well as low correlations between K562 and G1E cell types. Abbreviations used: ChIP-seq, chromatin immunoprecipitation followed by high-throughput sequencing. (PDF) [file pgen.1004890.s018.pdf]

H3K4me1	hProE_1	K562_1	K562_2	mProE_1	mProE_2	G1E_1	G1E_2	G1E_3	G1E_4
hProE_1	1	0.44538032	0.40720437	0.23993175	0.23690307	0.25757421	0.25362134	0.20543981	0.24222975
K562_1	0.44538032	1	0.86488142	0.34648703	0.34508533	0.3612124	0.32956468	0.325552	0.32297527
K562_2	0.40720437	0.86488142	1	0.275412	0.27548324	0.3028167	0.24011791	0.29379891	0.2464952
mProE_1	0.23993175	0.34648703	0.275412	1	0.98159754	0.68774257	0.714893	0.61645171	0.66352365
mProE_2	0.23690307	0.34508533	0.27548324	0.98159754	1	0.6878775	0.71667748	0.61762315	0.67769711
G1E_1	0.25757421	0.3612124	0.3028167	0.68774257	0.6878775	1	0.88147623	0.8476441	0.82750288
G1E_2	0.25362134	0.32956468	0.24011791	0.714893	0.71667748	0.88147623	1	0.72047177	0.8528431
G1E_3	0.20543981	0.325552	0.29379891	0.61645171	0.61762315	0.8476441	0.72047177	1	0.81583382
G1E_4	0.24222975	0.32297527	0.2464952	0.66352365	0.67769711	0.82750288	0.8528431	0.81583382	1

H3K4me2	hProE_1	K562_1	K562_2	mProE_1
hProE_1	1	0.62812231	0.62505586	0.58966148
K562_1	0.62812231	1	0.90460885	0.75327777
K562_2	0.62505586	0.90460885	1	0.66748603
mProE_1	0.58966148	0.75327777	0.66748603	1

H3K4me3	hProE_1	hProE_2	K562_1	K562_2	mProE_1	G1E_1	G1E_2	G1E_3	G1E_4
hProE_1	1	0.88517291	0.87349669	0.88157575	0.79801321	0.78375024	0.75347635	0.77069668	0.77562164
hProE_2	0.88517291	1	0.79873598	0.80693663	0.79581235	0.76557693	0.76296142	0.77050473	0.76908548
K562_1	0.87349669	0.79873598	1	0.98214396	0.76175205	0.74661901	0.70422534	0.71994355	0.72823252
K562_2	0.88157575	0.80693663	0.98214396	1	0.7722681	0.75531373	0.71740366	0.73056185	0.74034591
mProE_1	0.79801321	0.79581235	0.76175205	0.7722681	1	0.91835676	0.9107748	0.9179498	0.91929749
G1E_1	0.78375024	0.76557693	0.74661901	0.75531373	0.91835676	1	0.94619169	0.97955154	0.96404341
G1E_2	0.75347635	0.76296142	0.70422534	0.71740366	0.9107748	0.94619169	1	0.95459533	0.97301623
G1E_3	0.77069668	0.77050473	0.71994355	0.73056185	0.9179498	0.97955154	0.95459533	1	0.97973358
G1E_4	0.77562164	0.76908548	0.72823252	0.74034591	0.91929749	0.96404341	0.97301623	0.97973358	1

H3K9ac	hProE_1	K562_1	K562_2	mProE_1
hProE_1	1	0.85835736	0.78312696	0.74960017
K562_1	0.85835736	1	0.93651642	0.72780375
K562_2	0.78312696	0.93651642	1	0.67336663
mProE_1	0.74960017	0.72780375	0.67336663	1

H3K27me3	hProE_1	hProE_2	K562_1	K562_2	mProE_1	G1E_1	G1E_2	G1E_3	G1E_4
hProE_1	1	0.93217832	0.46601432	0.41414412	0.65833608	0.22978987	0.26543957	0.4050342	0.26858767
hProE_2	0.93217832	1	0.44367328	0.43333257	0.68602864	0.24241656	0.29121533	0.44244692	0.2954955
K562_1	0.46601432	0.44367328	1	0.68822483	0.29110313	0.29251776	0.30097259	0.26532314	0.27318735
K562_2	0.41414412	0.43333257	0.68822483	1	0.29224406	0.26455464	0.29544629	0.28437543	0.26970712
mProE_1	0.65833608	0.68602864	0.29110313	0.29224406	1	0.30244662	0.35508981	0.58333289	0.37673526
G1E_1	0.22978987	0.24241656	0.29251776	0.26455464	0.30244662	1	0.90969619	0.70118918	0.82464884
G1E_2	0.26543957	0.29121533	0.30097259	0.29544629	0.35508981	0.90969619	1	0.7870802	0.87009417
G1E_3	0.4050342	0.44244692	0.26532314	0.28437543	0.58333289	0.70118918	0.7870802	1	0.86166076
G1E_4	0.26858767	0.2954955	0.27318735	0.26970712	0.37673526	0.82464884	0.87009417	0.86166076	1

H3K36me3	hProE_1	K562_1	K562_2	mProE_1	G1E_1	G1E_2	G1E_3	G1E_4
hProE_1	1	0.27701569	0.29362228	0.19971101	0.21122358	0.19543912	0.21187062	0.19145014
K562_1	0.27701569	1	0.7683953	0.19829812	0.21186443	0.20081486	0.2181758	0.19241206
K562_2	0.29362228	0.7683953	1	0.18958665	0.20590441	0.18634527	0.20996608	0.17449626
mProE_1	0.19971101	0.19829812	0.18958665	1	0.67431202	0.65316545	0.78818393	0.73931716
G1E_1	0.21122358	0.21186443	0.20590441	0.67431202	1	0.95478493	0.90579486	0.88411235
G1E_2	0.19543912	0.20081486	0.18634527	0.65316545	0.95478493	1	0.88630176	0.88806314
G1E_3	0.21187062	0.2181758	0.20996608	0.78818393	0.90579486	0.88630176	1	0.9488205
G1E_4	0.19145014	0.19241206	0.17449626	0.73931716	0.88411235	0.88806314	0.9488205	1
