# Supplementary material for: Altered Chromatin Occupancy of Master Regulators Underlies Evolutionary Divergence in the Transcriptional Landscape of Erythroid Differentiation
Source: PLoS Genet. 2014 Dec 18;10(12):e1004890. doi: 10.1371/journal.pgen.1004890 (PMC4270484; doi:10.1371/journal.pgen.1004890)
Supplement: S4 Table — GATA1, TAL1, and KLF1 conserved regions. Coordinates in hg19 for human ProEs. Regions are centered around KLF1 peaks lifted over from mouse ProEs. Abbreviations used: ProE, pro-erythroblast. (PDF) [file pgen.1004890.s019.pdf]

Chromosome	Loci Start	Loci End	Gene Symbol	Strand	GATA1, KLF1, TAL1 peak distance to TSS
chr13	41632680	41636735	TRNA_Glu	-	238
chr12	22198747	22202317	CMAS	+	1374
chr12	50440483	50446240	ACCN2	+	-8125
chr12	56692622	56696794	CS	-	-533
chr12	62652370	62656346	USP15	+	238
chr12	106694105	106697736	TCP11L2	+	-660
chr12	114368766	114372864	RBM19	-	33361
chr11	907269	912881	AP2A2	+	-15733
chr11	5244178	5249713	HBB	-	1356
chr11	5295386	5298551	HBG2	-	370043
chr11	5304753	5307936	HBG2	-	360667
chr11	10476289	10479151	AMPD3	+	5853
chr11	34458739	34462077	CAT	+	-63
chr11	44597719	44605325	CD82	+	14382
chr11	61683721	61689056	RAB3IL1	-	1353
chr11	61727928	61742147	FTH1	-	95
chr11	65336112	65347994	FAM89B	+	2234
chr11	65402739	65406875	SIPA1	+	-770
chr11	66681648	66683994	PC	-	43026
chr10	71991365	71994264	PPA1	-	376
chr10	104401322	104409280	TRIM8	+	1050
chr17	1632495	1634896	WDR81	+	13879
chr17	19911589	19915730	SPECC1	+	1011
chr17	29149812	29153461	CRLF3	-	142
chr17	40610201	40612727	ATP6V0A1	+	603
chr17	40849580	40852445	CNTNAP1	+	16381
chr17	42342228	42347879	SLC4A1	-	449
chr17	42356784	42362865	SLC4A1	-	-14322
chr17	47324889	47328647	FLJ40194	+	1164
chr17	47815455	47819429	FAM117A	-	48495
chr17	71302746	71309525	CDC42EP4	-	2008
chr17	74377479	74383657	SPHK1	+	7827
chr17	75135006	75138612	SCARNA16	+	51421
chr17	76117392	76131118	TMC6	-	4233
chr16	154075	156349	NPRL3	-	33485
chr16	615265	621966	NHLRC4	+	1584
chr16	4849493	4855702	ROGDI	-	77
chr16	19531443	19534095	GDE1	-	681
chr16	56457222	56460596	AMFR	-	535

chr16	67833392	67837965	RANBP10	-	4877
chr16	67879388	67882390	NUTF2	+	71
chr16	85403214	85413482	LOC727710	-	-71346
chr16	85585991	85593834	KIAA0182	+	-55116
chr16	88517364	88527254	ZFPM1	+	2296
chr16	88556584	88559682	ZFPM1	+	38120
chr16	88572993	88587310	ZFPM1	+	60138
chr16	88587347	88594547	ZFPM1	+	70934
chr16	89069305	89073292	CBFA2T3	-	-27794
chr15	40718486	40720665	IVD	+	21890
chr15	43508881	43514635	EPB42	-	1565
chr15	64123795	64127792	HERC1	-	354
chr15	64441388	64447245	SNX22	+	401
chr15	66096344	66098840	TRNA_Gln	-	63879
chr15	66970906	66974277	BC016970	+	98064
chr15	78555020	78559460	DNAJA4	+	754
chr15	90756194	90757609	SEMA4B	+	28750
chr14	37639959	37644515	LOC100129794	+	1007
chr14	50807359	50811743	CDKL1	-	73628
chr14	59653425	59657777	DAAM1	+	203
chr14	65307110	65315282	SPTB	-	35405
chr14	65344236	65348407	SPTB	-	280
chr14	72946178	72948517	RGS6	+	548531
chr14	102969605	102972304	TECPR2	+	141655
chr19	1631522	1634386	TCF3	-	19374
chr19	5798135	5800719	DUS3L	-	-8178
chr19	10395282	10407435	ICAM5	+	704
chr19	10653159	10656061	ATG4D	+	-36
chr19	11491102	11496514	EPOR	-	1210
chr19	12910128	12914113	PRDX2	-	574
chr19	12993965	13000544	KLF1	-	763
chr19	13055217	13060994	RAD23A	+	1452
chr19	34744796	34747143	KIAA0355	+	514
chr19	40968776	40973470	BLVRB	-	602
chr19	47743905	47748722	CCDC9	+	-13417
chr19	49374512	49383216	PPP1R15A	+	3216
chr18	43731737	43734674	C18orf25	+	-20782
chr18	55249616	55257866	FECH	-	228
chrx	134653963	134657421	DDX26B	+	1138
chr22	19919421	19924049	TXNRD2	-	7624
chr22	39539386	39543581	CBX7	-	7055
chr20	3746561	3750083	C20orf27	-	130
chr20	17549459	17552515	DSTN	+	389

chr20	25034489	25041165	ACSS1	-	991
chr20	30261081	30267614	BCL2L1	-	46309
chr20	30793111	30796958	PLAGL2	-	512
chr21	34567881	34573764	C21orf54	-	-28281
chr7	1093718	1096584	GPR146	+	207
chr7	100129726	100133901	AGFG2	+	-5020
chr7	100489776	100496433	ACHE	-	611
chr7	129597419	129600024	JA611275	+	4122
chr7	138774725	138778437	ZC3HAV1	-	17884
chr7	155086689	155092044	INSIG1	+	-119
chr6	30127458	30131215	TRIM10	-	-625
chr6	37069440	37073210	PIM1	+	-66596
chr6	37076548	37079338	PIM1	+	-59978
chr6	37633991	37636429	MDGA1	-	30556
chr6	43594460	43600365	MAD2L1BP	+	134
chr6	52931630	52934269	FBXO9	+	3154
chr5	67660714	67665145	PIK3R1	+	151346
chr5	102593800	102597284	C5orf30	+	1101
chr5	153575638	153581078	GALNT10	+	8064
chr5	176851976	176856959	GRK6	+	781
chr5	179226418	179235055	SQSTM1	+	-2651
chr4	2844066	2847833	ADD1	+	366
chr4	56914398	56917049	CEP135	+	100687
chr4	90756228	90759997	SNCA	-	1335
chr4	145057858	145064765	GYPA	-	593
chr4	153855715	153860325	BC039551	-	-31
chr4	185664316	185670149	ACSL1	-	79983
chr3	16378338	16381526	RFTN1	-	175290
chr3	49393147	49397963	GPX1	-	236
chr3	49708018	49714909	APEH	+	29
chr3	49839419	49845906	UBA7	-	8729
chr3	50269424	50277341	GNAI2	+	9659
chr3	50328268	50331629	IFRD2	-	401
chr3	98311202	98314270	CPOX	-	-281
chr3	183732807	183738233	ABCC5	-	207
chr3	195918563	195924786	ZDHHC19	-	16626
chr2	113440483	113445370	SLC20A1	+	39493
chr2	178127696	178132016	NFE2L2	-	127563
chr2	190442836	190450899	SLC40A1	-	-1330
chr2	220081615	220085542	ABCB6	-	1596
chr2	220141947	220145651	DNAJB2	+	-240
chr2	228315199	228317825	AGFG1	+	-20375
chr1	15735301	15739864	EFHD2	+	1192

chr1	25737610	25742104	RHCE	-	16826
chr1	25745417	25748799	RHCE	-	9575
chr1	26867583	26873461	RPS6KA1	+	14274
chr1	29208034	29215385	EPB41	+	-1893
chr1	44494724	44499056	SLC6A9	-	244
chr1	44499083	44503673	KLF17	+	-12651
chr1	45475202	45481141	UROD	+	367
chr1	47644074	47649162	PDZK1IP1	-	9153
chr1	114455078	114461613	DCLRE1B	+	10431
chr1	116974087	116977208	ATP1A1OS	-	-14403
chr1	118146873	118159122	FAM46C	+	4394
chr1	203258231	203260577	LOC730227	-	15049
chr1	203271762	203277004	LOC730227	-	70
chr1	205223982	205226731	TMCC2	+	28319
chr1	220079246	220082187	SLC30A10	-	51273
chr1	224689731	224692981	AB586698	-	-85273
chr1	225612234	225617276	LBR	-	1802
chr1	226185358	226189261	C1orf55	-	-243
chr1	247093871	247096543	AHCTF1	-	-481
chr1	248017162	248023067	TRIM58	+	-386
chr9	37902679	37906148	BC021061	-	7725
chr9	92217635	92223022	GADD45G	+	402
chr9	100263098	100268024	TMOD1	+	2100
chr9	125093351	125096346	MRRF	+	67967
chr9	127043964	127046435	NEK6	+	25315
chr8	20090833	20094377	LZTS1	-	20198
chr8	110373703	110376501	PKHD1L1	+	397
chr8	125482697	125489252	CR933665	-	830
chr8	142316694	142319863	LOC731779	+	-32369
