# Supplementary material for: Altered Chromatin Occupancy of Master Regulators Underlies Evolutionary Divergence in the Transcriptional Landscape of Erythroid Differentiation
Source: PLoS Genet. 2014 Dec 18;10(12):e1004890. doi: 10.1371/journal.pgen.1004890 (PMC4270484; doi:10.1371/journal.pgen.1004890)
Supplement: S5 Table — Chromatin state functional enrichment for HMM. Values corresponding to the heatmaps shown in Fig. 4A. Abbreviations used: none. (PDF) [file pgen.1004890.s020.pdf]

state	H3K36me3*	H3K4me1*	H3K4me2*	H3K4me3*	H3K9ac*	H3K27me3*
1	0.74	0.02	0.00	0.00	0.00	0.00
2	0.04	0.01	0.00	0.00	0.00	0.00
3	0.92	0.82	0.02	0.00	0.01	0.00
4	0.94	0.98	0.93	0.36	0.33	0.01
5	0.04	0.96	0.74	0.17	0.20	0.01
6	0.02	0.27	0.18	0.03	0.12	0.00
7	0.03	0.70	0.00	0.01	0.01	0.01
8	0.05	0.96	1.00	0.93	0.76	0.04
9	0.01	0.17	0.63	0.61	0.08	0.02
10	0.04	0.05	0.96	0.89	0.93	0.00
11	0.00	0.04	0.72	1.00	0.94	0.03
12	0.03	0.44	0.65	0.45	0.08	0.93
13	0.00	0.00	0.00	0.00	0.00	0.07
14	0.00	0.00	0.00	0.00	0.00	0.68
15	0.00	0.00	0.00	0.00	0.00	0.00

*observation frequency

state	Genome %	CpG Island*	Exons*	Gene body*	TES*	TSS*	Promoter*	Lamin B1*
1	0.06	0.97	10.62	2.38	4.72	0.98	0.75	0.28
2	10.19	0.40	2.91	2.12	2.73	0.41	0.94	0.31
3	0.00	0.00	10.28	2.20	0.00	0.00	0.85	0.42
4	0.00	0.00	3.24	1.02	14.88	0.00	3.20	0.39
5	0.03	0.83	2.38	1.80	2.07	1.45	3.11	0.25
6	0.99	0.70	1.80	1.69	2.48	1.57	4.30	0.35
7	0.20	0.14	1.69	1.87	2.04	1.05	1.11	0.39
8	0.01	7.63	4.15	1.85	7.70	6.54	15.64	0.10
9	0.35	12.90	3.56	1.65	3.56	19.07	21.48	0.29
10	0.08	31.77	6.70	1.75	5.92	33.56	17.95	0.15
11	0.55	59.80	8.92	1.83	3.28	63.40	28.99	0.21
12	0.24	78.71	8.78	1.43	2.97	43.42	20.63	0.45
13	20.82	0.30	0.90	0.92	1.14	0.53	0.88	1.02
14	5.78	4.51	2.13	1.12	1.86	2.63	3.34	0.64
15	60.71	0.07	0.45	0.80	0.50	0.22	0.30	1.17

* fold enrichment
