# Supplementary material for: Altered Chromatin Occupancy of Master Regulators Underlies Evolutionary Divergence in the Transcriptional Landscape of Erythroid Differentiation
Source: PLoS Genet. 2014 Dec 18;10(12):e1004890. doi: 10.1371/journal.pgen.1004890 (PMC4270484; doi:10.1371/journal.pgen.1004890)
Supplement: S6 Table — Predictive promoter models of transcription. Both the species-specific and consensus models applied to promoter marks are applied to gene expression across terminal erythroid differentiation. Abbreviations used: none. (PDF) [file pgen.1004890.s021.pdf]

Model learned on each species independently

mouse	R^2	human	R^2
ProE	0.62	ProE	0.56
BasoE	0.57	eBasoE	0.56
PolyE	0.56	lBasoE	0.55
OrthE	0.54	PolyE	0.47
		OrthE	0.39

Model learned on both species together

mouse	R^2	human	R^2
ProE	0.61	ProE	0.57
BasoE	0.57	eBasoE	0.56
PolyE	0.56	lBasoE	0.55
OrthE	0.53	PolyE	0.47
		OrthE	0.40
