# Supplementary material for: Altered Chromatin Occupancy of Master Regulators Underlies Evolutionary Divergence in the Transcriptional Landscape of Erythroid Differentiation
Source: PLoS Genet. 2014 Dec 18;10(12):e1004890. doi: 10.1371/journal.pgen.1004890 (PMC4270484; doi:10.1371/journal.pgen.1004890)
Supplement: S4 Dataset — Mouse proerythroblast GATA1 motif discovery results. Raw motif results. Abbreviations used: none. (ZIP) [file pgen.1004890.s025.zip › Dataset S4 mouse_GATA1.tar!/appMEMECHIP_4.9.11402016772261-554169689/meme_out/meme.html]

MEME


For further information on how to interpret these results or to get a
copy of the MEME software please access
http://meme.nbcr.net.

If you use MEME in your research, please cite the following paper:  
Timothy L. Bailey and Charles Elkan,
"Fitting a mixture model by expectation maximization to discover motifs in biopolymers",
*Proceedings of the Second International Conference on Intelligent Systems
for Molecular Biology*, pp. 28-36, AAAI Press, Menlo Park, California, 1994.

Discovered Motifs
  |  
Block diagrams of Motifs
  |  
Program information
  |  
Explanation

## Discovered Motifs

#### Motif Overview

|  |  |  |  |  |
| --- | --- | --- | --- | --- |
| Motif 1 | - 2.8e-190 - 317 sites |  |  |  |
| Motif 2 | - 6.6e-182 - 71 sites |  |  |  |
| Motif 3 | - 4.7e-160 - 51 sites |  |  |  |

#### Further Analysis

Submit all motifs to
 


    
 


    
 


    
 


 Mouse-over buttons for more information.

|  |  |
| --- | --- |
| Motif 1 | Next Top |

| Summary | Sequence Logo |
| --- | --- |
| E-value \| 2.8e-190; Width \| 10; Sites \| 317 show more Log Likelihood Ratio \|; \| 2579; Information Content \|; \| 11.6 (bits); Relative Entropy \|; \| 11.7 (bits) show less | StandardReverse Complement   Download LOGO      Orientation: standard reverse complement    SSC: off on    Format: web (png) publication (eps)    Width:  cm    Height:  cm |

#### Regular expression

[GAT][CG][AT]GATAA[GC][AGC]

#### Further Analysis

Submit this motif to
 


    
 


    
 


    
 


    
 


 Mouse-over buttons for more information.

#### Data Formats

View the motif in
PSPM Format 


    
PSSM Format 


    
BLOCKS Format 


    
FASTA Format 


    
Raw Format 


    
or
Hide  
This feature requires javascript.

#### Sites

Click on any row to highlight sequence in all motifs.

| Name | Strand | Start | *p*-value | Sites | | |
| --- | --- | --- | --- | --- | --- | --- |
| chr11:69589369-69589469 | - | 48 | 1.75e-06 | CCGAGAAGCC | ACAGATAAGA | GACGTCCCTA |
| chr4:136653119-136653219 | - | 31 | 1.75e-06 | TGTGTGTGCT | GCAGATAAGA | CTGTTAGAAG |
| chr15:78268012-78268112 | + | 76 | 1.75e-06 | GCTGGTTGGG | ACAGATAAGA | GCAGAGCAGG |
| chr4:41026584-41026684 | + | 64 | 1.75e-06 | AGATGACTCA | GCAGATAAGA | GCACTGGCTG |
| chr3:96082800-96082900 | - | 33 | 1.75e-06 | CTGGTGGTTG | GCAGATAAGA | CTCTGGCAGA |
| chr19:55865965-55866065 | + | 33 | 1.75e-06 | ATAACTGCCT | GCAGATAAGA | GGGGCTTCCT |
| chr9:66347548-66347648 | - | 66 | 1.75e-06 | TCAGGAGTCT | ACAGATAAGA | GTCCTGCTGT |
| chr12:40036729-40036829 | - | 56 | 1.75e-06 | GAAATTGTAA | GCAGATAAGA | AGAACGCGTT |
| chr2:181458507-181458607 | - | 52 | 1.75e-06 | AGTGGGCAGC | GCAGATAAGA | CGCAGCGCTC |
| chr11:105366112-105366212 | - | 33 | 3.49e-06 | TGTGGCATAA | AGAGATAAGA | GGCCCAGCAG |
| chr11:79008560-79008660 | - | 23 | 3.49e-06 | TCCTCCCAAC | AGAGATAAGA | GCCTCAGGGA |
| chr10:70133348-70133448 | - | 62 | 3.49e-06 | TGAGGTGTGA | AGAGATAAGA | AGGTGTTGCA |
| chr11:68884524-68884624 | - | 29 | 3.49e-06 | TTGGCTCCTT | GGAGATAAGA | CCTGCCTTAG |
| chr14:21549388-21549488 | + | 82 | 3.49e-06 | GCAGAAGGAC | AGAGATAAGA | CTTTCAGAT |
| chr15:53202459-53202559 | - | 13 | 3.49e-06 | GTGTGGTCAG | GGAGATAAGA | GATAGGTAGG |
| chr13:31909533-31909633 | + | 79 | 3.49e-06 | AGATGGCTCA | GGAGATAAGA | GCATTGGCTC |
| chr6:122394334-122394434 | + | 69 | 3.49e-06 | TGCTGGAGAC | AGAGATAAGA | CAGAAGCAAC |
| chr2:19024069-19024169 | - | 51 | 3.49e-06 | GATTTTGCCT | GGAGATAAGA | TTCTTCAGGG |
| chr8:60900206-60900306 | + | 17 | 3.49e-06 | GATGAGACAG | AGAGATAAGA | AGGTAGGGTC |
| chr14:70107111-70107211 | - | 32 | 3.49e-06 | CTGCAGGGAC | AGAGATAAGA | GGCTGACCCA |
| chr3:60546330-60546430 | + | 36 | 3.49e-06 | CCCGGCTGTG | GGAGATAAGA | GCCATGAGGA |
| chr10:127126665-127126765 | - | 50 | 4.34e-06 | CAGGAACGGG | TCAGATAAGA | AAGTGGCCAG |
| chr1:3435154-3435254 | + | 61 | 4.34e-06 | ATGGGCTAGA | TCAGATAAGA | CCAAGAAGCC |
| chr8:71960207-71960307 | - | 64 | 4.34e-06 | AGCTGGCACA | TCAGATAAGA | GCATTTACAG |
| chrX:7837292-7837392 | + | 67 | 7.04e-06 | CTGGACCTGC | TGAGATAAGA | GACTAACATC |
| chr6:134984894-134984994 | + | 30 | 7.04e-06 | TGTGAAGCCC | TGAGATAAGA | AAGGACCTTC |
| chr4:33450487-33450587 | + | 51 | 7.04e-06 | ACTGGGGACA | GCAGATAAGG | CAAGCCACAT |
| chr2:28323025-28323125 | - | 36 | 7.04e-06 | TGGAGATTGG | GCAGATAAGG | CCCTGGGTCT |
| chr11:32269108-32269208 | - | 24 | 7.04e-06 | AAGTGACGGG | ACAGATAAGG | CCTGGCACCC |
| chr13:95965321-95965421 | + | 12 | 1.39e-05 | CATGTGTGTC | GCTGATAAGA | AAAAGGAAAA |
| chr7:132492076-132492176 | - | 57 | 1.39e-05 | AAAATGTCCC | ACAGATAAGC | CCACATGCCT |
| chr17:46679988-46680088 | + | 56 | 1.39e-05 | GAAAATGCAC | AAAGATAAGA | CTACAATCCC |
| chr13:43191907-43192007 | - | 15 | 1.39e-05 | GGAAGGCTAT | GCAGATAAGC | GAACTCACAT |
| chr19:53277633-53277733 | + | 53 | 1.39e-05 | TGATGGCAGT | GTAGATAAGA | GAGCCGAGTG |
| chr6:29583606-29583706 | + | 85 | 1.39e-05 | AGATGGCTCA | GTAGATAAGA | GCACTG |
| chr7:102958803-102958903 | - | 4 | 1.39e-05 | TCTGCTATGT | GTAGATAAGA | GCC |
| chr7:122015208-122015308 | + | 22 | 1.39e-05 | ACACTGCTGC | ACAGATAAGC | ACTTAAAGAC |
| chr9:99242846-99242946 | - | 44 | 1.39e-05 | CCTGTTAGAC | ACTGATAAGA | CTGGTTAATA |
| chr5:35035873-35035973 | - | 40 | 1.39e-05 | GCTGGGGTGG | GCAGATAAGC | CTTACAGACA |
| chr7:97589986-97590086 | + | 46 | 1.39e-05 | AGCAAAGGCA | ATAGATAAGA | TCTTGCTGCT |
| chr4:103120154-103120254 | + | 41 | 1.39e-05 | TCTTGTCAAG | GCAGATAAGC | AAGCGCTCTA |
| chr9:72902765-72902865 | - | 6 | 1.39e-05 | TGCTGCCAAG | GCTGATAAGA | TAAGC |
| chr10:108582493-108582593 | + | 84 | 1.39e-05 | AGTGCTTGGG | GTAGATAAGA | GTGCTAC |
| chr2:151011403-151011503 | - | 62 | 1.39e-05 | GCTGCTGAGA | ATAGATAAGA | TACTGGCTTG |
| chr11:23313687-23313787 | - | 25 | 1.39e-05 | CCACTGCTCA | ACAGATAAGC | CACGTGCTGC |
| chr7:115831009-115831109 | + | 49 | 1.39e-05 | AGCACAGGCT | GTAGATAAGA | AAAGTGCTGC |
| chr19:9899580-9899680 | - | 3 | 1.39e-05 | CTAACAGCCA | GCAGATAAGC | GG |
| chr5:118489137-118489237 | + | 69 | 1.39e-05 | TGGTGGTGAA | GCTGATAAGA | AGCTGGGTGA |
| chr13:97066815-97066915 | + | 78 | 1.39e-05 | TGTGCAAAGG | GAAGATAAGA | GGGAGGGTAC |
| chr11:48838510-48838610 | + | 41 | 1.39e-05 | GAGGAGAGCT | GAAGATAAGA | TAGACCGAAC |
| chr9:3328343-3328443 | + | 57 | 1.39e-05 | CCTTGTTCAC | ACAGATAAGC | ACTGCAGAAG |
| chr7:44483007-44483107 | - | 39 | 1.39e-05 | TTGGGTCAGA | ATAGATAAGA | GGGGAGGAGA |
| chr13:51976267-51976367 | - | 55 | 1.39e-05 | GCTGGAGTGT | GCTGATAAGA | GTCTTACCAG |
| chr14:21823238-21823338 | - | 5 | 1.39e-05 | ACTGGAGAAG | ACTGATAAGA | AAGA |
| chr18:38350130-38350230 | - | 19 | 1.39e-05 | GTAGCCTTGA | GCAGATAAGC | TAGCGCAGCT |
| chr13:8871701-8871801 | - | 53 | 1.39e-05 | GTCATCTTCT | GCAGATAAGC | TGAAATATTA |
| chr2:181055406-181055506 | - | 27 | 1.39e-05 | GATCCCCTGA | GCAGATAAGC | TGAATTCTAT |
| chr12:105019665-105019765 | - | 36 | 1.67e-05 | CGGAGCCTGT | GGAGATAAGG | CATCTGGCCC |
| chr5:137116487-137116587 | + | 85 | 1.67e-05 | GCTGTGAGCC | CCAGATAAGA | ACATTT |
| chr5:24927981-24928081 | + | 52 | 1.67e-05 | GACGCTGCGG | CCAGATAAGA | GAGGAGGAGA |
| chr6:53134583-53134683 | + | 51 | 1.67e-05 | AGATCGAGCA | AGAGATAAGG | GAAAATGAAG |
| chr18:67246200-67246300 | + | 46 | 1.67e-05 | TGCAATGCGT | GGAGATAAGG | GCGTGGGATG |
| chr18:82568460-82568560 | - | 32 | 1.67e-05 | GCCCAGTGCT | GGAGATAAGG | CAGCGCGAAC |
| chr14:52306274-52306374 | - | 49 | 1.67e-05 | GTCATGGGCT | GGAGATAAGG | CTCAGTGGTA |
| chr16:17220559-17220659 | + | 37 | 1.67e-05 | CAAGGTCAGG | CCAGATAAGA | GCTGCAATAG |
| chr2:152741639-152741739 | - | 51 | 1.67e-05 | GTGGGGTCAG | AGAGATAAGG | GAGCCGGGCC |
| chr9:75547297-75547397 | - | 7 | 1.67e-05 | AGCAAGGACT | GGAGATAAGG | ATGTTG |
| chr16:32541427-32541527 | + | 75 | 1.67e-05 | GGCTGGAGGC | AGAGATAAGG | TTGTGAACCA |
| chr15:27497059-27497159 | - | 70 | 1.67e-05 | CCTGTGGGAC | CCAGATAAGA | ACCGCTGTCC |
| chr6:86364837-86364937 | - | 5 | 1.67e-05 | GTGGTCCCAT | AGAGATAAGG | GAAA |
| chr6:123293105-123293205 | - | 79 | 1.67e-05 | TCTGCACCAG | AGAGATAAGG | CTGTTCCACA |
| chr17:71681163-71681263 | - | 3 | 1.67e-05 | GATCTTTGAG | GGAGATAAGG | GG |
| chr17:5099381-5099481 | + | 88 | 1.67e-05 | CTGGGCTGTG | AGAGATAAGG | CTT |
| chr6:32534120-32534220 | + | 8 | 1.67e-05 | GGAACAG | GGAGATAAGG | TGGCCTTCTG |
| chr11:73047749-73047849 | + | 15 | 1.67e-05 | AGGGGGCGCC | AGAGATAAGG | ACAGCTTTCT |
| chr8:122296354-122296454 | - | 73 | 2.29e-05 | TCTCCAGATG | GGTGATAAGA | ACTGAGCCAT |
| chr10:94073013-94073113 | - | 7 | 2.29e-05 | GGCTGTCTCA | GCAGATAACA | AACTGC |
| chr2:118660266-118660366 | - | 54 | 2.29e-05 | CCCAGCAGCC | ACAGATAACA | GAGGGTGCAG |
| chr19:40977065-40977165 | - | 50 | 2.29e-05 | TATAGCAGCC | AGTGATAAGA | GAAATTACTC |
| chr2:60223668-60223768 | - | 53 | 2.29e-05 | AGATGGCTCA | TCAGATAAGG | CAGCTGGTGC |
| chr12:71164592-71164692 | - | 36 | 2.29e-05 | AGTGCTGCGC | ACAGATAACA | AACATAAAGC |
| chr10:77210625-77210725 | - | 18 | 2.29e-05 | AGCCAAGATC | ACAGATAACA | GTCCTAGAAA |
| chr14:76520693-76520793 | + | 69 | 2.29e-05 | CCCAGAGTAA | ACAGATAACA | GGCAAGAAGG |
| chr7:128672450-128672550 | - | 72 | 2.29e-05 | GGAGAAGGAC | ACAGATAACA | CAGATAAATG |
| chr8:85526315-85526415 | + | 4 | 2.29e-05 | AGC | AGAGATAAGC | AAGGGCTGAA |
| chr9:44343366-44343466 | + | 24 | 2.29e-05 | TGCCTGGGCA | GGAGATAAGC | AGGCTTCCCA |
| chr15:97844710-97844810 | + | 57 | 2.29e-05 | ATAATGGCAT | GCAGATAACA | GTGCCCAGGG |
| chr11:120393791-120393891 | - | 33 | 2.29e-05 | AGTAGATGCT | GCAGATAACA | CCAAACTTTC |
| chr7:64058092-64058192 | + | 58 | 2.29e-05 | TTCTGCTCTT | GGTGATAAGA | CCTAGCACAA |
| chr19:6284558-6284658 | + | 52 | 2.72e-05 | GAACACAGCC | TCAGATAAGC | GACAATAGTT |
| chr15:82342048-82342148 | + | 36 | 2.72e-05 | AGCTGTGTGG | TTAGATAAGA | GGCCACCCTG |
| chr18:32556803-32556903 | - | 88 | 2.72e-05 | GGC | TCTGATAAGA | CATGCTATTA |
| chr2:93443949-93444049 | - | 70 | 2.72e-05 | AGTAAAGAGA | TCAGATAAGC | ACGTGGGAAA |
| chr16:21357574-21357674 | + | 31 | 2.72e-05 | TCACCTCTGT | TCTGATAAGA | TTGGCAAACG |
| chr3:75918202-75918302 | - | 58 | 2.98e-05 | TGTGCTCCAG | TGAGATAAGG | GGCGCTACTT |
| chr4:124645091-124645191 | - | 68 | 2.98e-05 | GGGGAGCTGG | TGAGATAAGG | AGGGAAGGGC |
| chr4:134663256-134663356 | + | 35 | 2.98e-05 | TGTCTAACCT | TGAGATAAGG | TTCCCCACAC |
| chr11:60486424-60486524 | + | 41 | 2.98e-05 | CAGGAAGCCA | TGAGATAAGG | CCTGGATCAG |
| chr10:93679867-93679967 | + | 78 | 2.98e-05 | GCCTGCGTGA | AGAGATAACA | GAAATGCTGG |
| chr18:44335469-44335569 | + | 49 | 2.98e-05 | GATCAGAGGC | AGAGATAACA | CCAAAGGTGA |
| chr14:47116162-47116262 | + | 15 | 2.98e-05 | GCAAGGGGAA | GGAGATAACA | CACTTGGCCT |
| chr3:88325269-88325369 | + | 33 | 2.98e-05 | TGGATGCAAG | AGAGATAACA | ACCCAGAGAG |
| chr4:89510153-89510253 | - | 58 | 2.98e-05 | CACTCAGCTC | AGAGATAACA | GGCAGCAAAG |
| chr13:97453145-97453245 | - | 66 | 2.98e-05 | CCTGGCTCTG | TGAGATAAGG | CCTAACAGCA |
| chr12:8609275-8609375 | + | 55 | 3.78e-05 | TGTGCCTCCC | TCAGATAACA | TGTTCTACCG |
| chr2:154634391-154634491 | - | 38 | 3.78e-05 | TCCTGTTATG | TCAGATAACA | AAGTCCCTAA |
| chr1:75450526-75450626 | + | 32 | 3.78e-05 | CTGTGCGGGT | GCTGATAAGG | GAAGCCACAA |
| chr1:136014964-136015064 | - | 85 | 3.78e-05 | GCAGAA | TGTGATAAGA | ATATGCGAAC |
| chr9:76236716-76236816 | + | 47 | 3.78e-05 | TAAACACTCA | TCAGATAACA | GGACTGCCTG |
| chr13:101573340-101573440 | + | 68 | 3.78e-05 | GCTGGGGCTG | TCAGATAACA | ACGGAATGTG |
| chr4:126803606-126803706 | + | 84 | 3.78e-05 | CCTCTTATCA | GAAGATAAGG | CATGGGG |
| chr7:115988546-115988646 | + | 36 | 3.78e-05 | CCTGCAGGGA | ACTGATAAGG | TAGTCCTATA |
| chr11:75234827-75234927 | + | 77 | 3.78e-05 | GGGTGCGCGT | GCTGATAAGG | GTTGCAGGAT |
| chr17:30347302-30347402 | + | 71 | 3.78e-05 | TGCTGCCGCC | TCAGATAACA | GCTGCTGCAG |
| chr15:79741596-79741696 | - | 33 | 3.78e-05 | TATGAAAGAT | AAAGATAAGG | TCCACACCGC |
| chr2:132695865-132695965 | + | 31 | 3.78e-05 | GCCTGCCCTG | GTAGATAAGG | TGAAGACTCC |
| chr7:19632888-19632988 | + | 20 | 3.78e-05 | TGTGTGGAGG | TCAGATAACA | CTATAGTTGC |
| chr4:154541753-154541853 | - | 20 | 3.78e-05 | AGGTGCTCGG | TGAGATAAGC | CGATCTGCCA |
| chr5:73354169-73354269 | - | 27 | 3.78e-05 | TCTCTGGAAA | TGTGATAAGA | TAAGTGGAAT |
| chr12:84161266-84161366 | - | 70 | 3.78e-05 | AGCCTCCTGG | ATAGATAAGG | GAAGAATTTG |
| chr6:143833202-143833302 | - | 57 | 3.78e-05 | GGACAAGCCA | ACTGATAAGG | GCCAACAGAC |
| chr17:24527396-24527496 | - | 66 | 4.90e-05 | CGGATTCCAA | ACTGATAAGC | TCATCTGGTA |
| chr6:38757779-38757879 | - | 60 | 4.90e-05 | AATCACATGA | CCAGATAAGG | GTGGGAGAAA |
| chr18:32024987-32025087 | + | 87 | 4.90e-05 | AGATTTTCCA | ACTGATAAGC | AAAG |
| chr1:9782189-9782289 | - | 39 | 4.90e-05 | GATGGACGAG | GAAGATAAGC | TTTCAAGCAG |
| chr7:110920405-110920505 | - | 57 | 4.90e-05 | GCTGCCTGGC | ACTGATAAGC | CCTGAGTAAA |
| chr10:77232495-77232595 | + | 52 | 4.90e-05 | TGTAAAGCGA | GCTGATAAGC | AGCTCTCCTC |
| chr5:120599783-120599883 | + | 48 | 4.90e-05 | CACGAAGCCA | AAAGATAAGC | TTGCCAGGGT |
| chr11:52115665-52115765 | + | 22 | 4.90e-05 | CTGGCAGCTA | ACAGATAAAA | TGTAGCCATA |
| chr7:125472198-125472298 | - | 11 | 4.90e-05 | AAATGCGACT | GTAGATAAGC | TGGAGGACTT |
| chr15:37100038-37100138 | + | 43 | 4.90e-05 | ACAGAAGGCA | ACTGATAAGC | ATGTGACTTG |
| chr5:50126334-50126434 | - | 68 | 4.90e-05 | GAGAGTCTGT | ATTGATAAGA | TAATGAACCC |
| chr17:24628796-24628896 | + | 27 | 4.90e-05 | CCAGAAGGGC | ACTGATAAGC | TCCCAAGGCA |
| chr2:28418164-28418264 | + | 83 | 4.90e-05 | ACTAACCATT | ATAGATAAGC | TCACAGCA |
| chr9:103288322-103288422 | - | 51 | 4.90e-05 | ATCTGCTTGG | CCAGATAAGG | AAGGCATGGA |
| chr14:73322227-73322327 | - | 85 | 4.90e-05 | CACAAA | AAAGATAAGC | ATATGTTTAA |
| chr19:4878888-4878988 | - | 35 | 4.90e-05 | AGATTGAGCA | GCTGATAAGC | AGCTAGCGCC |
| chr9:21384160-21384260 | - | 54 | 4.90e-05 | AGATGTGTAG | GATGATAAGA | GGTCAGAGAC |
| chr8:14890864-14890964 | - | 74 | 4.90e-05 | GCCGTGTGTG | GTTGATAAGA | GTTAGATGAG |
| chr17:29658328-29658428 | - | 53 | 5.71e-05 | CAAGGCTGTC | CCAGATAAGC | AGAACCCAGT |
| chr14:63067779-63067879 | - | 42 | 5.71e-05 | TGAACTTAGT | GGTGATAAGG | GCCAGATAAT |
| chr11:117308077-117308177 | - | 31 | 5.71e-05 | CCCCCAGTCA | CCAGATAAGC | ATTAGTCAGC |
| chr18:10092913-10093013 | + | 55 | 5.71e-05 | AGTAGGTGTG | GGTGATAAGG | GCAGATCATG |
| chr11:109557500-109557600 | + | 25 | 5.71e-05 | ACGTGCTATT | CCTGATAAGA | CCGTGGCCAG |
| chr4:42880272-42880372 | + | 64 | 5.71e-05 | AGCTGCAAGT | CCTGATAAGA | AGGCCTTGCT |
| chr6:125104401-125104501 | - | 29 | 5.71e-05 | GTCTGTTCAG | AGTGATAAGG | TAGGTATCAG |
| chr1:167273957-167274057 | + | 41 | 5.71e-05 | CAATTAGGAC | TGAGATAACA | GTTATCTCCT |
| chr12:80930283-80930383 | + | 71 | 7.28e-05 | CGTGACTCAG | GCAGATAAGT | AGGCTCACGT |
| chr9:45803597-45803697 | + | 23 | 7.28e-05 | GCGGGGAGGC | AGAGATAAAA | CCCCAGCTAC |
| chr9:96254978-96255078 | - | 42 | 7.28e-05 | CCAGCAAGTT | TTAGATAAGG | GCCTAATAAG |
| chr12:12427396-12427496 | + | 21 | 7.28e-05 | ATGTGCATAC | TCTGATAAGG | ATGCCCACAT |
| chr4:134924511-134924611 | - | 37 | 7.28e-05 | GAGGTGGCTC | GGTGATAAGC | GTCCAGAACT |
| chr2:174297079-174297179 | + | 52 | 7.28e-05 | TGTGGGGTCC | TTAGATAAGG | AATTTACCGC |
| chr9:57070405-57070505 | - | 41 | 7.28e-05 | CTGGCTCAGT | GTAGATAACA | GAAGAGGTAG |
| chr6:116350164-116350264 | - | 63 | 7.28e-05 | GCAACAGTGA | GAAGATAACA | ACTATCAATA |
| chr12:32143280-32143380 | - | 84 | 7.28e-05 | ACCCTGG | AGTGATAAGC | ACACAGGGTT |
| chr5:31733120-31733220 | - | 39 | 7.28e-05 | GATCGTCCTA | GCTGATAACA | AAGCTGAGTC |
| chr17:47598269-47598369 | - | 9 | 7.28e-05 | GACTGGAGCC | AGAGATAAAA | GGCCAGAG |
| chr16:49771816-49771916 | + | 21 | 7.28e-05 | CGAACATTTG | ACAGATAACC | ACTTGTTTTG |
| chr16:34031700-34031800 | + | 91 | 7.28e-05 | TGTGGGGTTT | TTAGATAAGG |  |
| chr10:81352088-81352188 | + | 59 | 7.28e-05 | ATAGAGCTTC | TTAGATAAGG | TTAGGCTTGA |
| chr16:91865025-91865125 | + | 76 | 7.28e-05 | GGTGTGAGAG | ACAGATAAGT | CTAAGACAGA |
| chr4:144981198-144981298 | + | 43 | 7.28e-05 | CTGAGTACAG | GCTGATAACA | GAGGACACTC |
| chr2:170128845-170128945 | - | 49 | 7.28e-05 | CTGTCTCCTG | GCAGATAAGT | GCTAATCACT |
| chr7:142087231-142087331 | + | 30 | 7.28e-05 | TATGCATGTG | ACTGATAACA | GAGGCTCAGG |
| chr9:67013624-67013724 | - | 38 | 7.28e-05 | ATGCTCCTAA | AAAGATAACA | CAGGCTGTAA |
| chr16:58497980-58498080 | + | 53 | 7.28e-05 | TTCTGGAAGT | GCTGATAACA | GGAAAGAGGA |
| chr18:82634085-82634185 | + | 26 | 8.24e-05 | AGAAGCCTGA | TAAGATAAGC | CTCAAGCCAG |
| chr17:83489251-83489351 | - | 11 | 8.24e-05 | GATGGCAGCC | TTAGATAAGC | AATCCCAGGG |
| chr9:64779736-64779836 | + | 83 | 8.24e-05 | CACATTTGCC | TCAGATAAAA | ATCTTAAC |
| chr6:120174624-120174724 | + | 41 | 8.24e-05 | AGCTGCAGGT | TAAGATAAGC | TGAGCTGAGA |
| chr2:11212331-11212431 | - | 18 | 8.24e-05 | GATCCCCAGG | TTAGATAAGC | ACCAGTAATT |
| chr5:130029677-130029777 | + | 16 | 8.24e-05 | AGTGCGACCC | AGAGATAACG | TCCCAGTCAT |
| chr2:153525950-153526050 | - | 10 | 8.24e-05 | AAGATCTTTC | TATGATAAGA | GCTAGCCAG |
| chr10:59875541-59875641 | + | 43 | 8.24e-05 | GCTGTGGTGG | TTTGATAAGA | AATGGCCCCA |
| chr5:143819088-143819188 | + | 56 | 8.24e-05 | TCTGTGACGC | CGTGATAAGA | GTGTCTGCAC |
| chr11:96075674-96075774 | - | 28 | 8.24e-05 | TGGAACGTGG | AGAGATAACG | ACCAATGGGA |
| chr13:24681926-24682026 | - | 66 | 8.24e-05 | GTGAGATGGG | TATGATAAGA | CAGGTATGGT |
| chr11:116615802-116615902 | - | 7 | 8.95e-05 | TCTGGGGGCG | GGAGATAAGT | GGCGGG |
| chr17:84926353-84926453 | - | 78 | 8.95e-05 | GTTCTCAGTG | GGTGATAACA | GCAGGCTACC |
| chr11:95835267-95835367 | + | 17 | 8.95e-05 | TTGGGCACAG | TGTGATAAGG | TCCCCTCCGC |
| chr7:16412641-16412741 | - | 12 | 8.95e-05 | CTACAGACCA | AGTGATAACA | CAACTAGCCC |
| chr5:37456736-37456836 | - | 16 | 8.95e-05 | AGACATACTG | GGTGATAACA | CTTAGCTGGA |
| chr1:87712410-87712510 | - | 51 | 8.95e-05 | TCTGCCAAGT | AGAGATAACC | ATACTTGACT |
| chr3:130810201-130810301 | + | 34 | 8.95e-05 | GATGGAGGAA | GGAGATAAGT | ACCTGCTGCT |
| chr9:115518039-115518139 | + | 20 | 8.95e-05 | CTTCCCACTC | AGTGATAACA | GCAATGTGTG |
| chr12:101084027-101084127 | - | 83 | 8.95e-05 | AGAGAAAG | GGAGATAACC | GCTGATTATT |
| chr6:119361106-119361206 | - | 15 | 1.02e-04 | ACTGTGCTGA | ACAGATAAAG | TTCTGTATCA |
| chr16:90144449-90144549 | + | 51 | 1.02e-04 | TTTGCTTAGG | TGAGATAAAA | TCTCAACGCG |
| chr16:76370166-76370266 | + | 35 | 1.02e-04 | CCTTGGACAA | TGTGATAAGC | CACATGGCAG |
| chr6:28710210-28710310 | - | 12 | 1.02e-04 | TCCACTCCTA | TCAGATAACC | CAGCCTGTAG |
| chr7:27454859-27454959 | - | 58 | 1.02e-04 | AGTTCTACCG | TTAGATAACA | GACTGAAGGG |
| chr17:79926012-79926112 | + | 21 | 1.02e-04 | TGATGACAAT | GTTGATAAGG | AAAACCGGTC |
| chr6:38831161-38831261 | + | 44 | 1.02e-04 | GATTTCTCCC | GTTGATAAGG | ACCTCTGTCA |
| chr3:79580337-79580437 | + | 12 | 1.02e-04 | CCCTGGGTGG | AATGATAAGG | ACTCAGCTTT |
| chr14:65145074-65145174 | + | 15 | 1.15e-04 | GCAGGCGATG | CTAGATAAGG | CCCTTCTGTT |
| chr12:111518050-111518150 | + | 2 | 1.15e-04 | G | ATAGATAAAA | TGTTTATTCA |
| chr6:5287482-5287582 | + | 84 | 1.15e-04 | TCTGTCACCC | ATTGATAAGC | TAACTGT |
| chr2:120969738-120969838 | + | 5 | 1.15e-04 | ACAA | ACTGATAAAA | CATGTTGTGA |
| chr19:56543498-56543598 | + | 77 | 1.15e-04 | GAAGGCATGG | GCAGATAAAC | AAAAGCAGAA |
| chr15:38301922-38302022 | - | 66 | 1.15e-04 | GTCCCAGGGA | GAAGATAAAA | ATCTCTAGTT |
| chr11:69258426-69258526 | + | 63 | 1.15e-04 | ATCACCCAAG | CAAGATAAGG | ACTCCCTTCC |
| chr1:159238253-159238353 | + | 35 | 1.15e-04 | AAATGGCACA | GTAGATAAAA | CAGTTGCTGT |
| chr11:85298177-85298277 | - | 45 | 1.15e-04 | GGTTGAGAAC | CAAGATAAGG | AAAACACCCC |
| chr14:79530302-79530402 | + | 25 | 1.15e-04 | CTGGTGCTCT | GCTGATAAAA | ACATGAGTGA |
| chr8:122330606-122330706 | - | 87 | 1.15e-04 | CCAA | CAAGATAAGG | CCTACTAGCT |
| chr2:166665721-166665821 | - | 19 | 1.15e-04 | TGGAAGGAAC | CCTGATAAGG | CCATTGGTTC |
| chr3:122113598-122113698 | - | 79 | 1.15e-04 | GAAAGATCAC | CAAGATAAGG | TGACATTTGA |
| chr16:90197429-90197529 | - | 76 | 1.15e-04 | TGCTCCTCCA | GATGATAAGC | CTTGGCTTCC |
| chrX:159597637-159597737 | - | 37 | 1.15e-04 | ATGTAGCTCA | ATAGATAAAA | CATCTGCCTT |
| chr5:145152785-145152885 | + | 84 | 1.15e-04 | GGAAAAAAAA | AAAGATAAAA | AAAACCC |
| chr11:60914061-60914161 | + | 21 | 1.30e-04 | GCCTGAGGCA | ATAGATAACG | TCAAATGCAA |
| chr12:83073377-83073477 | + | 30 | 1.30e-04 | GGTGTTTGCT | GGAGATAAAG | CGGCCCAGGC |
| chr6:38921194-38921294 | + | 32 | 1.30e-04 | CCGTGTCTTG | CCTGATAAGC | TGCATAGCCG |
| chr17:79365207-79365307 | + | 79 | 1.30e-04 | CAGGCCAGGG | CTAGATAAGC | CCCTTGACTC |
| chr2:119163310-119163410 | - | 19 | 1.30e-04 | CCTGCAGCCT | CCTGATAAGC | ATCTCGAATC |
| chr9:59271788-59271888 | - | 9 | 1.30e-04 | GCGAGGGAGT | CCTGATAAGC | AAGCTGCT |
| chr11:61067579-61067679 | + | 72 | 1.30e-04 | CCTGTGATGG | GAAGATAACG | CATGTTCAGA |
| chr13:57659949-57660049 | + | 7 | 1.30e-04 | GGCCCC | TGTGATAACA | TCAATCTCTC |
| chr18:35619702-35619802 | + | 87 | 1.30e-04 | GTTTTGCCTG | CTAGATAAGC | ACTC |
| chr12:100958677-100958777 | - | 74 | 1.30e-04 | ACCCCTGAAT | GGAGATAAAG | CGCTCTGGAA |
| chr9:122104015-122104115 | + | 25 | 1.30e-04 | CACTGAGAGC | CAAGATAAGC | CCTGACTCTC |
| chr6:120530699-120530799 | + | 84 | 1.53e-04 | TGATGATGAT | GATGATAACA | GTTCCTT |
| chr4:41129997-41130097 | - | 18 | 1.53e-04 | CCAGAGCCAA | GAAGATAACC | AGTATCACAA |
| chr2:28032679-28032779 | + | 53 | 1.53e-04 | CACAGGCAGC | TATGATAAGG | CTATCCGCTT |
| chr19:7552151-7552251 | - | 9 | 1.53e-04 | TGTGTTGCAC | AGTGATAAAA | GCCCTGGG |
| chr4:132530565-132530665 | - | 10 | 1.53e-04 | TGTGCTACTA | ACAGATAATA | AACGATCAC |
| chr11:90664423-90664523 | - | 37 | 1.53e-04 | TGTGCTGTGG | GCAGATAATA | GGGGCATTGC |
| chr15:80130822-80130922 | + | 42 | 1.53e-04 | TCTGGACACT | CGTGATAAGG | CCCTGAGCCA |
| chr12:71904407-71904507 | + | 15 | 1.53e-04 | CTTATGCCAG | TATGATAAGG | GTTACGTCAC |
| chr13:73443549-73443649 | - | 66 | 1.53e-04 | CTGCATTCTC | TATGATAAGG | GTAAGGGAGA |
| chr8:94964944-94965044 | - | 5 | 1.53e-04 | AGTGCAGACG | GTTGATAACA | TTTT |
| chr2:172298240-172298340 | - | 37 | 1.53e-04 | CCTGCAGAGA | ATAGATAACC | TAGCCATGCT |
| chr4:119198681-119198781 | - | 57 | 1.53e-04 | TACGATAAGC | ATAGATAAGT | CACTTCAACT |
| chr10:40328063-40328163 | - | 33 | 1.66e-04 | TAAGTTATGA | TAAGATAAAA | AGCACACACA |
| chr3:55080176-55080276 | + | 43 | 1.66e-04 | GATCTGGGGA | CCTGATAACA | GGAAGACTAA |
| chr9:66895075-66895175 | + | 4 | 1.66e-04 | CTC | TTTGATAAGC | TGTTTGATTA |
| chr4:46075811-46075911 | - | 36 | 1.66e-04 | GATCTTGCAG | CCAGATAACC | CTGGCTTAGC |
| chr17:47210549-47210649 | - | 83 | 1.66e-04 | TCAAAGGG | TATGATAAGC | CCAAGTTAGT |
| chr11:11835494-11835594 | + | 51 | 1.66e-04 | ACAATACACT | CGAGATAAAA | GTTTACAGGC |
| chr3:51387110-51387210 | - | 34 | 1.66e-04 | GGGAGATGGC | CTAGATAACA | CAGAGTTATG |
| chr11:86758157-86758257 | + | 71 | 1.66e-04 | CTCGAGATCC | TCAGATAAAC | CATCTCAAAG |
| chr1:157695418-157695518 | + | 83 | 1.78e-04 | TGTGGGTTCA | TGAGATAAAG | CCGTTTTC |
| chr12:80790108-80790208 | + | 44 | 1.78e-04 | AGCTTAATAG | GGAGATAATA | ACCTGCTTTG |
| chr4:29124150-29124250 | + | 42 | 1.78e-04 | TCCCTTTATA | GGTGATAAGT | CCACTAACAG |
| chr12:76941689-76941789 | - | 2 | 1.78e-04 | TTGTCTGCAT | AGAGATAATA | C |
| chr1:184669364-184669464 | + | 30 | 1.78e-04 | ATGTCCTGCA | TGAGATAAAG | TGAATGCCTA |
| chr4:130112948-130113048 | + | 13 | 1.78e-04 | TTGGACAGGC | ACAGATAACT | CACCCACCAA |
| chr5:100798575-100798675 | + | 49 | 1.95e-04 | GGAAGCGCGG | GAAGATAAAG | GTGCAGTGCA |
| chr8:117089958-117090058 | + | 50 | 1.95e-04 | GTAATTTAGA | TATGATAACA | CTATCTCCAA |
| chr14:118913743-118913843 | - | 90 | 1.95e-04 | A | ACTGATAAAG | ACCACATGTC |
| chr18:61960695-61960795 | + | 86 | 1.95e-04 | GCTGCTGTTC | TTTGATAACA | GCTGG |
| chr15:11399857-11399957 | - | 81 | 1.95e-04 | AGAGCGCATC | TGAGATAAAC | CAGAGCGCCA |
| chr2:37771930-37772030 | - | 45 | 1.95e-04 | GGAAGATGAC | AAAGATAAAG | GACAAACTGC |
| chr3:94526911-94527011 | + | 44 | 1.95e-04 | GATTGGCTCA | GCTGATAAAG | GCACTGCACT |
| chr7:16819975-16820075 | + | 48 | 2.08e-04 | TGGAGTGTGC | TGTGATAACG | AGTGGAAATG |
| chr1:144006685-144006785 | + | 69 | 2.08e-04 | CCTGAGATCT | GCTGATAAAC | AAAGCGTAGG |
| chr3:145590738-145590838 | + | 38 | 2.08e-04 | GTATTTGGGG | AAAGATAAAC | TGCCACCACA |
| chr10:93124454-93124554 | + | 78 | 2.08e-04 | CTGAGATGCA | GCTGATAAAC | GCCTCCACCT |
| chr4:123718285-123718385 | + | 30 | 2.08e-04 | GACGCTGACA | GCTGATAAAC | CGACTTTCCC |
| chr5:34930300-34930400 | - | 11 | 2.08e-04 | CATGGTAGAG | GGAGATAACT | GATTCTTGCA |
| chr6:34876128-34876228 | + | 29 | 2.24e-04 | CTCCCTCCTA | CTTGATAAGC | TCTCCCCGCC |
| chr19:32222270-32222370 | + | 26 | 2.24e-04 | AGTGTAACCG | TCAGATAACT | AACCATGGTT |
| chr6:38341809-38341909 | + | 46 | 2.24e-04 | CTCTGTGGTT | TCAGATAACT | GCTCCCACCA |
| chr13:20150271-20150371 | + | 22 | 2.24e-04 | GGTGTGCGGA | CTTGATAAGC | AGCCTATCAC |
| chr11:82847768-82847868 | - | 31 | 2.24e-04 | GTGTGGCTGG | TGAGATAATA | GGGGGACGCT |
| chr7:30169816-30169916 | - | 12 | 2.48e-04 | AAGCAGTATT | GCTGATAATA | GACAACAGCC |
| chr3:37546502-37546602 | - | 36 | 2.48e-04 | CGCTAAAGGT | GAAGATAATA | GCTACTATTG |
| chr12:105011655-105011755 | + | 72 | 2.65e-04 | GAGGGAGTTC | CAAGATAACC | AGGGCTACAC |
| chr8:105300153-105300253 | - | 69 | 2.65e-04 | GCTAATCCAC | CTAGATAACC | CCAAACTCCA |
| chr2:132076337-132076437 | + | 76 | 2.65e-04 | ACATCACTGC | TGAGATAACT | GAGGACCAGC |
| chr7:65640007-65640107 | + | 89 | 2.65e-04 | TACATAATGC | CGAGATAAAC | TC |
| chr3:37624816-37624916 | + | 20 | 2.65e-04 | GATCTCTGCT | GGAGATAATG | CTGACACAGA |
| chr10:71238009-71238109 | - | 32 | 2.65e-04 | GCGCGGCGGG | CATGATAACA | AGCCCCCGGA |
| chr18:88754956-88755056 | - | 55 | 2.65e-04 | ACGGAGTTGG | CATGATAACA | ACCCGCCAGT |
| chr14:79533488-79533588 | - | 21 | 2.81e-04 | ATGGGTAGGA | AAAGATAACT | GAGCTCTGAC |
| chr12:76710036-76710136 | - | 40 | 2.81e-04 | ATGTCCCTCC | TCAGATAATG | TTATTTATAT |
| chr19:44396904-44397004 | - | 45 | 2.81e-04 | TGTGCCTAGA | ACTGATAACT | AAACAGCTGA |
| chr10:41912482-41912582 | - | 71 | 2.81e-04 | CCTGAGGAGT | AAAGATAACT | GCAGTGAATG |
| chr7:141131063-141131163 | - | 76 | 2.97e-04 | ACTGGCTGGT | GTTGATAAAG | TTCCGAAGAC |
| chr17:27774012-27774112 | - | 62 | 2.97e-04 | CTAAGGACAG | GTTGATAAAG | CATTGTCATT |
| chr15:64310703-64310803 | - | 88 | 2.97e-04 | CTG | CCAGATAACT | TCTTCTCTGT |
| chr1:14484685-14484785 | + | 8 | 3.05e-04 | TATAGTG | AGTGATAACT | GGGTGTGCTG |
| chr1:82816070-82816170 | + | 12 | 3.05e-04 | ATCTGGAGTG | TGAGATAATG | CCGTTAAGCT |
| chr10:86026493-86026593 | + | 46 | 3.05e-04 | GCACATGGAA | GATGATAAAC | ACTCATATCA |
| chr2:32085363-32085463 | + | 56 | 3.05e-04 | CCATTAGCTT | CTAGATAAAG | GATAGTTTGT |
| chr2:131159566-131159666 | + | 7 | 3.05e-04 | TCCATC | TGAGATAATG | CCCTGCGCTC |
| chr5:134593271-134593371 | - | 42 | 3.23e-04 | CCTGGGCTGT | GAAGATAATG | GCTTCCAGGC |
| chr4:128760194-128760294 | - | 75 | 3.23e-04 | GTCTCTTCCT | TCTGATAACT | CATATCTCCA |
| chr12:55329070-55329170 | - | 49 | 3.23e-04 | GCCTCCCAGT | GCTGATAATG | CAGATGCACA |
| chr1:37479291-37479391 | + | 27 | 3.49e-04 | AGCTCCAAAT | CCAGATAATG | AAATACAACA |
| chr8:117032781-117032881 | + | 73 | 3.49e-04 | ATACAGCTCC | AAGGATAAGA | AGTGGGGGTA |
| chr6:58640791-58640891 | + | 57 | 3.49e-04 | ACTGTTTGGA | AAGGATAAGA | CTTCTTGAAT |
| chr13:34312449-34312549 | - | 83 | 3.49e-04 | GGCCAGAA | GAGGATAAGA | GAGCCCCTGG |
| chr16:87704585-87704685 | - | 89 | 3.49e-04 | GG | ATGGATAAGA | GTGGCTCCCA |
| chr14:47515830-47515930 | - | 12 | 3.49e-04 | CTGAGAAGTC | ACTGATAAAT | TACAAAACCG |
| chr7:75834765-75834865 | + | 30 | 3.64e-04 | CTCTCTGTCC | CCAGATAAAT | TCTTTTAACC |
| chr11:120303657-120303757 | + | 70 | 3.64e-04 | TTGCAGCCTC | CCAGATAATC | CTATCATTAC |
| chr1:4857707-4857807 | - | 74 | 3.83e-04 | CCGCGGCGGC | AGGGATAAGC | GCTCCGCCCC |
| chr2:152847820-152847920 | - | 74 | 3.83e-04 | GGCGGGACAA | GATGATAACT | GGCCAGAGCG |
| chr8:35589232-35589332 | - | 67 | 3.83e-04 | GACCCAGCCC | GGCGATAAGA | GAGGGCACTG |
| chr10:127290776-127290876 | - | 21 | 3.99e-04 | GTGTTAGGCT | CAAGATAACT | GACCTCAAGA |
| chr15:36727889-36727989 | + | 58 | 4.06e-04 | CTCCATTCTA | GGGGATAACA | GTGACTTTTA |
| chr4:40849690-40849790 | + | 54 | 4.06e-04 | AAGGTGGCAG | CATGATAAAG | AGGAATGCCT |
| chr5:118480125-118480225 | + | 68 | 4.24e-04 | ATAGCTGGGA | ATGGATAAGG | ACGATGTCTG |
| chr17:35165394-35165494 | - | 19 | 4.24e-04 | CATGACGTGA | TTTGATAACT | GGAAAGCCCC |
| chr9:108339161-108339261 | + | 2 | 4.45e-04 | G | CCGGATAAGG | CGGGACCCTG |
| chr11:80376292-80376392 | - | 85 | 4.45e-04 | CTATGG | CAAGATAATG | ATCTTTAAGG |
| chr2:121140435-121140535 | + | 7 | 4.45e-04 | GCCGCG | GTGGATAAGC | GGCGTGCGCC |
| chr2:79259440-79259540 | - | 61 | 4.61e-04 | CCTGAGCATG | CTAGATAAAT | GAGTACTCCT |
| chr6:43507104-43507204 | + | 77 | 4.61e-04 | TCTGGCAGTG | CCTGATAAAT | ACAGGAGTGG |
| chr2:4573053-4573153 | - | 60 | 4.61e-04 | CTCTTGAAGC | CAAGATAAAT | TTGATCTGGA |
| chr19:59261721-59261821 | + | 89 | 4.85e-04 | CCCTGTGACA | TCCGATAAGG | CC |
| chr1:53784310-53784410 | - | 65 | 4.85e-04 | CATACATGGT | ACCGATAACA | CCGAACCTCG |
| chr16:91729254-91729354 | - | 10 | 5.12e-04 | ATAGATGGGC | GGGGATAACC | GCCTGCCAA |
| chr2:173586727-173586827 | - | 57 | 6.54e-04 | AGTGGCCGGT | TGCGATAACG | GCTGCGCCCG |
| chr14:54426739-54426839 | + | 68 | 7.29e-04 | CCCACTTCCG | TACGATAACG | CAAGATGGTG |

#### Block Diagrams

The height of the motif "block" is proportional to -log(p-value), truncated at the height for a motif with a p-value of 1e-10.  
Click on any row to highlight sequence in all motifs. Mouse over the center of the motif blocks to see more information.

| Name | Lowest *p*-value | Motif Location |
| --- | --- | --- |
| chr1:167273957-167274057 | 5.71e-05 | +  - |
| chr18:88754956-88755056 | 2.65e-04 | +  - |
| chr7:64058092-64058192 | 2.29e-05 | +  - |
| chr11:120393791-120393891 | 2.29e-05 | +  - |
| chr5:145152785-145152885 | 1.15e-04 | +  - |
| chr16:58497980-58498080 | 7.28e-05 | +  - |
| chr2:181055406-181055506 | 1.39e-05 | +  - |
| chr10:41912482-41912582 | 2.81e-04 | +  - |
| chr3:60546330-60546430 | 3.49e-06 | +  - |
| chr12:101084027-101084127 | 8.95e-05 | +  - |
| chr6:143833202-143833302 | 3.78e-05 | +  - |
| chr11:73047749-73047849 | 1.67e-05 | +  - |
| chr4:40849690-40849790 | 4.06e-04 | +  - |
| chr12:84161266-84161366 | 3.78e-05 | +  - |
| chr1:53784310-53784410 | 4.85e-04 | +  - |
| chr2:181458507-181458607 | 1.75e-06 | +  - |
| chr15:97844710-97844810 | 2.29e-05 | +  - |
| chr8:35589232-35589332 | 3.83e-04 | +  - |
| chr3:94526911-94527011 | 1.95e-04 | +  - |
| chr8:71960207-71960307 | 4.34e-06 | +  - |
| chr5:34930300-34930400 | 2.08e-04 | +  - |
| chr13:8871701-8871801 | 1.39e-05 | +  - |
| chr9:67013624-67013724 | 7.28e-05 | +  - |
| chr8:14890864-14890964 | 4.90e-05 | +  - |
| chr11:32269108-32269208 | 7.04e-06 | +  - |
| chr15:64310703-64310803 | 2.97e-04 | +  - |
| chr18:38350130-38350230 | 1.39e-05 | +  - |
| chr5:73354169-73354269 | 3.78e-05 | +  - |
| chr9:115518039-115518139 | 8.95e-05 | +  - |
| chr4:154541753-154541853 | 3.78e-05 | +  - |
| chr11:86758157-86758257 | 1.66e-04 | +  - |
| chr7:142087231-142087331 | 7.28e-05 | +  - |
| chr2:170128845-170128945 | 7.28e-05 | +  - |
| chr19:44396904-44397004 | 2.81e-04 | +  - |
| chr14:21823238-21823338 | 1.39e-05 | +  - |
| chr13:97453145-97453245 | 2.98e-05 | +  - |
| chr2:131159566-131159666 | 3.05e-04 | +  - |
| chr4:123718285-123718385 | 2.08e-04 | +  - |
| chr2:37771930-37772030 | 1.95e-04 | +  - |
| chr12:76710036-76710136 | 2.81e-04 | +  - |
| chr10:93124454-93124554 | 2.08e-04 | +  - |
| chr14:47515830-47515930 | 3.49e-04 | +  - |
| chr6:32534120-32534220 | 1.67e-05 | +  - |
| chr4:144981198-144981298 | 7.28e-05 | +  - |
| chr3:130810201-130810301 | 8.95e-05 | +  - |
| chr17:5099381-5099481 | 1.67e-05 | +  - |
| chr9:21384160-21384260 | 4.90e-05 | +  - |
| chr12:40036729-40036829 | 1.75e-06 | +  - |
| chr3:79580337-79580437 | 1.02e-04 | +  - |
| chr9:44343366-44343466 | 2.29e-05 | +  - |
| chr4:119198681-119198781 | 1.53e-04 | +  - |
| chr16:91865025-91865125 | 7.28e-05 | +  - |
| chr13:51976267-51976367 | 1.39e-05 | +  - |
| chr7:44483007-44483107 | 1.39e-05 | +  - |
| chr9:3328343-3328443 | 1.39e-05 | +  - |
| chr9:122104015-122104115 | 1.30e-04 | +  - |
| chr17:27774012-27774112 | 2.97e-04 | +  - |
| chr12:55329070-55329170 | 3.23e-04 | +  - |
| chr9:66347548-66347648 | 1.75e-06 | +  - |
| chrX:159597637-159597737 | 1.15e-04 | +  - |
| chr7:19632888-19632988 | 3.78e-05 | +  - |
| chr10:81352088-81352188 | 7.28e-05 | +  - |
| chr12:100958677-100958777 | 1.30e-04 | +  - |
| chr11:48838510-48838610 | 1.39e-05 | +  - |
| chr4:89510153-89510253 | 2.98e-05 | +  - |
| chr10:71238009-71238109 | 2.65e-04 | +  - |
| chr16:21357574-21357674 | 2.72e-05 | +  - |
| chr11:82847768-82847868 | 2.24e-04 | +  - |
| chr13:97066815-97066915 | 1.39e-05 | +  - |
| chr8:85526315-85526415 | 2.29e-05 | +  - |
| chr18:35619702-35619802 | 1.30e-04 | +  - |
| chr17:71681163-71681263 | 1.67e-05 | +  - |
| chr3:51387110-51387210 | 1.66e-04 | +  - |
| chr2:132695865-132695965 | 3.78e-05 | +  - |
| chr6:38831161-38831261 | 1.02e-04 | +  - |
| chr1:87712410-87712510 | 8.95e-05 | +  - |
| chr13:57659949-57660049 | 1.30e-04 | +  - |
| chr2:28323025-28323125 | 7.04e-06 | +  - |
| chr19:55865965-55866065 | 1.75e-06 | +  - |
| chr11:11835494-11835594 | 1.66e-04 | +  - |
| chr2:32085363-32085463 | 3.05e-04 | +  - |
| chr7:128672450-128672550 | 2.29e-05 | +  - |
| chr17:47210549-47210649 | 1.66e-04 | +  - |
| chr5:37456736-37456836 | 8.95e-05 | +  - |
| chr13:20150271-20150371 | 2.24e-04 | +  - |
| chr17:79926012-79926112 | 1.02e-04 | +  - |
| chr15:11399857-11399957 | 1.95e-04 | +  - |
| chr5:118489137-118489237 | 1.39e-05 | +  - |
| chr14:70107111-70107211 | 3.49e-06 | +  - |
| chr4:46075811-46075911 | 1.66e-04 | +  - |
| chr15:79741596-79741696 | 3.78e-05 | +  - |
| chr10:86026493-86026593 | 3.05e-04 | +  - |
| chr14:76520693-76520793 | 2.29e-05 | +  - |
| chr4:128760194-128760294 | 3.23e-04 | +  - |
| chr11:61067579-61067679 | 1.30e-04 | +  - |
| chr4:33450487-33450587 | 7.04e-06 | +  - |
| chr2:121140435-121140535 | 4.45e-04 | +  - |
| chr3:145590738-145590838 | 2.08e-04 | +  - |
| chr14:79533488-79533588 | 2.81e-04 | +  - |
| chr17:30347302-30347402 | 3.78e-05 | +  - |
| chr6:123293105-123293205 | 1.67e-05 | +  - |
| chr16:34031700-34031800 | 7.28e-05 | +  - |
| chr19:9899580-9899680 | 1.39e-05 | +  - |
| chr1:82816070-82816170 | 3.05e-04 | +  - |
| chr6:38341809-38341909 | 2.24e-04 | +  - |
| chr16:87704585-87704685 | 3.49e-04 | +  - |
| chr3:88325269-88325369 | 2.98e-05 | +  - |
| chr10:77210625-77210725 | 2.29e-05 | +  - |
| chr14:47116162-47116262 | 2.98e-05 | +  - |
| chr7:115831009-115831109 | 1.39e-05 | +  - |
| chr13:24681926-24682026 | 8.24e-05 | +  - |
| chr2:172298240-172298340 | 1.53e-04 | +  - |
| chr19:32222270-32222370 | 2.24e-04 | +  - |
| chr8:94964944-94965044 | 1.53e-04 | +  - |
| chr13:73443549-73443649 | 1.53e-04 | +  - |
| chr6:86364837-86364937 | 1.67e-05 | +  - |
| chr16:90197429-90197529 | 1.15e-04 | +  - |
| chr3:122113598-122113698 | 1.15e-04 | +  - |
| chr11:96075674-96075774 | 8.24e-05 | +  - |
| chr19:4878888-4878988 | 4.90e-05 | +  - |
| chr11:75234827-75234927 | 3.78e-05 | +  - |
| chr5:143819088-143819188 | 8.24e-05 | +  - |
| chr2:4573053-4573153 | 4.61e-04 | +  - |
| chr6:134984894-134984994 | 7.04e-06 | +  - |
| chr2:166665721-166665821 | 1.15e-04 | +  - |
| chr8:122330606-122330706 | 1.15e-04 | +  - |
| chr18:61960695-61960795 | 1.95e-04 | +  - |
| chr6:125104401-125104501 | 5.71e-05 | +  - |
| chr8:60900206-60900306 | 3.49e-06 | +  - |
| chr14:118913743-118913843 | 1.95e-04 | +  - |
| chr1:3435154-3435254 | 4.34e-06 | +  - |
| chr2:93443949-93444049 | 2.72e-05 | +  - |
| chr10:59875541-59875641 | 8.24e-05 | +  - |
| chr7:141131063-141131163 | 2.97e-04 | +  - |
| chr11:23313687-23313787 | 1.39e-05 | +  - |
| chr7:115988546-115988646 | 3.78e-05 | +  - |
| chr3:96082800-96082900 | 1.75e-06 | +  - |
| chr4:42880272-42880372 | 5.71e-05 | +  - |
| chr14:73322227-73322327 | 4.90e-05 | +  - |
| chr7:16412641-16412741 | 8.95e-05 | +  - |
| chr9:103288322-103288422 | 4.90e-05 | +  - |
| chr15:27497059-27497159 | 1.67e-05 | +  - |
| chr16:32541427-32541527 | 1.67e-05 | +  - |
| chr2:151011403-151011503 | 1.39e-05 | +  - |
| chr12:71904407-71904507 | 1.53e-04 | +  - |
| chr9:75547297-75547397 | 1.67e-05 | +  - |
| chr8:117089958-117090058 | 1.95e-04 | +  - |
| chr16:49771816-49771916 | 7.28e-05 | +  - |
| chr2:153525950-153526050 | 8.24e-05 | +  - |
| chr2:152741639-152741739 | 1.67e-05 | +  - |
| chr4:41026584-41026684 | 1.75e-06 | +  - |
| chr5:100798575-100798675 | 1.95e-04 | +  - |
| chr9:59271788-59271888 | 1.30e-04 | +  - |
| chr16:91729254-91729354 | 5.12e-04 | +  - |
| chr2:19024069-19024169 | 3.49e-06 | +  - |
| chr3:37546502-37546602 | 2.48e-04 | +  - |
| chr11:109557500-109557600 | 5.71e-05 | +  - |
| chr4:126803606-126803706 | 3.78e-05 | +  - |
| chr3:37624816-37624916 | 2.65e-04 | +  - |
| chr16:17220559-17220659 | 1.67e-05 | +  - |
| chr18:44335469-44335569 | 2.98e-05 | +  - |
| chr5:130029677-130029777 | 8.24e-05 | +  - |
| chr14:79530302-79530402 | 1.15e-04 | +  - |
| chr17:47598269-47598369 | 7.28e-05 | +  - |
| chr2:11212331-11212431 | 8.24e-05 | +  - |
| chr7:27454859-27454959 | 1.02e-04 | +  - |
| chr6:122394334-122394434 | 3.49e-06 | +  - |
| chr14:52306274-52306374 | 1.67e-05 | +  - |
| chr2:28418164-28418264 | 4.90e-05 | +  - |
| chr11:85298177-85298277 | 1.15e-04 | +  - |
| chr18:10092913-10093013 | 5.71e-05 | +  - |
| chr17:24628796-24628896 | 4.90e-05 | +  - |
| chr13:31909533-31909633 | 3.49e-06 | +  - |
| chr12:71164592-71164692 | 2.29e-05 | +  - |
| chr2:119163310-119163410 | 1.30e-04 | +  - |
| chr1:159238253-159238353 | 1.15e-04 | +  - |
| chr5:31733120-31733220 | 7.28e-05 | +  - |
| chr12:32143280-32143380 | 7.28e-05 | +  - |
| chr14:54426739-54426839 | 7.29e-04 | +  - |
| chr4:130112948-130113048 | 1.78e-04 | +  - |
| chr2:60223668-60223768 | 2.29e-05 | +  - |
| chr15:53202459-53202559 | 3.49e-06 | +  - |
| chr10:108582493-108582593 | 1.39e-05 | +  - |
| chr5:50126334-50126434 | 4.90e-05 | +  - |
| chr14:21549388-21549488 | 3.49e-06 | +  - |
| chr11:80376292-80376392 | 4.45e-04 | +  - |
| chr6:116350164-116350264 | 7.28e-05 | +  - |
| chr11:95835267-95835367 | 8.95e-05 | +  - |
| chr7:65640007-65640107 | 2.65e-04 | +  - |
| chr15:78268012-78268112 | 1.75e-06 | +  - |
| chr2:132076337-132076437 | 2.65e-04 | +  - |
| chr18:82568460-82568560 | 1.67e-05 | +  - |
| chr6:34876128-34876228 | 2.24e-04 | +  - |
| chr9:57070405-57070505 | 7.28e-05 | +  - |
| chr15:37100038-37100138 | 4.90e-05 | +  - |
| chr15:80130822-80130922 | 1.53e-04 | +  - |
| chr6:120174624-120174724 | 8.24e-05 | +  - |
| chr17:79365207-79365307 | 1.30e-04 | +  - |
| chr11:69258426-69258526 | 1.15e-04 | +  - |
| chr9:66895075-66895175 | 1.66e-04 | +  - |
| chr7:30169816-30169916 | 2.48e-04 | +  - |
| chr9:72902765-72902865 | 1.39e-05 | +  - |
| chr17:84926353-84926453 | 8.95e-05 | +  - |
| chr9:108339161-108339261 | 4.45e-04 | +  - |
| chr4:136653119-136653219 | 1.75e-06 | +  - |
| chr7:125472198-125472298 | 4.90e-05 | +  - |
| chr6:28710210-28710310 | 1.02e-04 | +  - |
| chr11:52115665-52115765 | 4.90e-05 | +  - |
| chr10:93679867-93679967 | 2.98e-05 | +  - |
| chr11:69589369-69589469 | 1.75e-06 | +  - |
| chr11:68884524-68884624 | 3.49e-06 | +  - |
| chr17:35165394-35165494 | 4.24e-04 | +  - |
| chr1:14484685-14484785 | 3.05e-04 | +  - |
| chr11:60486424-60486524 | 2.98e-05 | +  - |
| chr18:32556803-32556903 | 2.72e-05 | +  - |
| chr13:101573340-101573440 | 3.78e-05 | +  - |
| chr1:184669364-184669464 | 1.78e-04 | +  - |
| chr11:116615802-116615902 | 8.95e-05 | +  - |
| chr18:67246200-67246300 | 1.67e-05 | +  - |
| chr9:76236716-76236816 | 3.78e-05 | +  - |
| chr9:64779736-64779836 | 8.24e-05 | +  - |
| chr4:103120154-103120254 | 1.39e-05 | +  - |
| chr16:76370166-76370266 | 1.02e-04 | +  - |
| chr6:43507104-43507204 | 4.61e-04 | +  - |
| chr5:120599783-120599883 | 4.90e-05 | +  - |
| chr4:134663256-134663356 | 2.98e-05 | +  - |
| chr7:97589986-97590086 | 1.39e-05 | +  - |
| chr5:35035873-35035973 | 1.39e-05 | +  - |
| chr10:70133348-70133448 | 3.49e-06 | +  - |
| chr11:90664423-90664523 | 1.53e-04 | +  - |
| chr2:174297079-174297179 | 7.28e-05 | +  - |
| chr6:53134583-53134683 | 1.67e-05 | +  - |
| chr9:99242846-99242946 | 1.39e-05 | +  - |
| chr4:132530565-132530665 | 1.53e-04 | +  - |
| chr10:77232495-77232595 | 4.90e-05 | +  - |
| chr15:82342048-82342148 | 2.72e-05 | +  - |
| chr11:79008560-79008660 | 3.49e-06 | +  - |
| chr19:7552151-7552251 | 1.53e-04 | +  - |
| chr7:110920405-110920505 | 4.90e-05 | +  - |
| chr19:6284558-6284658 | 2.72e-05 | +  - |
| chr1:136014964-136015064 | 3.78e-05 | +  - |
| chr2:28032679-28032779 | 1.53e-04 | +  - |
| chrX:7837292-7837392 | 7.04e-06 | +  - |
| chr5:24927981-24928081 | 1.67e-05 | +  - |
| chr17:83489251-83489351 | 8.24e-05 | +  - |
| chr2:173586727-173586827 | 6.54e-04 | +  - |
| chr1:75450526-75450626 | 3.78e-05 | +  - |
| chr19:40977065-40977165 | 2.29e-05 | +  - |
| chr3:55080176-55080276 | 1.66e-04 | +  - |
| chr4:124645091-124645191 | 2.98e-05 | +  - |
| chr12:76941689-76941789 | 1.78e-04 | +  - |
| chr10:40328063-40328163 | 1.66e-04 | +  - |
| chr4:134924511-134924611 | 7.28e-05 | +  - |
| chr1:144006685-144006785 | 2.08e-04 | +  - |
| chr18:82634085-82634185 | 8.24e-05 | +  - |
| chr2:118660266-118660366 | 2.29e-05 | +  - |
| chr1:9782189-9782289 | 4.90e-05 | +  - |
| chr6:38921194-38921294 | 1.30e-04 | +  - |
| chr13:34312449-34312549 | 3.49e-04 | +  - |
| chr7:122015208-122015308 | 1.39e-05 | +  - |
| chr11:117308077-117308177 | 5.71e-05 | +  - |
| chr12:12427396-12427496 | 7.28e-05 | +  - |
| chr16:90144449-90144549 | 1.02e-04 | +  - |
| chr10:94073013-94073113 | 2.29e-05 | +  - |
| chr12:83073377-83073477 | 1.30e-04 | +  - |
| chr8:122296354-122296454 | 2.29e-05 | +  - |
| chr4:29124150-29124250 | 1.78e-04 | +  - |
| chr2:79259440-79259540 | 4.61e-04 | +  - |
| chr9:96254978-96255078 | 7.28e-05 | +  - |
| chr4:41129997-41130097 | 1.53e-04 | +  - |
| chr7:102958803-102958903 | 1.39e-05 | +  - |
| chr8:105300153-105300253 | 2.65e-04 | +  - |
| chr15:36727889-36727989 | 4.06e-04 | +  - |
| chr18:32024987-32025087 | 4.90e-05 | +  - |
| chr6:119361106-119361206 | 1.02e-04 | +  - |
| chr6:29583606-29583706 | 1.39e-05 | +  - |
| chr3:75918202-75918302 | 2.98e-05 | +  - |
| chr2:152847820-152847920 | 3.83e-04 | +  - |
| chr15:38301922-38302022 | 1.15e-04 | +  - |
| chr7:16819975-16820075 | 2.08e-04 | +  - |
| chr6:38757779-38757879 | 4.90e-05 | +  - |
| chr9:45803597-45803697 | 7.28e-05 | +  - |
| chr11:60914061-60914161 | 1.30e-04 | +  - |
| chr5:137116487-137116587 | 1.67e-05 | +  - |
| chr19:56543498-56543598 | 1.15e-04 | +  - |
| chr19:53277633-53277733 | 1.39e-05 | +  - |
| chr13:43191907-43192007 | 1.39e-05 | +  - |
| chr10:127290776-127290876 | 3.99e-04 | +  - |
| chr19:59261721-59261821 | 4.85e-04 | +  - |
| chr6:58640791-58640891 | 3.49e-04 | +  - |
| chr10:127126665-127126765 | 4.34e-06 | +  - |
| chr14:63067779-63067879 | 5.71e-05 | +  - |
| chr17:46679988-46680088 | 1.39e-05 | +  - |
| chr2:120969738-120969838 | 1.15e-04 | +  - |
| chr11:120303657-120303757 | 3.64e-04 | +  - |
| chr7:132492076-132492176 | 1.39e-05 | +  - |
| chr13:95965321-95965421 | 1.39e-05 | +  - |
| chr6:5287482-5287582 | 1.15e-04 | +  - |
| chr5:118480125-118480225 | 4.24e-04 | +  - |
| chr12:111518050-111518150 | 1.15e-04 | +  - |
| chr12:105019665-105019765 | 1.67e-05 | +  - |
| chr2:154634391-154634491 | 3.78e-05 | +  - |
| chr8:117032781-117032881 | 3.49e-04 | +  - |
| chr5:134593271-134593371 | 3.23e-04 | +  - |
| chr1:37479291-37479391 | 3.49e-04 | +  - |
| chr17:29658328-29658428 | 5.71e-05 | +  - |
| chr14:65145074-65145174 | 1.15e-04 | +  - |
| chr11:105366112-105366212 | 3.49e-06 | +  - |
| chr17:24527396-24527496 | 4.90e-05 | +  - |
| chr6:120530699-120530799 | 1.53e-04 | +  - |
| chr12:80790108-80790208 | 1.78e-04 | +  - |
| chr1:157695418-157695518 | 1.78e-04 | +  - |
| chr1:4857707-4857807 | 3.83e-04 | +  - |
| chr7:75834765-75834865 | 3.64e-04 | +  - |
| chr12:105011655-105011755 | 2.65e-04 | +  - |
| chr12:8609275-8609375 | 3.78e-05 | +  - |
| chr12:80930283-80930383 | 7.28e-05 | +  - |
|  | | 0      20      40      60      80      100 |

Time 474.9 secs.

|  |  |
| --- | --- |
| Motif 2 | Previous Next Top |

| Summary | Sequence Logo |
| --- | --- |
| E-value \| 6.6e-182; Width \| 15; Sites \| 71 show more Log Likelihood Ratio \|; \| 1089; Information Content \|; \| 22.0 (bits); Relative Entropy \|; \| 22.1 (bits) show less | StandardReverse Complement   Download LOGO      Orientation: standard reverse complement    SSC: off on    Format: web (png) publication (eps)    Width:  cm    Height:  cm |

#### Regular expression

ACACACA[TC]ACACACA

#### Further Analysis

Submit this motif to
 


    
 


    
 


    
 


    
 


 Mouse-over buttons for more information.

#### Data Formats

View the motif in
PSPM Format 


    
PSSM Format 


    
BLOCKS Format 


    
FASTA Format 


    
Raw Format 


    
or
Hide  
This feature requires javascript.

#### Sites

Click on any row to highlight sequence in all motifs.

| Name | Strand | Start | *p*-value | Sites | | |
| --- | --- | --- | --- | --- | --- | --- |
| chr1:136062109-136062209 | - | 84 | 8.51e-10 | AC | ACACACATACACACA | CACTCACCAC |
| chr10:11123760-11123860 | - | 54 | 8.51e-10 | GTACACACAC | ACACACATACACACA | TACACATATG |
| chr11:98417533-98417633 | - | 58 | 8.51e-10 | CATGGGCACA | ACACACATACACACA | TATACATGGA |
| chr4:154191336-154191436 | + | 73 | 8.51e-10 | CACAAACACC | ACACACATACACACA | CAGCACACAG |
| chr15:101296982-101297082 | - | 14 | 8.51e-10 | GTGCACACGC | ACACACATACACACA | CTCACACATT |
| chr16:75291794-75291894 | - | 61 | 8.51e-10 | GCACACATAC | ACACACATACACACA | TACACACACA |
| chr15:11429043-11429143 | - | 9 | 8.51e-10 | ACATACACAC | ACACACATACACACA | CACACACA |
| chr3:130124484-130124584 | + | 72 | 8.51e-10 | ACACACTCAC | ACACACATACACACA | TACACATGCA |
| chr17:28383401-28383501 | - | 19 | 8.51e-10 | TCAGTCACAC | ACACACATACACACA | CAATCAGTCA |
| chr17:29116579-29116679 | - | 80 | 8.51e-10 | ATTCAC | ACACACATACACACA | TTCACACATA |
| chr6:137792127-137792227 | + | 56 | 8.51e-10 | ACATGCACGC | ACACACATACACACA | CATACACACA |
| chr5:97280498-97280598 | - | 16 | 8.51e-10 | ACTCACTCAC | ACACACATACACACA | CTCACACAGA |
| chr8:126132997-126133097 | - | 31 | 8.51e-10 | ACACACATAT | ACACACATACACACA | CATGCATACA |
| chr6:34325635-34325735 | - | 6 | 1.75e-09 | ACACACATGC | ACACACACACACACA | TGCAC |
| chr4:41129997-41130097 | - | 85 | 1.75e-09 | C | ACACACACACACACA | GAGAGAGAGA |
| chr1:154798318-154798418 | + | 77 | 1.75e-09 | CACCTAGCTC | ACACACACACACACA | CACATACAC |
| chr11:51555870-51555970 | - | 7 | 1.75e-09 | ACACACACAC | ACACACACACACACA | CTCATT |
| chr12:12370982-12371082 | - | 68 | 1.75e-09 | ACACACACGG | ACACACACACACACA | GAGACACACA |
| chr13:25050499-25050599 | - | 1 | 1.75e-09 | TCAGCCACAC | ACACACACACACACA |  |
| chr10:118908786-118908886 | - | 44 | 1.75e-09 | ACACAGACAC | ACACACACACACACA | TGCACACACA |
| chr8:44734948-44735048 | + | 10 | 1.75e-09 | TGCACATGC | ACACACACACACACA | CACTAGTGCA |
| chr11:33863308-33863408 | + | 68 | 1.75e-09 | ACGTGCGCGC | ACACACACACACACA | TACATATACA |
| chr10:75570144-75570244 | - | 11 | 1.75e-09 | GCACATGCAC | ACACACACACACACA | ATTGCACATA |
| chr7:29034256-29034356 | + | 78 | 1.75e-09 | ACACCACACC | ACACACACACACACA | CCAACACA |
| chr1:191198864-191198964 | - | 63 | 1.75e-09 | GCACATACAC | ACACACACACACACA | CGCATGCACT |
| chr15:83991240-83991340 | + | 56 | 1.75e-09 | CATACATATC | ACACACACACACACA | CACACACACA |
| chr5:120320367-120320467 | + | 25 | 2.65e-09 | CACACATCTC | ACACACATGCACACA | CTCACACGTG |
| chr14:79915824-79915924 | - | 75 | 2.65e-09 | ACAGAGACAT | ACACACATGCACACA | CAGAGACACA |
| chr14:8006859-8006959 | + | 8 | 2.65e-09 | CACACAG | ACACACATGCACACA | CACCACACAC |
| chr18:81664637-81664737 | - | 66 | 3.46e-09 | ACTCACACAC | ACATACATACACACA | TATACACACT |
| chr4:21829354-21829454 | - | 58 | 6.21e-09 | ACAAAGACAC | ACACACAGACACACA | CAGACACAGA |
| chr9:45278995-45279095 | + | 7 | 7.96e-09 | ACAGTC | ACATACACACACACA | TAGACACACA |
| chr2:172484480-172484580 | + | 37 | 7.96e-09 | ACAGACAGAC | ACACGCATACACACA | CACGCATACA |
| chr8:121438945-121439045 | + | 58 | 1.04e-08 | GTGCACACAC | ACACACATACATACA | TACACAGCAC |
| chr16:9738087-9738187 | - | 47 | 1.04e-08 | GCACGCGCAC | ACACACATACACATA | CACGCACACA |
| chr9:50683147-50683247 | + | 69 | 1.75e-08 | ACACACTCAC | TCACACACACACACA | CTCACACACA |
| chr7:44483007-44483107 | + | 85 | 1.75e-08 | TCTCTCTCTC | TCACACACACACACA | C |
| chr19:16767067-16767167 | + | 25 | 2.10e-08 | ACTCACACAG | ACACACACTCACACA | CACTCACACA |
| chr11:109325648-109325748 | + | 53 | 2.10e-08 | ACACACTCAC | ACACACACTCACACA | TCCTCACACA |
| chr1:37036110-37036210 | - | 19 | 2.18e-08 | ACATGCATGC | ACACACATATACACA | CATGCACACT |
| chr4:108881520-108881620 | + | 14 | 3.61e-08 | CATACACACC | ACACACATGCATACA | CACATCCATA |
| chr9:120355823-120355923 | - | 23 | 3.61e-08 | ATGCACATGG | TCACACATGCACACA | CCTACACATG |
| chr4:129091654-129091754 | + | 79 | 3.61e-08 | GTTTCTATAC | ACACACATGCACATA | CCTGAGT |
| chr5:118317192-118317292 | - | 28 | 5.01e-08 | AGACAGACAG | ACACACATACACAAA | TGTACACACA |
| chr9:60539702-60539802 | + | 42 | 5.01e-08 | ATACACGCAC | ACACGCACGCACACA | CACATGCACA |
| chr17:3307322-3307422 | + | 22 | 6.85e-08 | ATATACAGAC | ACACACAGACATACA | GAACCATATA |
| chr5:146424152-146424252 | - | 37 | 7.73e-08 | ACACACATGC | ACATACACACATACA | CATGGGCACA |
| chr2:93390201-93390301 | - | 35 | 8.40e-08 | GCCCAGCCCC | AAACACAGACACACA | AGTCTATCGG |
| chr2:177305052-177305152 | + | 1 | 9.22e-08 |  | GCACACATACACCCA | CATAAACATG |
| chr10:94264745-94264845 | - | 1 | 1.12e-07 | AAAGACAGAC | ACACGCACACACATA |  |
| chr13:110070280-110070380 | - | 6 | 1.53e-07 | ACACACTCAC | TCACACACTCACACA | CTCAC |
| chr9:14988798-14988898 | + | 10 | 1.53e-07 | GCATACACT | GCACACATACACACC | ATCATGCCAT |
| chr18:34164850-34164950 | + | 58 | 3.17e-07 | ACACACATGC | TCATGCATACACACA | TGCTCCTGCA |
| chr6:120530699-120530799 | + | 30 | 3.96e-07 | TCTCCCTCTG | TCTCACATACACACA | ACACATGATG |
| chr17:8365907-8366007 | - | 33 | 4.30e-07 | ACGCATCGAC | ATATACATGCACACA | CCACACACAC |
| chr2:31449952-31450052 | - | 82 | 4.30e-07 | ACCT | ATATACATGCACACA | CATGCATCTG |
| chr13:38338228-38338328 | - | 11 | 5.36e-07 | ACAGACACAG | ACACAGACACAGACA | CAGACAGACA |
| chr2:91664865-91664965 | + | 22 | 5.79e-07 | CACATACCAC | ACACACATCCAGACA | GATAGACAGA |
| chr1:37479291-37479391 | + | 49 | 9.22e-07 | ATACAACAGG | AAACACACACACCTA | ACAGTCACTG |
| chr8:77688089-77688189 | - | 12 | 9.99e-07 | GTGCCTGCTC | ACATGCCCACACACA | GGGACTCTAC |
| chr6:39012219-39012319 | + | 60 | 1.31e-06 | CTGTGCACTC | GCACACTCACATACA | TGCTAAGTAG |
| chr7:142087231-142087331 | + | 50 | 1.31e-06 | GAGGCTCAGG | GCACATAGACACACA | GATATGCTTG |
| chr7:111004284-111004384 | - | 36 | 1.50e-06 | CCATATCTGA | GCCCACAGACACACA | GGGAAGCAAT |
| chr2:35609292-35609392 | - | 85 | 1.94e-06 | T | ACACGGATACACACC | CGTCCACCCT |
| chr2:180059847-180059947 | - | 9 | 1.94e-06 | CAGGAGGAAC | ACACACATTTACACC | ACGGAGGG |
| chr3:145590738-145590838 | - | 5 | 2.06e-06 | ACTGACAAGT | AAACACCCACACCCA | CCCA |
| chr16:75305611-75305711 | - | 46 | 3.54e-06 | CAGCTACCAG | GCACACATACAGCTA | TGAGGCACAC |
| chr10:79975648-79975748 | + | 68 | 3.54e-06 | AGCTTCCCCG | AGATGCACGCACACA | TGCGTCGCCA |
| chr13:51976267-51976367 | + | 14 | 3.76e-06 | CAGAGGGAGA | GCATGCATGCACCCA | GGGGACCCGC |
| chr19:55865965-55866065 | + | 82 | 7.36e-06 | CTTGGCTACT | GAATACTTGCACACA | CACT |
| chr6:38831161-38831261 | - | 18 | 8.60e-06 | GGAGAAATCT | AAACACATGTATATA | CAGGGAGGAC |

#### Block Diagrams

The height of the motif "block" is proportional to -log(p-value), truncated at the height for a motif with a p-value of 1e-10.  
Click on any row to highlight sequence in all motifs. Mouse over the center of the motif blocks to see more information.

| Name | Lowest *p*-value | Motif Location |
| --- | --- | --- |
| chr16:9738087-9738187 | 1.04e-08 | +  - |
| chr8:126132997-126133097 | 8.51e-10 | +  - |
| chr5:146424152-146424252 | 7.73e-08 | +  - |
| chr7:142087231-142087331 | 1.31e-06 | +  - |
| chr2:180059847-180059947 | 1.94e-06 | +  - |
| chr14:8006859-8006959 | 2.65e-09 | +  - |
| chr13:51976267-51976367 | 3.76e-06 | +  - |
| chr15:83991240-83991340 | 1.75e-09 | +  - |
| chr7:44483007-44483107 | 1.75e-08 | +  - |
| chr4:129091654-129091754 | 3.61e-08 | +  - |
| chr5:97280498-97280598 | 8.51e-10 | +  - |
| chr6:38831161-38831261 | 8.60e-06 | +  - |
| chr19:55865965-55866065 | 7.36e-06 | +  - |
| chr18:81664637-81664737 | 3.46e-09 | +  - |
| chr3:145590738-145590838 | 2.06e-06 | +  - |
| chr6:137792127-137792227 | 8.51e-10 | +  - |
| chr17:3307322-3307422 | 6.85e-08 | +  - |
| chr1:191198864-191198964 | 1.75e-09 | +  - |
| chr9:14988798-14988898 | 1.53e-07 | +  - |
| chr2:172484480-172484580 | 7.96e-09 | +  - |
| chr10:79975648-79975748 | 3.54e-06 | +  - |
| chr17:29116579-29116679 | 8.51e-10 | +  - |
| chr7:29034256-29034356 | 1.75e-09 | +  - |
| chr17:28383401-28383501 | 8.51e-10 | +  - |
| chr9:120355823-120355923 | 3.61e-08 | +  - |
| chr9:50683147-50683247 | 1.75e-08 | +  - |
| chr1:37036110-37036210 | 2.18e-08 | +  - |
| chr13:110070280-110070380 | 1.53e-07 | +  - |
| chr10:75570144-75570244 | 1.75e-09 | +  - |
| chr3:130124484-130124584 | 8.51e-10 | +  - |
| chr15:11429043-11429143 | 8.51e-10 | +  - |
| chr16:75291794-75291894 | 8.51e-10 | +  - |
| chr8:77688089-77688189 | 9.99e-07 | +  - |
| chr7:111004284-111004384 | 1.50e-06 | +  - |
| chr2:177305052-177305152 | 9.22e-08 | +  - |
| chr2:31449952-31450052 | 4.30e-07 | +  - |
| chr15:101296982-101297082 | 8.51e-10 | +  - |
| chr4:108881520-108881620 | 3.61e-08 | +  - |
| chr11:33863308-33863408 | 1.75e-09 | +  - |
| chr4:154191336-154191436 | 8.51e-10 | +  - |
| chr17:8365907-8366007 | 4.30e-07 | +  - |
| chr11:109325648-109325748 | 2.10e-08 | +  - |
| chr8:121438945-121439045 | 1.04e-08 | +  - |
| chr8:44734948-44735048 | 1.75e-09 | +  - |
| chr10:118908786-118908886 | 1.75e-09 | +  - |
| chr9:60539702-60539802 | 5.01e-08 | +  - |
| chr13:25050499-25050599 | 1.75e-09 | +  - |
| chr14:79915824-79915924 | 2.65e-09 | +  - |
| chr18:34164850-34164950 | 3.17e-07 | +  - |
| chr16:75305611-75305711 | 3.54e-06 | +  - |
| chr12:12370982-12371082 | 1.75e-09 | +  - |
| chr2:91664865-91664965 | 5.79e-07 | +  - |
| chr11:51555870-51555970 | 1.75e-09 | +  - |
| chr9:45278995-45279095 | 7.96e-09 | +  - |
| chr11:98417533-98417633 | 8.51e-10 | +  - |
| chr1:154798318-154798418 | 1.75e-09 | +  - |
| chr4:21829354-21829454 | 6.21e-09 | +  - |
| chr4:41129997-41130097 | 1.75e-09 | +  - |
| chr6:39012219-39012319 | 1.31e-06 | +  - |
| chr5:118317192-118317292 | 5.01e-08 | +  - |
| chr5:120320367-120320467 | 2.65e-09 | +  - |
| chr6:34325635-34325735 | 1.75e-09 | +  - |
| chr2:35609292-35609392 | 1.94e-06 | +  - |
| chr10:11123760-11123860 | 8.51e-10 | +  - |
| chr19:16767067-16767167 | 2.10e-08 | +  - |
| chr1:37479291-37479391 | 9.22e-07 | +  - |
| chr2:93390201-93390301 | 8.40e-08 | +  - |
| chr10:94264745-94264845 | 1.12e-07 | +  - |
| chr13:38338228-38338328 | 5.36e-07 | +  - |
| chr6:120530699-120530799 | 3.96e-07 | +  - |
| chr1:136062109-136062209 | 8.51e-10 | +  - |
|  | | 0      20      40      60      80      100 |

Time 894.7 secs.

|  |  |
| --- | --- |
| Motif 3 | Previous Top |

| Summary | Sequence Logo |
| --- | --- |
| E-value \| 4.7e-160; Width \| 19; Sites \| 51 show more Log Likelihood Ratio \|; \| 911; Information Content \|; \| 25.7 (bits); Relative Entropy \|; \| 25.8 (bits) show less | StandardReverse Complement   Download LOGO      Orientation: standard reverse complement    SSC: off on    Format: web (png) publication (eps)    Width:  cm    Height:  cm |

#### Regular expression

GTGT[GA]T[GA]TG[TC][AG]TGTGTGTG

#### Further Analysis

Submit this motif to
 


    
 


    
 


    
 


    
 


 Mouse-over buttons for more information.

#### Data Formats

View the motif in
PSPM Format 


    
PSSM Format 


    
BLOCKS Format 


    
FASTA Format 


    
Raw Format 


    
or
Hide  
This feature requires javascript.

#### Sites

Click on any row to highlight sequence in all motifs.

| Name | Strand | Start | *p*-value | Sites | | |
| --- | --- | --- | --- | --- | --- | --- |
| chr10:11123760-11123860 | + | 23 | 3.51e-12 | GTGTGTGTAT | GTGTGTGTGTATGTGTGTG | TGCATATGTG |
| chr15:11429043-11429143 | + | 42 | 3.51e-12 | GTGTATGTGT | GTGTGTGTGTATGTGTGTG | TGTGTATGTG |
| chr13:25050499-25050599 | + | 76 | 7.22e-12 | TGCCGCGCGT | GTGTGTGTGTGTGTGTGTG | TGTGTG |
| chr1:136062109-136062209 | + | 54 | 1.05e-11 | GCGTGCATGT | GTGTGTATGTATGTGTGTG | TGTGGTGAGT |
| chr16:75291794-75291894 | + | 4 | 1.05e-11 | TGT | GTGTGTATGTATGTGTGTG | TGTGTATGTG |
| chr9:60539702-60539802 | - | 77 | 2.11e-11 | TATGT | GTGTGTGTGCATGTGTGTG | TGCGTGCGTG |
| chr15:101296982-101297082 | + | 49 | 2.11e-11 | ACGCATGCAC | GTGTGTGTGCATGTGTGTG | TGAGTGTGTA |
| chr11:33863308-33863408 | - | 5 | 4.61e-11 | GTGGGTGTGG | GTGTATATGTATGTGTGTG | TGGT |
| chr6:137792127-137792227 | - | 75 | 4.61e-11 | TGTGTGC | GTGCGTGTGTATGTGTGTG | TATGTGTGTG |
| chr5:146424152-146424252 | + | 70 | 4.61e-11 | GTATGTGCAT | GTGTGTATGCATGTGTGTG | CATGTGCACA |
| chr6:34325635-34325735 | + | 65 | 5.38e-11 | GTGTGCATGT | GTGTGCGTGTGTGTGTGTG | TGTGCATGTG |
| chr14:8006859-8006959 | - | 36 | 5.38e-11 | TGTGTGTGGT | GTGTGTGTGTGTGAGTGTG | TGTGTGTGTG |
| chr16:9738087-9738187 | + | 18 | 8.17e-11 | GTGCGCGTGT | GTGTGCATGTATGTGTGTG | TGTGTGCGTG |
| chr4:21829354-21829454 | + | 13 | 1.10e-10 | TTCTGTCTCT | GTGTGTCTGTGTGTGTGTG | TCTCTGTGTG |
| chr12:12370982-12371082 | + | 45 | 1.10e-10 | CTCTGTGTGT | GTGTGTCTGTGTGTGTGTG | TCTCTGTGTG |
| chr10:118908786-118908886 | + | 71 | 1.24e-10 | GTCTGTGTCT | GTGTGCATGTGTGTGTGTG | TGTGTCTGTG |
| chr2:172484480-172484580 | - | 4 | 1.49e-10 | GTCTGTGTAT | GTGTGTATGTATGTGTATG | TGT |
| chr3:130124484-130124584 | - | 37 | 1.89e-10 | GTGTGTGTGT | GTGCATGTGTATGTGTGTG | TGCACGTGTG |
| chr9:50683147-50683247 | - | 23 | 1.96e-10 | TGAGTGTGGT | GTGTGTGAGTGTGTGTGTG | TGAGAGTGTG |
| chr19:16767067-16767167 | - | 80 | 6.90e-10 | GT | GTGTGTCTGTGTGAGTGTG | TGTGAGTATG |
| chr9:45278995-45279095 | - | 62 | 6.90e-10 | GATTGTGTCT | GTGTGTCTGTGTGAGTGTG | TATGTATCTG |
| chr1:37036110-37036210 | + | 79 | 7.26e-10 | GTGCGGGTAT | GTGTGTGTGAGTGTGTGTG | TTT |
| chr8:44734948-44735048 | - | 71 | 9.40e-10 | TAGTGTGTGT | GTGAGTGTGCATGTGTGTG | TGTGCATGTG |
| chr14:79915824-79915924 | + | 38 | 1.27e-09 | GTGTTTGTGT | GTGTGCATGTATGTATGTG | TGAATACATG |
| chr4:154191336-154191436 | - | 19 | 1.55e-09 | TATATATGCT | GTATGTGTATATGTGTGTG | TGTAGTATGT |
| chr15:83991240-83991340 | - | 19 | 1.90e-09 | TGTGTGGTAT | GTGTGTGTGCATCTGTGTG | TGGTGTGTAT |
| chr1:154798318-154798418 | - | 49 | 2.12e-09 | TGAGCTAGGT | GTGTGCGTGCACGTGTGTG | CATGTGCGTG |
| chr5:120320367-120320467 | - | 76 | 2.28e-09 | GAGAGT | GTGTGCATATGTGTGTGTG | TGCATGCATG |
| chr7:29034256-29034356 | - | 51 | 2.28e-09 | GTGGTGTGGT | GTGTGTGTGTTGGTGTGTG | TGTTGGTGTG |
| chr8:126132997-126133097 | + | 52 | 3.79e-09 | GTGTATATGT | GTGTGCATGCATGTATGTG | TGAGTGTGTG |
| chr17:3307322-3307422 | - | 51 | 4.11e-09 | GTGTTTGTGA | GTGTACGTGTATGTTTGTG | TGAGTATATG |
| chr9:120355823-120355923 | + | 42 | 4.47e-09 | GTGTGACCAT | GTGCATATGTAGGTGTGTG | CATGTGTGAC |
| chr1:191198864-191198964 | + | 32 | 4.47e-09 | GTGTGTACGT | GTATGTATGCATGTGTATG | TGAGTGCATG |
| chr17:28383401-28383501 | + | 72 | 5.93e-09 | GTGACTGATT | GTGTATGTGTGTGACTGTG | TGTTTATGTG |
| chr5:97280498-97280598 | + | 65 | 7.18e-09 | TTGTGTGAGT | GTGTATGTGTGTGAGTGCG | TGTGACTGTG |
| chr17:29116579-29116679 | + | 42 | 7.76e-09 | GTGTGAGAGT | GTGTATGAGTGTATGTGTG | TTGAGCATGT |
| chr2:35609292-35609392 | - | 41 | 1.53e-08 | TCACACTCAC | GTGCATATGTATGGGTGTG | CATCCACAGG |
| chr11:109325648-109325748 | - | 76 | 1.67e-08 | GACTGT | GTGTGTGAGTACATGTGTG | TGTGAGGATG |
| chr4:108881520-108881620 | - | 81 | 1.67e-08 | T | GTGTATGGATGTGTGTGTG | GTGTGTGTGG |
| chr10:75570144-75570244 | + | 32 | 1.67e-08 | GTGTGTGCAT | GTGCACATGTGTGTGTATG | TTGTATGTGC |
| chr18:81664637-81664737 | + | 21 | 1.67e-08 | CTGTCTGTGT | GTGAGTGTGTTTGAGTGTG | TATGTGAGTA |
| chr4:129091654-129091754 | - | 40 | 2.49e-08 | ACAAGCGTGT | GTGTATATGTACTTGTGTG | TAGATGTATA |
| chr8:121438945-121439045 | - | 19 | 3.97e-08 | ATGTAAGTGT | GTATATGTTTATGAGTGTG | TGCTGTATGT |
| chr11:51555870-51555970 | + | 24 | 4.97e-08 | GTGTGTGTGT | GTGTGTGTGTGCAAGTATG | TTTTCTTCTA |
| chr11:98417533-98417633 | + | 74 | 6.66e-08 | TATGTGTGTT | GTGCCCATGTATGTATGTG | TGTAGGGG |
| chr13:38338228-38338328 | + | 48 | 1.43e-07 | GTGTCTGTCT | GTGTCTGTCTGTGTCTGTG | TCTGTGTCTA |
| chr10:94264745-94264845 | + | 48 | 1.97e-07 | TATGTGTGGT | GTGTTTGTGCATGTGCGCG | CGCTGTGTAT |
| chr17:8365907-8366007 | + | 62 | 3.07e-07 | ATGCGTGCAT | GTGCGTGTGCGTATGTATA | TGTGCATGTG |
| chr13:110070280-110070380 | + | 57 | 4.16e-07 | GTGTAAGTGT | GTGCGTGAGCGTGTAAGTG | TGTGAGTGAG |
| chr18:34164850-34164950 | - | 5 | 7.80e-07 | ACATGAGCAT | GTATGTGTGCAGGATCGTG | TACA |
| chr2:31449952-31450052 | + | 49 | 1.08e-06 | GTTGGCAGGT | GTATGTGGGCAGGTGCATG | CGTGCAGATG |

#### Block Diagrams

The height of the motif "block" is proportional to -log(p-value), truncated at the height for a motif with a p-value of 1e-10.  
Click on any row to highlight sequence in all motifs. Mouse over the center of the motif blocks to see more information.

| Name | Lowest *p*-value | Motif Location |
| --- | --- | --- |
| chr16:9738087-9738187 | 8.17e-11 | +  - |
| chr8:126132997-126133097 | 3.79e-09 | +  - |
| chr5:146424152-146424252 | 4.61e-11 | +  - |
| chr14:8006859-8006959 | 5.38e-11 | +  - |
| chr15:83991240-83991340 | 1.90e-09 | +  - |
| chr4:129091654-129091754 | 2.49e-08 | +  - |
| chr5:97280498-97280598 | 7.18e-09 | +  - |
| chr18:81664637-81664737 | 1.67e-08 | +  - |
| chr6:137792127-137792227 | 4.61e-11 | +  - |
| chr17:3307322-3307422 | 4.11e-09 | +  - |
| chr1:191198864-191198964 | 4.47e-09 | +  - |
| chr2:172484480-172484580 | 1.49e-10 | +  - |
| chr17:29116579-29116679 | 7.76e-09 | +  - |
| chr7:29034256-29034356 | 2.28e-09 | +  - |
| chr17:28383401-28383501 | 5.93e-09 | +  - |
| chr9:120355823-120355923 | 4.47e-09 | +  - |
| chr9:50683147-50683247 | 1.96e-10 | +  - |
| chr1:37036110-37036210 | 7.26e-10 | +  - |
| chr13:110070280-110070380 | 4.16e-07 | +  - |
| chr10:75570144-75570244 | 1.67e-08 | +  - |
| chr3:130124484-130124584 | 1.89e-10 | +  - |
| chr15:11429043-11429143 | 3.51e-12 | +  - |
| chr16:75291794-75291894 | 1.05e-11 | +  - |
| chr2:31449952-31450052 | 1.08e-06 | +  - |
| chr15:101296982-101297082 | 2.11e-11 | +  - |
| chr4:108881520-108881620 | 1.67e-08 | +  - |
| chr11:33863308-33863408 | 4.61e-11 | +  - |
| chr4:154191336-154191436 | 1.55e-09 | +  - |
| chr17:8365907-8366007 | 3.07e-07 | +  - |
| chr11:109325648-109325748 | 1.67e-08 | +  - |
| chr8:121438945-121439045 | 3.97e-08 | +  - |
| chr8:44734948-44735048 | 9.40e-10 | +  - |
| chr10:118908786-118908886 | 1.24e-10 | +  - |
| chr9:60539702-60539802 | 2.11e-11 | +  - |
| chr13:25050499-25050599 | 7.22e-12 | +  - |
| chr14:79915824-79915924 | 1.27e-09 | +  - |
| chr18:34164850-34164950 | 7.80e-07 | +  - |
| chr12:12370982-12371082 | 1.10e-10 | +  - |
| chr11:51555870-51555970 | 4.97e-08 | +  - |
| chr9:45278995-45279095 | 6.90e-10 | +  - |
| chr11:98417533-98417633 | 6.66e-08 | +  - |
| chr1:154798318-154798418 | 2.12e-09 | +  - |
| chr4:21829354-21829454 | 1.10e-10 | +  - |
| chr5:120320367-120320467 | 2.28e-09 | +  - |
| chr6:34325635-34325735 | 5.38e-11 | +  - |
| chr2:35609292-35609392 | 1.53e-08 | +  - |
| chr10:11123760-11123860 | 3.51e-12 | +  - |
| chr19:16767067-16767167 | 6.90e-10 | +  - |
| chr10:94264745-94264845 | 1.97e-07 | +  - |
| chr13:38338228-38338328 | 1.43e-07 | +  - |
| chr1:136062109-136062209 | 1.05e-11 | +  - |
|  | | 0      20      40      60      80      100 |

Time 1274.6 secs.

|  |  |
| --- | --- |
| All Motifs | Top |

#### Combined Block Diagrams

Non-overlapping sites with a *p*-value better than 0.0001.  
The height of the motif "block" is proportional to -log(p-value), truncated at the height for a motif with a p-value of 1e-10.  
Click on any row to highlight sequence in all motifs. The motif blocks have tool tips with more information.

|  |  |
| --- | --- |
|  | Motif 1 |

|  |  |
| --- | --- |
|  | Motif 2 |

|  |  |
| --- | --- |
|  | Motif 3 |

| Name | Combined *p*-value | Motif Location |
| --- | --- | --- |
| chr1:167273957-167274057 | 1.24e-01 | +  - |
| chr7:64058092-64058192 | 5.82e-02 | +  - |
| chr11:120393791-120393891 | 6.60e-03 | +  - |
| chr5:145152785-145152885 | 3.08e-02 | +  - |
| chr16:58497980-58498080 | 1.67e-01 | +  - |
| chr16:9738087-9738187 | 3.50e-12 | +  - |
| chr2:181055406-181055506 | 4.05e-03 | +  - |
| chr3:60546330-60546430 | 1.96e-02 | +  - |
| chr12:101084027-101084127 | 1.06e-01 | +  - |
| chr6:143833202-143833302 | 1.21e-01 | +  - |
| chr11:73047749-73047849 | 1.89e-02 | +  - |
| chr12:84161266-84161366 | 2.51e-02 | +  - |
| chr1:53784310-53784410 | 1.55e-02 | +  - |
| chr2:181458507-181458607 | 1.23e-02 | +  - |
| chr8:126132997-126133097 | 6.90e-13 | +  - |
| chr15:97844710-97844810 | 1.15e-02 | +  - |
| chr8:71960207-71960307 | 8.08e-04 | +  - |
| chr13:8871701-8871801 | 5.51e-03 | +  - |
| chr9:67013624-67013724 | 5.83e-02 | +  - |
| chr8:14890864-14890964 | 4.97e-02 | +  - |
| chr11:32269108-32269208 | 1.97e-02 | +  - |
| chr5:146424152-146424252 | 2.32e-11 | +  - |
| chr18:38350130-38350230 | 4.54e-02 | +  - |
| chr5:73354169-73354269 | 2.08e-02 | +  - |
| chr9:115518039-115518139 | 1.20e-01 | +  - |
| chr4:154541753-154541853 | 5.48e-02 | +  - |
| chr7:142087231-142087331 | 2.16e-06 | +  - |
| chr2:170128845-170128945 | 9.40e-02 | +  - |
| chr14:21823238-21823338 | 9.76e-03 | +  - |
| chr9:21817160-21817260 | 2.16e-02 | +  - |
| chr13:97453145-97453245 | 6.60e-02 | +  - |
| chr2:180059847-180059947 | 1.85e-03 | +  - |
| chr6:32534120-32534220 | 6.25e-02 | +  - |
| chr4:144981198-144981298 | 7.17e-02 | +  - |
| chr3:130810201-130810301 | 2.06e-02 | +  - |
| chr17:5099381-5099481 | 1.38e-03 | +  - |
| chr9:21384160-21384260 | 6.85e-03 | +  - |
| chr12:40036729-40036829 | 1.45e-03 | +  - |
| chr2:180325451-180325551 | 2.19e-02 | +  - |
| chr14:8006859-8006959 | 5.23e-13 | +  - |
| chr9:44343366-44343466 | 4.74e-02 | +  - |
| chr16:91865025-91865125 | 7.26e-02 | +  - |
| chr13:51976267-51976367 | 1.37e-04 | +  - |
| chr15:83991240-83991340 | 1.35e-13 | +  - |
| chr7:44483007-44483107 | 7.26e-10 | +  - |
| chr15:73015317-73015417 | 8.39e-04 | +  - |
| chr9:3328343-3328443 | 5.33e-04 | +  - |
| chr12:55329070-55329170 | 1.86e-03 | +  - |
| chr9:66347548-66347648 | 9.61e-05 | +  - |
| chr4:129091654-129091754 | 7.97e-09 | +  - |
| chr7:19632888-19632988 | 4.13e-04 | +  - |
| chr10:81352088-81352188 | 1.38e-01 | +  - |
| chr11:48838510-48838610 | 3.00e-02 | +  - |
| chr4:89510153-89510253 | 4.24e-02 | +  - |
| chr5:97280498-97280598 | 3.20e-12 | +  - |
| chr16:21357574-21357674 | 8.32e-02 | +  - |
| chr13:97066815-97066915 | 3.83e-02 | +  - |
| chr8:85526315-85526415 | 5.70e-02 | +  - |
| chr17:71681163-71681263 | 6.88e-02 | +  - |
| chr2:132695865-132695965 | 3.12e-03 | +  - |
| chr6:38831161-38831261 | 1.45e-04 | +  - |
| chr1:87712410-87712510 | 2.02e-01 | +  - |
| chr2:28323025-28323125 | 1.48e-02 | +  - |
| chr19:55865965-55866065 | 4.87e-06 | +  - |
| chr7:128672450-128672550 | 4.58e-04 | +  - |
| chr5:37456736-37456836 | 7.71e-02 | +  - |
| chr18:81664637-81664737 | 6.43e-13 | +  - |
| chr5:118489137-118489237 | 5.53e-03 | +  - |
| chr14:70107111-70107211 | 8.81e-03 | +  - |
| chr15:79741596-79741696 | 4.62e-02 | +  - |
| chr14:76520693-76520793 | 8.20e-02 | +  - |
| chr4:33450487-33450587 | 4.54e-03 | +  - |
| chr3:145590738-145590838 | 1.64e-04 | +  - |
| chr14:79533488-79533588 | 6.29e-03 | +  - |
| chr17:30347302-30347402 | 7.96e-02 | +  - |
| chr6:123293105-123293205 | 2.42e-02 | +  - |
| chr16:34031700-34031800 | 1.09e-01 | +  - |
| chr6:137792127-137792227 | 6.90e-13 | +  - |
| chr19:9899580-9899680 | 5.33e-03 | +  - |
| chr17:3307322-3307422 | 3.30e-10 | +  - |
| chr3:88325269-88325369 | 2.86e-02 | +  - |
| chr10:77210625-77210725 | 1.22e-04 | +  - |
| chr14:47116162-47116262 | 4.53e-03 | +  - |
| chr7:115831009-115831109 | 7.05e-03 | +  - |
| chr13:24681926-24682026 | 7.59e-02 | +  - |
| chr2:172298240-172298340 | 8.80e-02 | +  - |
| chr6:86364837-86364937 | 1.37e-02 | +  - |
| chr5:30166897-30166997 | 5.48e-02 | +  - |
| chr11:96075674-96075774 | 1.54e-01 | +  - |
| chr19:4878888-4878988 | 1.44e-01 | +  - |
| chr1:191198864-191198964 | 2.37e-13 | +  - |
| chr11:75234827-75234927 | 1.01e-02 | +  - |
| chr5:143819088-143819188 | 8.13e-03 | +  - |
| chr9:14988798-14988898 | 1.47e-04 | +  - |
| chr6:134984894-134984994 | 2.78e-03 | +  - |
| chr6:125104401-125104501 | 7.02e-02 | +  - |
| chr8:60900206-60900306 | 1.02e-02 | +  - |
| chr1:3435154-3435254 | 2.32e-02 | +  - |
| chr2:93443949-93444049 | 6.70e-02 | +  - |
| chr10:59875541-59875641 | 9.53e-02 | +  - |
| chr2:172484480-172484580 | 4.43e-12 | +  - |
| chr11:23313687-23313787 | 1.87e-02 | +  - |
| chr7:115988546-115988646 | 5.92e-02 | +  - |
| chr3:96082800-96082900 | 9.85e-03 | +  - |
| chr10:79975648-79975748 | 2.17e-03 | +  - |
| chr4:42880272-42880372 | 1.30e-01 | +  - |
| chr17:29116579-29116679 | 8.48e-11 | +  - |
| chr14:73322227-73322327 | 9.91e-03 | +  - |
| chr7:16412641-16412741 | 5.98e-03 | +  - |
| chr9:103288322-103288422 | 4.73e-02 | +  - |
| chr7:29034256-29034356 | 5.35e-11 | +  - |
| chr15:27497059-27497159 | 4.36e-02 | +  - |
| chr16:32541427-32541527 | 2.44e-03 | +  - |
| chr17:28383401-28383501 | 2.25e-13 | +  - |
| chr2:151011403-151011503 | 4.10e-02 | +  - |
| chr9:75547297-75547397 | 5.79e-02 | +  - |
| chr16:49771816-49771916 | 1.31e-01 | +  - |
| chr2:153525950-153526050 | 1.64e-01 | +  - |
| chr9:120355823-120355923 | 9.94e-10 | +  - |
| chr2:152741639-152741739 | 3.10e-02 | +  - |
| chr9:50683147-50683247 | 1.38e-11 | +  - |
| chr4:41026584-41026684 | 3.93e-03 | +  - |
| chr2:19024069-19024169 | 6.36e-03 | +  - |
| chr11:109557500-109557600 | 1.98e-02 | +  - |
| chr4:126803606-126803706 | 8.45e-02 | +  - |
| chr16:17220559-17220659 | 6.23e-02 | +  - |
| chr18:44335469-44335569 | 5.19e-02 | +  - |
| chr5:130029677-130029777 | 1.15e-01 | +  - |
| chr11:117451846-117451946 | 9.53e-03 | +  - |
| chr17:47598269-47598369 | 8.50e-02 | +  - |
| chr1:37036110-37036210 | 1.79e-10 | +  - |
| chr13:110070280-110070380 | 9.83e-09 | +  - |
| chr10:75570144-75570244 | 5.72e-11 | +  - |
| chr2:11212331-11212431 | 1.28e-01 | +  - |
| chr6:122394334-122394434 | 9.65e-03 | +  - |
| chr14:52306274-52306374 | 5.06e-02 | +  - |
| chr2:28418164-28418264 | 2.09e-02 | +  - |
| chr18:10092913-10093013 | 5.56e-03 | +  - |
| chr17:24628796-24628896 | 1.36e-01 | +  - |
| chr13:31909533-31909633 | 1.65e-02 | +  - |
| chr12:71164592-71164692 | 9.41e-03 | +  - |
| chr5:31733120-31733220 | 1.88e-02 | +  - |
| chr3:130124484-130124584 | 1.18e-12 | +  - |
| chr15:11429043-11429143 | 6.04e-14 | +  - |
| chr12:32143280-32143380 | 1.50e-01 | +  - |
| chr2:60223668-60223768 | 6.52e-03 | +  - |
| chr16:75291794-75291894 | 6.04e-14 | +  - |
| chr15:53202459-53202559 | 2.85e-03 | +  - |
| chr8:77688089-77688189 | 1.04e-05 | +  - |
| chr10:108582493-108582593 | 1.92e-02 | +  - |
| chr7:111004284-111004384 | 3.09e-04 | +  - |
| chr5:50126334-50126434 | 2.83e-02 | +  - |
| chr14:21549388-21549488 | 1.44e-02 | +  - |
| chr6:116350164-116350264 | 1.29e-01 | +  - |
| chr11:95835267-95835367 | 2.13e-01 | +  - |
| chr2:177305052-177305152 | 2.10e-08 | +  - |
| chr15:78268012-78268112 | 3.39e-03 | +  - |
| chr18:82568460-82568560 | 7.05e-02 | +  - |
| chr2:31449952-31450052 | 2.40e-06 | +  - |
| chr15:101296982-101297082 | 3.30e-13 | +  - |
| chr9:57070405-57070505 | 2.10e-02 | +  - |
| chr15:37100038-37100138 | 6.44e-02 | +  - |
| chr4:108881520-108881620 | 2.26e-11 | +  - |
| chr6:120174624-120174724 | 1.56e-01 | +  - |
| chr11:69258426-69258526 | 1.52e-03 | +  - |
| chr9:72902765-72902865 | 2.35e-02 | +  - |
| chr17:84926353-84926453 | 7.36e-02 | +  - |
| chr4:136653119-136653219 | 2.75e-03 | +  - |
| chr7:125472198-125472298 | 1.90e-02 | +  - |
| chr11:33863308-33863408 | 1.36e-12 | +  - |
| chr11:52115665-52115765 | 6.11e-02 | +  - |
| chr10:93679867-93679967 | 2.60e-03 | +  - |
| chr11:69589369-69589469 | 6.59e-03 | +  - |
| chr4:154191336-154191436 | 1.84e-11 | +  - |
| chr11:68884524-68884624 | 3.93e-03 | +  - |
| chr11:60486424-60486524 | 9.24e-02 | +  - |
| chr18:32556803-32556903 | 4.62e-02 | +  - |
| chr13:101573340-101573440 | 4.95e-02 | +  - |
| chr17:8365907-8366007 | 2.15e-07 | +  - |
| chr11:116615802-116615902 | 7.42e-03 | +  - |
| chr18:67246200-67246300 | 6.98e-03 | +  - |
| chr10:83060369-83060469 | 7.81e-02 | +  - |
| chr9:76236716-76236816 | 4.04e-03 | +  - |
| chr11:109325648-109325748 | 2.15e-09 | +  - |
| chr9:64779736-64779836 | 9.92e-02 | +  - |
| chr4:103120154-103120254 | 2.43e-02 | +  - |
| chr8:121438945-121439045 | 1.29e-11 | +  - |
| chr5:120599783-120599883 | 5.82e-03 | +  - |
| chr4:134663256-134663356 | 2.47e-04 | +  - |
| chr7:97589986-97590086 | 5.20e-02 | +  - |
| chr8:44734948-44735048 | 2.39e-13 | +  - |
| chr5:35035873-35035973 | 3.63e-02 | +  - |
| chr10:70133348-70133448 | 1.18e-02 | +  - |
| chr10:118908786-118908886 | 6.50e-13 | +  - |
| chr2:174297079-174297179 | 1.23e-02 | +  - |
| chr6:53134583-53134683 | 7.02e-02 | +  - |
| chr9:99242846-99242946 | 1.05e-02 | +  - |
| chr9:60539702-60539802 | 9.70e-13 | +  - |
| chr10:77232495-77232595 | 1.26e-01 | +  - |
| chr15:82342048-82342148 | 1.63e-03 | +  - |
| chr11:79008560-79008660 | 1.87e-02 | +  - |
| chr7:110920405-110920505 | 1.50e-02 | +  - |
| chr19:6284558-6284658 | 1.88e-03 | +  - |
| chr13:25050499-25050599 | 2.35e-13 | +  - |
| chr1:136014964-136015064 | 6.32e-02 | +  - |
| chr14:79915824-79915924 | 4.01e-11 | +  - |
| chrX:7837292-7837392 | 1.39e-02 | +  - |
| chr18:34164850-34164950 | 5.40e-08 | +  - |
| chr5:24927981-24928081 | 6.53e-02 | +  - |
| chr17:83489251-83489351 | 1.84e-01 | +  - |
| chr16:75305611-75305711 | 1.48e-03 | +  - |
| chr1:75450526-75450626 | 4.45e-02 | +  - |
| chr19:40977065-40977165 | 7.39e-02 | +  - |
| chr4:124645091-124645191 | 7.02e-02 | +  - |
| chr12:12370982-12371082 | 3.09e-12 | +  - |
| chr10:40328063-40328163 | 1.68e-02 | +  - |
| chr4:134924511-134924611 | 7.12e-02 | +  - |
| chr2:91664865-91664965 | 1.19e-05 | +  - |
| chr18:82634085-82634185 | 8.31e-02 | +  - |
| chr2:118660266-118660366 | 6.53e-02 | +  - |
| chr1:9782189-9782289 | 6.18e-02 | +  - |
| chr11:51555870-51555970 | 7.82e-14 | +  - |
| chr7:122015208-122015308 | 6.15e-03 | +  - |
| chr11:117308077-117308177 | 8.96e-02 | +  - |
| chr12:12427396-12427496 | 7.22e-02 | +  - |
| chr10:94073013-94073113 | 1.37e-02 | +  - |
| chr9:45278995-45279095 | 4.31e-11 | +  - |
| chr8:122296354-122296454 | 3.32e-02 | +  - |
| chr9:96254978-96255078 | 3.42e-02 | +  - |
| chr11:98417533-98417633 | 1.18e-12 | +  - |
| chr1:154798318-154798418 | 2.27e-13 | +  - |
| chr6:120580192-120580292 | 6.18e-03 | +  - |
| chr4:21829354-21829454 | 9.96e-12 | +  - |
| chr4:41129997-41130097 | 8.38e-09 | +  - |
| chr7:102958803-102958903 | 2.72e-03 | +  - |
| chr18:32024987-32025087 | 4.17e-03 | +  - |
| chr6:29583606-29583706 | 4.19e-02 | +  - |
| chr3:75918202-75918302 | 3.84e-04 | +  - |
| chr17:25598196-25598296 | 9.99e-03 | +  - |
| chr6:38757779-38757879 | 9.31e-03 | +  - |
| chr6:39012219-39012319 | 3.60e-05 | +  - |
| chr9:45803597-45803697 | 1.83e-01 | +  - |
| chr5:137116487-137116587 | 3.47e-02 | +  - |
| chr19:56543498-56543598 | 9.64e-02 | +  - |
| chr5:118317192-118317292 | 1.58e-08 | +  - |
| chr19:53277633-53277733 | 4.68e-03 | +  - |
| chr13:43191907-43192007 | 1.89e-02 | +  - |
| chr5:120320367-120320467 | 2.13e-11 | +  - |
| chr10:127126665-127126765 | 9.61e-03 | +  - |
| chr14:63067779-63067879 | 9.64e-02 | +  - |
| chr17:46679988-46680088 | 1.25e-03 | +  - |
| chr5:112238479-112238579 | 2.30e-02 | +  - |
| chr7:132492076-132492176 | 6.25e-04 | +  - |
| chr6:34325635-34325735 | 6.57e-13 | +  - |
| chr13:95965321-95965421 | 3.07e-03 | +  - |
| chr2:35609292-35609392 | 1.67e-07 | +  - |
| chr12:105019665-105019765 | 2.50e-02 | +  - |
| chr2:154634391-154634491 | 1.15e-01 | +  - |
| chr10:11123760-11123860 | 5.79e-14 | +  - |
| chr19:16767067-16767167 | 1.77e-10 | +  - |
| chr1:37479291-37479391 | 7.55e-05 | +  - |
| chr2:93390201-93390301 | 5.02e-05 | +  - |
| chr5:110813937-110814037 | 7.42e-03 | +  - |
| chr17:29658328-29658428 | 3.18e-02 | +  - |
| chr11:105366112-105366212 | 5.70e-03 | +  - |
| chr17:24527396-24527496 | 7.67e-02 | +  - |
| chr10:94264745-94264845 | 1.47e-07 | +  - |
| chr13:38338228-38338328 | 2.00e-07 | +  - |
| chr6:120530699-120530799 | 1.52e-05 | +  - |
| chr12:8609275-8609375 | 4.28e-02 | +  - |
| chr12:80930283-80930383 | 1.10e-02 | +  - |
| chr1:136062109-136062209 | 5.89e-14 | +  - |
|  | | 0      20      40      60      80      100 |

|  |  |
| --- | --- |
|  | Motif 1 |

|  |  |
| --- | --- |
|  | Motif 2 |

|  |  |
| --- | --- |
|  | Motif 3 |

Top

##### MEME version

4.9.1 (Release date: Fri Aug 23 16:49:42 2013 +1000)

##### Reference

Timothy L. Bailey and Charles Elkan,
"Fitting a mixture model by expectation maximization to discover motifs in biopolymers",
*Proceedings of the Second International Conference on Intelligent Systems
for Molecular Biology*, pp. 28-36, AAAI Press, Menlo Park, California, 1994.

show training set...

##### Training set:

Datafile = ./seqs-sampled  
Alphabet = ACGT  

| Sequence name | Weighting | Length |
| --- | --- | --- |
| chr1:167273957-167274057 | 1.000000 | 100 |
| chr15:84283240-84283340 | 1.000000 | 100 |
| chr11:120393791-120393891 | 1.000000 | 100 |
| chr16:58497980-58498080 | 1.000000 | 100 |
| chr11:61001628-61001728 | 1.000000 | 100 |
| chr11:85799595-85799695 | 1.000000 | 100 |
| chr6:143833202-143833302 | 1.000000 | 100 |
| chr12:84161266-84161366 | 1.000000 | 100 |
| chr8:126132997-126133097 | 1.000000 | 100 |
| chr3:94526911-94527011 | 1.000000 | 100 |
| chr5:34930300-34930400 | 1.000000 | 100 |
| chr1:85008761-85008861 | 1.000000 | 100 |
| chr11:32269108-32269208 | 1.000000 | 100 |
| chr4:120632026-120632126 | 1.000000 | 100 |
| chr15:64310703-64310803 | 1.000000 | 100 |
| chr5:73354169-73354269 | 1.000000 | 100 |
| chr15:75894350-75894450 | 1.000000 | 100 |
| chr11:86758157-86758257 | 1.000000 | 100 |
| chr17:5796947-5797047 | 1.000000 | 100 |
| chr14:21823238-21823338 | 1.000000 | 100 |
| chr2:131159566-131159666 | 1.000000 | 100 |
| chr2:37771930-37772030 | 1.000000 | 100 |
| chr14:47515830-47515930 | 1.000000 | 100 |
| chr4:144981198-144981298 | 1.000000 | 100 |
| chr2:60163894-60163994 | 1.000000 | 100 |
| chr9:21384160-21384260 | 1.000000 | 100 |
| chr10:81271488-81271588 | 1.000000 | 100 |
| chr9:58662297-58662397 | 1.000000 | 100 |
| chr15:99321390-99321490 | 1.000000 | 100 |
| chr4:119198681-119198781 | 1.000000 | 100 |
| chr13:51976267-51976367 | 1.000000 | 100 |
| chr15:83991240-83991340 | 1.000000 | 100 |
| chr9:3328343-3328443 | 1.000000 | 100 |
| chr17:27774012-27774112 | 1.000000 | 100 |
| chr9:66347548-66347648 | 1.000000 | 100 |
| chr9:113496284-113496384 | 1.000000 | 100 |
| chr4:129091654-129091754 | 1.000000 | 100 |
| chr10:81352088-81352188 | 1.000000 | 100 |
| chr11:48838510-48838610 | 1.000000 | 100 |
| chr4:89510153-89510253 | 1.000000 | 100 |
| chr14:70469768-70469868 | 1.000000 | 100 |
| chrX:152307529-152307629 | 1.000000 | 100 |
| chr8:85526315-85526415 | 1.000000 | 100 |
| chr18:35619702-35619802 | 1.000000 | 100 |
| chr3:51387110-51387210 | 1.000000 | 100 |
| chr5:76161651-76161751 | 1.000000 | 100 |
| chr13:57659949-57660049 | 1.000000 | 100 |
| chr11:11835494-11835594 | 1.000000 | 100 |
| chr17:47210549-47210649 | 1.000000 | 100 |
| chr17:79926012-79926112 | 1.000000 | 100 |
| chr5:118489137-118489237 | 1.000000 | 100 |
| chr4:46075811-46075911 | 1.000000 | 100 |
| chr13:23682509-23682609 | 1.000000 | 100 |
| chr2:151625496-151625596 | 1.000000 | 100 |
| chr11:61067579-61067679 | 1.000000 | 100 |
| chr2:121140435-121140535 | 1.000000 | 100 |
| chr17:30347302-30347402 | 1.000000 | 100 |
| chr6:137792127-137792227 | 1.000000 | 100 |
| chr14:57548974-57549074 | 1.000000 | 100 |
| chr1:9783760-9783860 | 1.000000 | 100 |
| chr3:88325269-88325369 | 1.000000 | 100 |
| chr14:47116162-47116262 | 1.000000 | 100 |
| chr17:49909548-49909648 | 1.000000 | 100 |
| chr2:130667691-130667791 | 1.000000 | 100 |
| chr2:172298240-172298340 | 1.000000 | 100 |
| chr13:73443549-73443649 | 1.000000 | 100 |
| chr13:111548477-111548577 | 1.000000 | 100 |
| chr5:30166897-30166997 | 1.000000 | 100 |
| chr11:96075674-96075774 | 1.000000 | 100 |
| chr15:78389414-78389514 | 1.000000 | 100 |
| chr9:35570288-35570388 | 1.000000 | 100 |
| chr2:4573053-4573153 | 1.000000 | 100 |
| chr2:166665721-166665821 | 1.000000 | 100 |
| chr6:125104401-125104501 | 1.000000 | 100 |
| chr9:59656629-59656729 | 1.000000 | 100 |
| chr4:147944089-147944189 | 1.000000 | 100 |
| chr10:59875541-59875641 | 1.000000 | 100 |
| chr7:141131063-141131163 | 1.000000 | 100 |
| chr18:89176843-89176943 | 1.000000 | 100 |
| chr10:79975648-79975748 | 1.000000 | 100 |
| chr17:29116579-29116679 | 1.000000 | 100 |
| chr1:151755195-151755295 | 1.000000 | 100 |
| chr9:103288322-103288422 | 1.000000 | 100 |
| chr15:27497059-27497159 | 1.000000 | 100 |
| chr19:10014976-10015076 | 1.000000 | 100 |
| chr12:71904407-71904507 | 1.000000 | 100 |
| chr16:49771816-49771916 | 1.000000 | 100 |
| chr9:120355823-120355923 | 1.000000 | 100 |
| chr2:152741639-152741739 | 1.000000 | 100 |
| chr4:41026584-41026684 | 1.000000 | 100 |
| chr16:91729254-91729354 | 1.000000 | 100 |
| chr11:109557500-109557600 | 1.000000 | 100 |
| chr1:181260241-181260341 | 1.000000 | 100 |
| chr16:17220559-17220659 | 1.000000 | 100 |
| chr18:44335469-44335569 | 1.000000 | 100 |
| chr5:130029677-130029777 | 1.000000 | 100 |
| chr1:135855740-135855840 | 1.000000 | 100 |
| chr1:37036110-37036210 | 1.000000 | 100 |
| chr10:75570144-75570244 | 1.000000 | 100 |
| chr7:27454859-27454959 | 1.000000 | 100 |
| chr1:106637834-106637934 | 1.000000 | 100 |
| chr11:85298177-85298277 | 1.000000 | 100 |
| chr17:24628796-24628896 | 1.000000 | 100 |
| chr12:71164592-71164692 | 1.000000 | 100 |
| chr8:46551208-46551308 | 1.000000 | 100 |
| chr3:130124484-130124584 | 1.000000 | 100 |
| chr14:54426739-54426839 | 1.000000 | 100 |
| chr16:75291794-75291894 | 1.000000 | 100 |
| chr8:77688089-77688189 | 1.000000 | 100 |
| chr7:111004284-111004384 | 1.000000 | 100 |
| chr5:50126334-50126434 | 1.000000 | 100 |
| chr11:80376292-80376392 | 1.000000 | 100 |
| chr2:177305052-177305152 | 1.000000 | 100 |
| chr4:152257240-152257340 | 1.000000 | 100 |
| chr2:164469044-164469144 | 1.000000 | 100 |
| chr6:34876128-34876228 | 1.000000 | 100 |
| chr15:37100038-37100138 | 1.000000 | 100 |
| chr1:181257361-181257461 | 1.000000 | 100 |
| chr6:120174624-120174724 | 1.000000 | 100 |
| chr11:69258426-69258526 | 1.000000 | 100 |
| chr17:25124644-25124744 | 1.000000 | 100 |
| chr9:72902765-72902865 | 1.000000 | 100 |
| chr9:108339161-108339261 | 1.000000 | 100 |
| chr7:125472198-125472298 | 1.000000 | 100 |
| chr11:52115665-52115765 | 1.000000 | 100 |
| chr4:147985931-147986031 | 1.000000 | 100 |
| chr11:68884524-68884624 | 1.000000 | 100 |
| chr1:14484685-14484785 | 1.000000 | 100 |
| chr7:49327048-49327148 | 1.000000 | 100 |
| chr13:101573340-101573440 | 1.000000 | 100 |
| chr11:116615802-116615902 | 1.000000 | 100 |
| chr9:76236716-76236816 | 1.000000 | 100 |
| chr9:64779736-64779836 | 1.000000 | 100 |
| chr17:24209033-24209133 | 1.000000 | 100 |
| chr6:43507104-43507204 | 1.000000 | 100 |
| chr4:134663256-134663356 | 1.000000 | 100 |
| chr1:135941758-135941858 | 1.000000 | 100 |
| chr5:35035873-35035973 | 1.000000 | 100 |
| chr10:118908786-118908886 | 1.000000 | 100 |
| chr2:174297079-174297179 | 1.000000 | 100 |
| chr6:53134583-53134683 | 1.000000 | 100 |
| chr4:132530565-132530665 | 1.000000 | 100 |
| chr10:77232495-77232595 | 1.000000 | 100 |
| chr15:82342048-82342148 | 1.000000 | 100 |
| chr7:110920405-110920505 | 1.000000 | 100 |
| chr13:25050499-25050599 | 1.000000 | 100 |
| chr14:79915824-79915924 | 1.000000 | 100 |
| chr5:24927981-24928081 | 1.000000 | 100 |
| chr17:83489251-83489351 | 1.000000 | 100 |
| chr16:75305611-75305711 | 1.000000 | 100 |
| chr3:55080176-55080276 | 1.000000 | 100 |
| chr12:12370982-12371082 | 1.000000 | 100 |
| chr1:144006685-144006785 | 1.000000 | 100 |
| chr18:82634085-82634185 | 1.000000 | 100 |
| chr10:80708478-80708578 | 1.000000 | 100 |
| chr18:82471949-82472049 | 1.000000 | 100 |
| chr13:34312449-34312549 | 1.000000 | 100 |
| chr11:117308077-117308177 | 1.000000 | 100 |
| chr6:60828133-60828233 | 1.000000 | 100 |
| chr10:94073013-94073113 | 1.000000 | 100 |
| chr8:122296354-122296454 | 1.000000 | 100 |
| chr11:78658035-78658135 | 1.000000 | 100 |
| chr19:24529077-24529177 | 1.000000 | 100 |
| chr8:20545738-20545838 | 1.000000 | 100 |
| chr1:154798318-154798418 | 1.000000 | 100 |
| chr4:21829354-21829454 | 1.000000 | 100 |
| chr7:102958803-102958903 | 1.000000 | 100 |
| chr8:57487238-57487338 | 1.000000 | 100 |
| chr14:21052193-21052293 | 1.000000 | 100 |
| chr14:6157918-6158018 | 1.000000 | 100 |
| chr3:75918202-75918302 | 1.000000 | 100 |
| chr15:38301922-38302022 | 1.000000 | 100 |
| chr7:44748353-44748453 | 1.000000 | 100 |
| chr6:39012219-39012319 | 1.000000 | 100 |
| chr11:60914061-60914161 | 1.000000 | 100 |
| chr5:118317192-118317292 | 1.000000 | 100 |
| chr5:120320367-120320467 | 1.000000 | 100 |
| chr6:58640791-58640891 | 1.000000 | 100 |
| chr14:63067779-63067879 | 1.000000 | 100 |
| chr10:85957586-85957686 | 1.000000 | 100 |
| chr5:112238479-112238579 | 1.000000 | 100 |
| chr6:34325635-34325735 | 1.000000 | 100 |
| chr6:5287482-5287582 | 1.000000 | 100 |
| chr6:88007490-88007590 | 1.000000 | 100 |
| chr10:119095650-119095750 | 1.000000 | 100 |
| chr5:100518126-100518226 | 1.000000 | 100 |
| chr9:54610706-54610806 | 1.000000 | 100 |
| chr13:12975066-12975166 | 1.000000 | 100 |
| chr5:134593271-134593371 | 1.000000 | 100 |
| chr9:72654983-72655083 | 1.000000 | 100 |
| chr2:93390201-93390301 | 1.000000 | 100 |
| chr17:29658328-29658428 | 1.000000 | 100 |
| chr17:20945519-20945619 | 1.000000 | 100 |
| chr11:105366112-105366212 | 1.000000 | 100 |
| chr14:66266397-66266497 | 1.000000 | 100 |
| chr13:38338228-38338328 | 1.000000 | 100 |
| chr6:38676679-38676779 | 1.000000 | 100 |
| chr19:34588411-34588511 | 1.000000 | 100 |
| chr7:75834765-75834865 | 1.000000 | 100 |
| chr12:8609275-8609375 | 1.000000 | 100 |

| Sequence name | Weighting | Length |
| --- | --- | --- |
| chr18:88754956-88755056 | 1.000000 | 100 |
| chr2:167732977-167733077 | 1.000000 | 100 |
| chrX:8102334-8102434 | 1.000000 | 100 |
| chr16:9738087-9738187 | 1.000000 | 100 |
| chr10:41912482-41912582 | 1.000000 | 100 |
| chr3:60546330-60546430 | 1.000000 | 100 |
| chr11:73047749-73047849 | 1.000000 | 100 |
| chr1:53784310-53784410 | 1.000000 | 100 |
| chr15:97844710-97844810 | 1.000000 | 100 |
| chr17:27442535-27442635 | 1.000000 | 100 |
| chr13:8871701-8871801 | 1.000000 | 100 |
| chr11:17211560-17211660 | 1.000000 | 100 |
| chr5:146424152-146424252 | 1.000000 | 100 |
| chr17:86162707-86162807 | 1.000000 | 100 |
| chr18:38350130-38350230 | 1.000000 | 100 |
| chr6:72335852-72335952 | 1.000000 | 100 |
| chr9:115518039-115518139 | 1.000000 | 100 |
| chr7:142087231-142087331 | 1.000000 | 100 |
| chr2:170128845-170128945 | 1.000000 | 100 |
| chr9:21817160-21817260 | 1.000000 | 100 |
| chr4:123718285-123718385 | 1.000000 | 100 |
| chr12:76710036-76710136 | 1.000000 | 100 |
| chr5:90224037-90224137 | 1.000000 | 100 |
| chr3:130810201-130810301 | 1.000000 | 100 |
| chr2:155989250-155989350 | 1.000000 | 100 |
| chr12:40036729-40036829 | 1.000000 | 100 |
| chr2:180325451-180325551 | 1.000000 | 100 |
| chr13:23571237-23571337 | 1.000000 | 100 |
| chr3:79580337-79580437 | 1.000000 | 100 |
| chr8:83934862-83934962 | 1.000000 | 100 |
| chr2:18973841-18973941 | 1.000000 | 100 |
| chr7:44483007-44483107 | 1.000000 | 100 |
| chr3:95742334-95742434 | 1.000000 | 100 |
| chr12:55329070-55329170 | 1.000000 | 100 |
| chr4:129189908-129190008 | 1.000000 | 100 |
| chr16:21858712-21858812 | 1.000000 | 100 |
| chr7:19632888-19632988 | 1.000000 | 100 |
| chr12:3284025-3284125 | 1.000000 | 100 |
| chrX:170856865-170856965 | 1.000000 | 100 |
| chr10:71238009-71238109 | 1.000000 | 100 |
| chr5:97280498-97280598 | 1.000000 | 100 |
| chr11:82847768-82847868 | 1.000000 | 100 |
| chr5:113973863-113973963 | 1.000000 | 100 |
| chr14:13138827-13138927 | 1.000000 | 100 |
| chr2:132695865-132695965 | 1.000000 | 100 |
| chr6:38831161-38831261 | 1.000000 | 100 |
| chr2:28323025-28323125 | 1.000000 | 100 |
| chr2:32085363-32085463 | 1.000000 | 100 |
| chr5:37456736-37456836 | 1.000000 | 100 |
| chr18:81664637-81664737 | 1.000000 | 100 |
| chr14:70107111-70107211 | 1.000000 | 100 |
| chr15:79741596-79741696 | 1.000000 | 100 |
| chr16:33941768-33941868 | 1.000000 | 100 |
| chr14:76520693-76520793 | 1.000000 | 100 |
| chr11:117810749-117810849 | 1.000000 | 100 |
| chr3:145590738-145590838 | 1.000000 | 100 |
| chr6:123293105-123293205 | 1.000000 | 100 |
| chr19:9899580-9899680 | 1.000000 | 100 |
| chr6:38341809-38341909 | 1.000000 | 100 |
| chr16:87704585-87704685 | 1.000000 | 100 |
| chrX:7971408-7971508 | 1.000000 | 100 |
| chr9:121837292-121837392 | 1.000000 | 100 |
| chr6:114981846-114981946 | 1.000000 | 100 |
| chr2:94178786-94178886 | 1.000000 | 100 |
| chr19:32222270-32222370 | 1.000000 | 100 |
| chr6:86364837-86364937 | 1.000000 | 100 |
| chr15:101202481-101202581 | 1.000000 | 100 |
| chr3:122113598-122113698 | 1.000000 | 100 |
| chr19:4878888-4878988 | 1.000000 | 100 |
| chr11:75234827-75234927 | 1.000000 | 100 |
| chr1:37429795-37429895 | 1.000000 | 100 |
| chr6:134984894-134984994 | 1.000000 | 100 |
| chr8:122330606-122330706 | 1.000000 | 100 |
| chr4:131564348-131564448 | 1.000000 | 100 |
| chr8:60900206-60900306 | 1.000000 | 100 |
| chr1:3435154-3435254 | 1.000000 | 100 |
| chr2:172484480-172484580 | 1.000000 | 100 |
| chr11:23313687-23313787 | 1.000000 | 100 |
| chr18:82615522-82615622 | 1.000000 | 100 |
| chr4:42880272-42880372 | 1.000000 | 100 |
| chr17:46646448-46646548 | 1.000000 | 100 |
| chr7:16412641-16412741 | 1.000000 | 100 |
| chr12:3773626-3773726 | 1.000000 | 100 |
| chr16:32541427-32541527 | 1.000000 | 100 |
| chr2:151011403-151011503 | 1.000000 | 100 |
| chr9:75547297-75547397 | 1.000000 | 100 |
| chr2:153525950-153526050 | 1.000000 | 100 |
| chr8:20507123-20507223 | 1.000000 | 100 |
| chr10:77163907-77164007 | 1.000000 | 100 |
| chr5:100798575-100798675 | 1.000000 | 100 |
| chr2:19024069-19024169 | 1.000000 | 100 |
| chr8:68861982-68862082 | 1.000000 | 100 |
| chr4:126803606-126803706 | 1.000000 | 100 |
| chr4:149152565-149152665 | 1.000000 | 100 |
| chr11:44509017-44509117 | 1.000000 | 100 |
| chr11:117451846-117451946 | 1.000000 | 100 |
| chr17:47598269-47598369 | 1.000000 | 100 |
| chr13:110070280-110070380 | 1.000000 | 100 |
| chr2:11212331-11212431 | 1.000000 | 100 |
| chr6:122394334-122394434 | 1.000000 | 100 |
| chr14:52306274-52306374 | 1.000000 | 100 |
| chr18:10092913-10093013 | 1.000000 | 100 |
| chr13:31909533-31909633 | 1.000000 | 100 |
| chr2:119163310-119163410 | 1.000000 | 100 |
| chr2:75455213-75455313 | 1.000000 | 100 |
| chr15:11429043-11429143 | 1.000000 | 100 |
| chr4:130112948-130113048 | 1.000000 | 100 |
| chr15:53202459-53202559 | 1.000000 | 100 |
| chr10:108582493-108582593 | 1.000000 | 100 |
| chr5:136604935-136605035 | 1.000000 | 100 |
| chr4:62373277-62373377 | 1.000000 | 100 |
| chr6:116350164-116350264 | 1.000000 | 100 |
| chr7:65640007-65640107 | 1.000000 | 100 |
| chr2:132076337-132076437 | 1.000000 | 100 |
| chr2:31449952-31450052 | 1.000000 | 100 |
| chr9:57070405-57070505 | 1.000000 | 100 |
| chr4:108881520-108881620 | 1.000000 | 100 |
| chr15:80130822-80130922 | 1.000000 | 100 |
| chr17:79365207-79365307 | 1.000000 | 100 |
| chr9:66895075-66895175 | 1.000000 | 100 |
| chr7:30169816-30169916 | 1.000000 | 100 |
| chr15:73130772-73130872 | 1.000000 | 100 |
| chr7:144605681-144605781 | 1.000000 | 100 |
| chr6:28710210-28710310 | 1.000000 | 100 |
| chr10:93679867-93679967 | 1.000000 | 100 |
| chr11:69589369-69589469 | 1.000000 | 100 |
| chr11:118562301-118562401 | 1.000000 | 100 |
| chr4:135903912-135904012 | 1.000000 | 100 |
| chr18:32556803-32556903 | 1.000000 | 100 |
| chr17:8365907-8366007 | 1.000000 | 100 |
| chr18:67246200-67246300 | 1.000000 | 100 |
| chr11:109325648-109325748 | 1.000000 | 100 |
| chr4:103120154-103120254 | 1.000000 | 100 |
| chr2:153608293-153608393 | 1.000000 | 100 |
| chr5:120599783-120599883 | 1.000000 | 100 |
| chr7:97589986-97590086 | 1.000000 | 100 |
| chr8:44734948-44735048 | 1.000000 | 100 |
| chr12:75427489-75427589 | 1.000000 | 100 |
| chr2:57629578-57629678 | 1.000000 | 100 |
| chr11:103216680-103216780 | 1.000000 | 100 |
| chr9:99242846-99242946 | 1.000000 | 100 |
| chr9:60539702-60539802 | 1.000000 | 100 |
| chr18:49981910-49982010 | 1.000000 | 100 |
| chr11:79008560-79008660 | 1.000000 | 100 |
| chr7:128450470-128450570 | 1.000000 | 100 |
| chr1:136014964-136015064 | 1.000000 | 100 |
| chrX:7837292-7837392 | 1.000000 | 100 |
| chr4:118225591-118225691 | 1.000000 | 100 |
| chrX:31176226-31176326 | 1.000000 | 100 |
| chr1:75450526-75450626 | 1.000000 | 100 |
| chr4:124645091-124645191 | 1.000000 | 100 |
| chr10:40328063-40328163 | 1.000000 | 100 |
| chr19:44997734-44997834 | 1.000000 | 100 |
| chr6:91786509-91786609 | 1.000000 | 100 |
| chr1:9782189-9782289 | 1.000000 | 100 |
| chr11:51555870-51555970 | 1.000000 | 100 |
| chr2:32236440-32236540 | 1.000000 | 100 |
| chr12:12427396-12427496 | 1.000000 | 100 |
| chr17:57290458-57290558 | 1.000000 | 100 |
| chr9:45278995-45279095 | 1.000000 | 100 |
| chr4:29124150-29124250 | 1.000000 | 100 |
| chr2:79259440-79259540 | 1.000000 | 100 |
| chr11:98417533-98417633 | 1.000000 | 100 |
| chr4:133056800-133056900 | 1.000000 | 100 |
| chr7:142996117-142996217 | 1.000000 | 100 |
| chr11:90723412-90723512 | 1.000000 | 100 |
| chr7:143812009-143812109 | 1.000000 | 100 |
| chr15:36727889-36727989 | 1.000000 | 100 |
| chr18:32024987-32025087 | 1.000000 | 100 |
| chr6:119361106-119361206 | 1.000000 | 100 |
| chr2:152847820-152847920 | 1.000000 | 100 |
| chr7:16819975-16820075 | 1.000000 | 100 |
| chr13:103642919-103643019 | 1.000000 | 100 |
| chr15:64381366-64381466 | 1.000000 | 100 |
| chr5:137116487-137116587 | 1.000000 | 100 |
| chr19:53277633-53277733 | 1.000000 | 100 |
| chr10:127290776-127290876 | 1.000000 | 100 |
| chr10:86021753-86021853 | 1.000000 | 100 |
| chr18:3115677-3115777 | 1.000000 | 100 |
| chr2:120969738-120969838 | 1.000000 | 100 |
| chr18:68339354-68339454 | 1.000000 | 100 |
| chr2:32950894-32950994 | 1.000000 | 100 |
| chr2:32063862-32063962 | 1.000000 | 100 |
| chr5:118480125-118480225 | 1.000000 | 100 |
| chr2:35609292-35609392 | 1.000000 | 100 |
| chr8:10995804-10995904 | 1.000000 | 100 |
| chr12:105019665-105019765 | 1.000000 | 100 |
| chr2:154634391-154634491 | 1.000000 | 100 |
| chr10:11123760-11123860 | 1.000000 | 100 |
| chr1:37479291-37479391 | 1.000000 | 100 |
| chr2:23572421-23572521 | 1.000000 | 100 |
| chr10:80611339-80611439 | 1.000000 | 100 |
| chr14:25525093-25525193 | 1.000000 | 100 |
| chr17:24527396-24527496 | 1.000000 | 100 |
| chr6:114309478-114309578 | 1.000000 | 100 |
| chr6:120530699-120530799 | 1.000000 | 100 |
| chr1:157695418-157695518 | 1.000000 | 100 |
| chr1:4857707-4857807 | 1.000000 | 100 |
| chr18:38299566-38299666 | 1.000000 | 100 |
| chr12:80930283-80930383 | 1.000000 | 100 |

| Sequence name | Weighting | Length |
| --- | --- | --- |
| chr7:64058092-64058192 | 1.000000 | 100 |
| chr1:23533446-23533546 | 1.000000 | 100 |
| chr5:145152785-145152885 | 1.000000 | 100 |
| chr2:181055406-181055506 | 1.000000 | 100 |
| chr9:57563083-57563183 | 1.000000 | 100 |
| chr12:101084027-101084127 | 1.000000 | 100 |
| chr4:40849690-40849790 | 1.000000 | 100 |
| chr2:181458507-181458607 | 1.000000 | 100 |
| chr8:35589232-35589332 | 1.000000 | 100 |
| chr8:71960207-71960307 | 1.000000 | 100 |
| chr9:67013624-67013724 | 1.000000 | 100 |
| chr8:14890864-14890964 | 1.000000 | 100 |
| chr14:75834376-75834476 | 1.000000 | 100 |
| chr2:28840014-28840114 | 1.000000 | 100 |
| chr4:133910995-133911095 | 1.000000 | 100 |
| chr7:16717928-16718028 | 1.000000 | 100 |
| chr4:154541753-154541853 | 1.000000 | 100 |
| chr5:36786366-36786466 | 1.000000 | 100 |
| chr19:44396904-44397004 | 1.000000 | 100 |
| chr13:97453145-97453245 | 1.000000 | 100 |
| chr2:180059847-180059947 | 1.000000 | 100 |
| chr10:93124454-93124554 | 1.000000 | 100 |
| chr6:32534120-32534220 | 1.000000 | 100 |
| chr17:5099381-5099481 | 1.000000 | 100 |
| chr4:133680466-133680566 | 1.000000 | 100 |
| chr3:95282555-95282655 | 1.000000 | 100 |
| chr14:8006859-8006959 | 1.000000 | 100 |
| chr6:128072555-128072655 | 1.000000 | 100 |
| chr9:44343366-44343466 | 1.000000 | 100 |
| chr16:91865025-91865125 | 1.000000 | 100 |
| chr8:105901119-105901219 | 1.000000 | 100 |
| chr15:73015317-73015417 | 1.000000 | 100 |
| chr9:122104015-122104115 | 1.000000 | 100 |
| chr2:151913759-151913859 | 1.000000 | 100 |
| chr2:179750705-179750805 | 1.000000 | 100 |
| chrX:159597637-159597737 | 1.000000 | 100 |
| chr1:36547021-36547121 | 1.000000 | 100 |
| chr12:100958677-100958777 | 1.000000 | 100 |
| chr7:122970998-122971098 | 1.000000 | 100 |
| chr7:100860365-100860465 | 1.000000 | 100 |
| chr16:21357574-21357674 | 1.000000 | 100 |
| chr13:97066815-97066915 | 1.000000 | 100 |
| chr8:23249330-23249430 | 1.000000 | 100 |
| chr17:71681163-71681263 | 1.000000 | 100 |
| chr9:113916014-113916114 | 1.000000 | 100 |
| chr1:87712410-87712510 | 1.000000 | 100 |
| chr19:55865965-55866065 | 1.000000 | 100 |
| chr7:128672450-128672550 | 1.000000 | 100 |
| chr13:20150271-20150371 | 1.000000 | 100 |
| chr15:11399857-11399957 | 1.000000 | 100 |
| chr4:154773498-154773598 | 1.000000 | 100 |
| chr10:86026493-86026593 | 1.000000 | 100 |
| chr9:21924184-21924284 | 1.000000 | 100 |
| chr4:128760194-128760294 | 1.000000 | 100 |
| chr4:33450487-33450587 | 1.000000 | 100 |
| chr14:79533488-79533588 | 1.000000 | 100 |
| chr16:34031700-34031800 | 1.000000 | 100 |
| chr1:82816070-82816170 | 1.000000 | 100 |
| chr17:3307322-3307422 | 1.000000 | 100 |
| chr11:4659978-4660078 | 1.000000 | 100 |
| chr10:77210625-77210725 | 1.000000 | 100 |
| chr7:115831009-115831109 | 1.000000 | 100 |
| chr16:29839311-29839411 | 1.000000 | 100 |
| chr13:24681926-24682026 | 1.000000 | 100 |
| chr8:94964944-94965044 | 1.000000 | 100 |
| chr16:90197429-90197529 | 1.000000 | 100 |
| chr6:87851062-87851162 | 1.000000 | 100 |
| chr5:140749060-140749160 | 1.000000 | 100 |
| chr1:191198864-191198964 | 1.000000 | 100 |
| chr5:143819088-143819188 | 1.000000 | 100 |
| chr9:14988798-14988898 | 1.000000 | 100 |
| chr2:170151668-170151768 | 1.000000 | 100 |
| chr18:61960695-61960795 | 1.000000 | 100 |
| chr16:91044511-91044611 | 1.000000 | 100 |
| chr14:118913743-118913843 | 1.000000 | 100 |
| chr2:93443949-93444049 | 1.000000 | 100 |
| chr11:4096731-4096831 | 1.000000 | 100 |
| chr7:115988546-115988646 | 1.000000 | 100 |
| chr3:96082800-96082900 | 1.000000 | 100 |
| chr5:21051917-21052017 | 1.000000 | 100 |
| chr14:73322227-73322327 | 1.000000 | 100 |
| chr1:166138131-166138231 | 1.000000 | 100 |
| chr7:29034256-29034356 | 1.000000 | 100 |
| chr17:28383401-28383501 | 1.000000 | 100 |
| chr2:92397134-92397234 | 1.000000 | 100 |
| chr8:117089958-117090058 | 1.000000 | 100 |
| chr7:16783591-16783691 | 1.000000 | 100 |
| chr8:36589368-36589468 | 1.000000 | 100 |
| chr9:50683147-50683247 | 1.000000 | 100 |
| chr9:59271788-59271888 | 1.000000 | 100 |
| chr3:37546502-37546602 | 1.000000 | 100 |
| chr1:154841797-154841897 | 1.000000 | 100 |
| chr3:37624816-37624916 | 1.000000 | 100 |
| chr5:134768756-134768856 | 1.000000 | 100 |
| chr18:77811773-77811873 | 1.000000 | 100 |
| chr14:79530302-79530402 | 1.000000 | 100 |
| chr11:6267729-6267829 | 1.000000 | 100 |
| chr8:105881660-105881760 | 1.000000 | 100 |
| chr18:80296274-80296374 | 1.000000 | 100 |
| chr1:133133546-133133646 | 1.000000 | 100 |
| chr2:28418164-28418264 | 1.000000 | 100 |
| chr13:83847667-83847767 | 1.000000 | 100 |
| chr2:166657029-166657129 | 1.000000 | 100 |
| chr1:159238253-159238353 | 1.000000 | 100 |
| chr5:31733120-31733220 | 1.000000 | 100 |
| chr12:32143280-32143380 | 1.000000 | 100 |
| chr2:60223668-60223768 | 1.000000 | 100 |
| chr3:145691018-145691118 | 1.000000 | 100 |
| chr13:35677724-35677824 | 1.000000 | 100 |
| chr4:140816232-140816332 | 1.000000 | 100 |
| chr14:21549388-21549488 | 1.000000 | 100 |
| chr11:95835267-95835367 | 1.000000 | 100 |
| chr15:78268012-78268112 | 1.000000 | 100 |
| chr18:82568460-82568560 | 1.000000 | 100 |
| chr15:101296982-101297082 | 1.000000 | 100 |
| chr11:87755504-87755604 | 1.000000 | 100 |
| chr7:45510572-45510672 | 1.000000 | 100 |
| chr1:177259317-177259417 | 1.000000 | 100 |
| chr5:139284336-139284436 | 1.000000 | 100 |
| chr5:108707896-108707996 | 1.000000 | 100 |
| chr5:124490001-124490101 | 1.000000 | 100 |
| chr17:84926353-84926453 | 1.000000 | 100 |
| chr4:136653119-136653219 | 1.000000 | 100 |
| chr11:33863308-33863408 | 1.000000 | 100 |
| chr17:32321153-32321253 | 1.000000 | 100 |
| chr4:154191336-154191436 | 1.000000 | 100 |
| chr17:35165394-35165494 | 1.000000 | 100 |
| chr11:60486424-60486524 | 1.000000 | 100 |
| chr7:99764014-99764114 | 1.000000 | 100 |
| chr1:184669364-184669464 | 1.000000 | 100 |
| chr10:83060369-83060469 | 1.000000 | 100 |
| chr13:49263408-49263508 | 1.000000 | 100 |
| chr16:76370166-76370266 | 1.000000 | 100 |
| chr8:121438945-121439045 | 1.000000 | 100 |
| chr2:130687197-130687297 | 1.000000 | 100 |
| chr15:79690324-79690424 | 1.000000 | 100 |
| chr1:191055364-191055464 | 1.000000 | 100 |
| chr10:70133348-70133448 | 1.000000 | 100 |
| chr11:90664423-90664523 | 1.000000 | 100 |
| chr2:157006157-157006257 | 1.000000 | 100 |
| chr17:29490041-29490141 | 1.000000 | 100 |
| chr19:5445275-5445375 | 1.000000 | 100 |
| chr11:101277792-101277892 | 1.000000 | 100 |
| chr19:7552151-7552251 | 1.000000 | 100 |
| chr19:6284558-6284658 | 1.000000 | 100 |
| chr2:28032679-28032779 | 1.000000 | 100 |
| chr18:34164850-34164950 | 1.000000 | 100 |
| chr5:123985329-123985429 | 1.000000 | 100 |
| chr2:173586727-173586827 | 1.000000 | 100 |
| chr19:40977065-40977165 | 1.000000 | 100 |
| chr12:76941689-76941789 | 1.000000 | 100 |
| chr4:134924511-134924611 | 1.000000 | 100 |
| chr2:91664865-91664965 | 1.000000 | 100 |
| chr2:118660266-118660366 | 1.000000 | 100 |
| chr6:38921194-38921294 | 1.000000 | 100 |
| chr10:17596274-17596374 | 1.000000 | 100 |
| chr7:122015208-122015308 | 1.000000 | 100 |
| chr16:90144449-90144549 | 1.000000 | 100 |
| chr17:24478950-24479050 | 1.000000 | 100 |
| chr12:83073377-83073477 | 1.000000 | 100 |
| chr16:32165408-32165508 | 1.000000 | 100 |
| chr9:96254978-96255078 | 1.000000 | 100 |
| chr2:130576244-130576344 | 1.000000 | 100 |
| chr11:54720316-54720416 | 1.000000 | 100 |
| chr6:120580192-120580292 | 1.000000 | 100 |
| chr4:41129997-41130097 | 1.000000 | 100 |
| chr8:105300153-105300253 | 1.000000 | 100 |
| chr4:57116435-57116535 | 1.000000 | 100 |
| chr10:78202589-78202689 | 1.000000 | 100 |
| chr6:29583606-29583706 | 1.000000 | 100 |
| chr17:25598196-25598296 | 1.000000 | 100 |
| chr19:27429900-27430000 | 1.000000 | 100 |
| chr6:38757779-38757879 | 1.000000 | 100 |
| chr9:45803597-45803697 | 1.000000 | 100 |
| chr19:56543498-56543598 | 1.000000 | 100 |
| chr13:43191907-43192007 | 1.000000 | 100 |
| chr19:59261721-59261821 | 1.000000 | 100 |
| chr10:127126665-127126765 | 1.000000 | 100 |
| chr17:46679988-46680088 | 1.000000 | 100 |
| chr11:120303657-120303757 | 1.000000 | 100 |
| chr7:132492076-132492176 | 1.000000 | 100 |
| chr13:95965321-95965421 | 1.000000 | 100 |
| chr2:101551581-101551681 | 1.000000 | 100 |
| chr12:111518050-111518150 | 1.000000 | 100 |
| chr17:35836735-35836835 | 1.000000 | 100 |
| chr5:137102973-137103073 | 1.000000 | 100 |
| chrX:57373707-57373807 | 1.000000 | 100 |
| chr8:117032781-117032881 | 1.000000 | 100 |
| chr19:16767067-16767167 | 1.000000 | 100 |
| chr14:56968990-56969090 | 1.000000 | 100 |
| chr5:110813937-110814037 | 1.000000 | 100 |
| chr2:152654362-152654462 | 1.000000 | 100 |
| chr14:65145074-65145174 | 1.000000 | 100 |
| chr10:94264745-94264845 | 1.000000 | 100 |
| chr3:100188848-100188948 | 1.000000 | 100 |
| chr12:80790108-80790208 | 1.000000 | 100 |
| chr2:117233797-117233897 | 1.000000 | 100 |
| chr17:27393538-27393638 | 1.000000 | 100 |
| chr12:105011655-105011755 | 1.000000 | 100 |
| chr1:136062109-136062209 | 1.000000 | 100 |

hide training set...

##### Command line summary

meme ./seqs-sampled -oc meme\_out -dna -mod zoops -nmotifs 3 -minw 6 -maxw 30 -bfile ./background -time 6056 -revcomp -nostatus   
Letter frequencies in dataset  

A: 0.243   C: 0.257   G: 0.257   T: 0.243

  
Background letter frequencies (from ./background):  

A: 0.243   C: 0.257   G: 0.257   T: 0.243

##### Stopping Reason

Stopped because nmotifs = 3 reached. Program ran on *sten-0-0.local*.

show model parameters...

##### Model parameters

host = sten-0-0.local
type = zoops
nmotifs = 3
evalue\_threshold = inf
object\_function = E-value of product of p-values
min\_width = 6
max\_width = 30
minic = 0.00
wg = 11
ws = 1
endgaps = yes
minsites = 2
maxsites = 600
wnsites = 0.8
prob = 1
spmap = uni
spfuzz = 0.5
prior = dirichlet
beta = 0.01
maxiter = 50
distance = 1e-05
num\_sequences = 600
num\_positions = 60000
seed = 0
seqfrac = 1
strands = both
priors\_file =
reason\_for\_stopping = Stopped because nmotifs = 3 reached.

hide model parameters...

|  |  |
| --- | --- |
| Explanation of MEME Results | Top |

#### The MEME results consist of

- The **overview** of all discovered motifs.
- Information on each of the **motifs** MEME discovered, including:
  1. A **summary table** showing the width, number of contributing sites, log likelihood ratio,
     statistical significance, information content and relative entropy of the motif.
  2. A **sequence LOGO**.
  3. Downloadable **LOGO files** suitable for publication.
  4. A **regular expression** describing the motif.
  5. Some **further analysis** that can be performed on the motif.
  6. A list of **data formats** describing the motif.
  7. The **contributing sites** of the motif sorted by *p*-value and aligned with each other.
  8. The **block diagrams** of the contributing sites of the motif within each sequence in the training set.
- A **combined block diagram** showing an optimized (non-overlapping)
  tiling of all of the motifs onto each of the sequences in the training set.
- The **version** of MEME and the date it was released.
- The **reference** to cite if you use MEME in your research.
- A description of the **sequences** you submitted (the "training set") showing the name,
  "weight" and length of each sequence.
- The **command line summary** detailing the parameters with which you ran MEME.
- The reason why MEME **stopped** and the name of the CPU on which it ran.
- This **explanation** of how to interpret MEME results.

#### Motifs

For each motif that it discovers in the training set,
MEME prints the following information:

##### Summary Table

This summary table gives the main attributes of the motif.

*E*-value
:   The statistical significance of the motif. MEME usually finds the most statistically significant (low *E*-value) motifs first.
    The *E*-value of a motif is based on its log likelihood ratio, width, sites, the background letter frequencies (given in the
    command line summary), and the size of the training set. The *E*-value is an estimate of the expected
    number of motifs with the given log likelihood ratio (or higher), and with the same width and site count, that one would find in
    a similarly sized set of random sequences. (In random sequences each position is independent with letters chosen according to
    the background letter frequencies.)

Width
:   The width of the motif. Each motif describes a pattern of a fixed with as no gaps are allowed in MEME motifs.

Sites
:   The number of sites contributing to the construction of the motif.

Log Likelihood Ratio
:   The log likelihood ratio of the motif.The log likelihood ratio is the logarithm of the ratio of the probability of the occurrences
    of the motif given the motif model (likelihood given the motif) versus their probability given the background model
    (likelihood given the null model). (Normally the background model is a 0-order Markov model using the background letter
    frequencies, but higher order Markov models may be specified via the **-bfile** option to MEME.)

Information Content
:   The information content of the motif in bits. It is equal to the sum of the **uncorrected** information content, R(),
    in the columns of the LOGO. This is equal relative entropy of the motif relative to a uniform background frequency
    model.

Relative Entropy
:   The relative entropy of the motif, computed in bits and relative to the background letter frequencies given in the
    command line summary. It is equal to the log-likelihood ratio (llr) divided by the
    number of contributing sites of the motif times 1/ln(2),  
      
    re = llr / (sites \* ln(2)).

##### Sequence LOGO

MEME motifs are represented by position-specific probability matrices
that specify the probability of each possible letter appearing at each
possible position in an occurrence of the motif. These are displayed
as "sequence LOGOS", containing stacks of letters at each position
in the motif. The total height of the stack is the "information
content" of that position in the motif in bits. The height of the
individual letters in a stack is the probability of the letter at that
position multiplied by the total information content of the stack.

Note: The MEME LOGO differs from those produced by the
Weblogo program
because a **small-sample correction is NOT applied**.
However, MEME LOGOs in PNG and encapsulated postscript (EPS) formats
**with small-sample correction (SSC)** are available by clicking
on the download button with "SSC" set to "on" under
Download LOGO.
The MEME LOGOs without small sample correction are similarly available.
Error bars are included in the LOGOs with small-sample correction.

Modern web browsers supporting the canvas element and it's text manipulation functions as described in the
html 5 standard, can render the sequence LOGOs without needing the images. The browsers which work with this
feature are:

- Firefox 3.5 and above
- Safari 4 and above
- Google Chrome 4 and above

Unfortunately Internet Explorer 8 does not support any html 5 features.

The information content of each motif position is computed as described in the paper by Schneider and Stephens,
"Sequence Logos: A New Way to Display Consensus Sequences" but
**the small-sample correction, e(n), is set to zero for the LOGO displayed in the MEME output.**
The corrected information content of position i is given by

```
            R(i) for amino acids   = log2(20) - (H(i) + e(n))   (1a) 
            R(i) for nucleic acids =    2    - (H(i) + e(n))    (1b)
```

where H(i) is the entropy of position i,

```
            H(l) = - (Sum f(a,i) * log2[ f(a,i) ]).             (2)
```

Here, f(a,i) is the frequency of base or amino acid a at position i, and e(n) is the small-sample correction
for an alignment of n letters. The height of letter a in column i is given by

```
            height = f(a,i) * R(i)                              (3)
```

The approximation for the small-sample correction, e(n), is given by:

```
            e(n) = (s-1) / (2 * ln(2) * n),                     (4)
```

where s is 4 for nucleotides, 20 for amino acids, and n is the number of sequences in the alignment.

The letters in the logos are colored as follows.  
For DNA sequences, the letter categories contain one letter each.

| NUCLEIC ACIDS | COLOR |
| --- | --- |
| A | RED |
| C | BLUE |
| G | ORANGE |
| T | GREEN |

For proteins, the categories are based on the biochemical properties of the various amino acids.

| AMINO ACIDS | COLOR | PROPERTIES |
| --- | --- | --- |
| A, C, F, I, L, V, W and M | BLUE | Most hydrophobic[Kyte and Doolittle, 1982] |
| NQST | GREEN | Polar, non-charged, non-aliphatic residues |
| DE | MAGENTA | Acidic |
| KR | RED | Positively charged |
| H | PINK |  |
| G | ORANGE |  |
| P | YELLOW |  |
| Y | TURQUOISE |  |

J. Kyte and R. Doolittle, 1982. "A Simple Method for Displaying the Hydropathic Character of a Protein",
J. Mol Biol. 157, 105-132.

**Note:** the "text" output format of MEME preserves the historical MEME format where LOGOS are replaced by a simplified probability
matrix, a relative entropy plot, and a multi-level consensus sequence.

##### Download LOGO

Logos can be generated on the fly by the meme webservice and you may specify a number of options to customize them to your needs.
The options are:

Orientation
:   Only valid for nucleotide motifs. Generate the standard view or the reverse complemented view of the motif.

SSC
:   Use small sample correction and show errorbars on the image.
    Small sample correction is used by the Weblogo program.

Format
:   The format of the generated image.
    If the image is to be used on the web then png is recommend.
    If the image is to be published then eps is recommended.

Width
:   The width of the generated image in centimetres.

Height
:   The height of the generated image in centimetres.

##### Regular Expression

This is a regular expression (RE) describing the motif. In each column, all letters with observed frequencies greater than 0.2 are shown;
less-frequent letters are not included in the RE. MEME regular expressions are interpreted as follows: single letters match that letter;
groups of letters in square brackets match any of the letters in the group. Regular expressions can be used for searching for the motif
in sequences (using, for example, PatMatch) but the search
accuracy will usually be better with the PSSM (using, for example MAST.)

##### Further Analysis

Either as a group or individually the motifs have a number of options for further analysis.

MAST
:   Finds the best matching positions for a set of motifs in each sequence provided to it, ranked by the combined score of each sequence.
    For more information about MAST please read the introduction.

FIMO
:   Finds all matches for a motif.
    For more information about FIMO please read the introduction.

TOMTOM
:   Compares a single motif to a database of motifs.
    For more information about TOMTOM please read the introduction.

GOMO
:   Identifies possible roles of DNA binding motifs.
    For more information about GOMO please read the introduction.

BLOCKS
:   Submit to Blocks Multiple Alignment Processor where you can do several things like create phylogeny trees and search the blocks
    against a database of other blocks (protein only). For more information about BLOCKS Multiple Alignment Processor please visit the
    website.

##### Data Formats

The extracted data is avaliable in the following formats.

PSPM Format
:   The motif itself is a position-specific probability matrix giving, for each position in the pattern, the observed frequency ("probability")
    of each possible letter. The probability matrix is printed "sideways"--columns correspond to the letters in the alphabet (in the same order
    as shown in the simplified motif) and rows corresponding to the positions of the motif, position one first. The motif is preceded by a line
    starting with "letter-probability matrix:" and containing the length of the alphabet, width of the motif, number of occurrences of the motif,
    and the *E*-value of the motif.  
    **Note:** Earlier versions of MEME gave the posterior probabilities--the probability after applying a prior on letter frequencies--rather
    than the observed frequencies. These versions of MEME also gave the number of *possible* positions for the motif rather than the actual
    number of occurrences. The output from these earlier versions of MEME can be distinguished by "n=" rather than "nsites=" in the line
    preceding the matrix.

PSSM Format
:   The position-specific scoring matrix corresponding to the motif is printed for use by database search programs such as MAST.
    This matrix is a log-odds matrix calculated by taking 100 times the log (base 2) of the ratio *p/f* at each position in
    the motif where *p* is the probability of a particular letter at that position in the motif, and *f* is the background
    frequency of the letter (given in the command line summary section.) This is the same matrix that is used
    above in computing the *p*-values of the occurrences of the motif in the Sites
    and Block Diagrams sections. The scoring matrix is printed "sideways"--columns
    correspond to the letters in the alphabet (in the same order as shown in the simplified motif) and rows corresponding to the
    positions of the motif, position one first. The scoring matrix is preceded by a line starting with "log-odds matrix:" and
    containing the length of the alphabet, width of the motif, number of characters in the training set, the scoring threshold
    (obsolete) and the motif *E*-value.  
    **Note:** The probability *p* used to compute the PSSM is *not* exactly the same as the corresponding value in the
    Position Specific Probability Matrix (PSPM). The values of *p* used to compute the PSSM take into account the motif prior,
    whereas the values in the PSPM are just the *observed* frequencies of letters in the motif sites.

BLOCKS Format
:   For use with BLOCKS tools.

FASTA Format
:   The FASTA format as described here.

Raw Format
:   Just the sites of the sequences that contributed to the motif. One site per line.

##### Sites

MEME displays the occurrences (sites) of the motif in the training set. The sites are shown aligned with each other, and the ten sequence
positions preceding and following each site are also shown. Each site is identified by the name of the sequence where it occurs,
the strand (if both strands of DNA sequences are being used), and the position in the sequence where the site begins. When the DNA strand
is specified, '+' means the sequence in the training set, and '-' means the reverse complement of the training set sequence.
(For '-' strands, the 'start' position is actually the position on the **positive** strand where the site ends.) The sites are **listed
in order of increasing statistical significance** (*p*-value). The *p*-value of a site is computed from the the match score of
the site with the position specific scoring matrix for the motif. The *p*-value gives the probability of a
random string (generated from the background letter frequencies) having the same match score or higher. (This is referred to as the
**position *p*-value** by the MAST algorithm.)

##### Block Diagrams

The occurrences of the motif in the training set sequences are shown as coloured blocks on a line. One diagram is printed for each
sequence showing all the sites contributating to that motif in that sequence. The sequences are **listed in the same order as in the input**
to make it easier to compare multiple block diagrams. Additionally the best *p*-value for the sequence/motif combination is
listed though this may not be in ascending order as with the sites. The *p*-value of an occurrence is the probability of a single
random subsequence the length of the motif, generated according to the 0-order background model, having a score at least as high as
the score of the occurrence. When the DNA strand is specified '+', it means the motif appears from left to right on the sequence, and '-'
means the motif appears from right to left on the complementary strand. A sequence position scale is shown at the end of each table of
block diagrams.

##### Combined Block Diagrams

The motif occurrences shown in the motif summary **may not be exactly the same as those reported in each motif section** because
only motifs with a position *p*-value of 0.0001 that don't overlap other, more significant motif occurrences are shown.

See the documentation for MAST output for the definition of position and
combined *p*-values.
