# Supplementary material for: Altered Chromatin Occupancy of Master Regulators Underlies Evolutionary Divergence in the Transcriptional Landscape of Erythroid Differentiation
Source: PLoS Genet. 2014 Dec 18;10(12):e1004890. doi: 10.1371/journal.pgen.1004890 (PMC4270484; doi:10.1371/journal.pgen.1004890)
Supplement: S7 Dataset — Human proerythroblast KLF1 motif discovery results. Raw motif results. Abbreviations used: none. (ZIP) [file pgen.1004890.s028.zip › Dataset S7 human_KLF1.tar!/appMEMECHIP_4.9.11402016315418885606493/dreme_out/dreme.html]

DREME


The name of the motif uses the IUPAC codes for nucleotides which has
a different letter to represent each of the 15 possible combinations.

The name is itself a representation of the motif though the position
weight matrix is not directly equalivant as it is generated from the
sites found that matched the letters given in the name.

Read more about the MEME suite's use of the IUPAC alphabets.

[close ]

The logo of the motif.

[close ]

The logo of the reverse complement motif.

[close ]

The E-value is the enrichment p-value times the number of candidate
motifs tested.

The enrichment p-value is calculated using the Fisher Exact Test for
enrichment of the motif in the positive sequences.

Note that the counts used in the Fisher Exact Test are made after
erasing sites that match previously found motifs.

[close ]

The E-value of the motif calculated without erasing the sites of
previously found motifs.

[close ]

Show more information on the motif.

[close ]

Submit your motif to another MEME Suite program.

##### Supported Programs

Tomtom
:   Tomtom is a tool for searching for similar known motifs.
    [manual]

MAST
:   MAST is a tool for searching biological sequence databases for
    sequences that contain one or more of a group of known motifs.
    [manual]

FIMO
:   FIMO is a tool for searching biological sequence databases for
    sequences that contain one or more known motifs.
    [manual]

GOMO
:   GOMO is a tool for identifying possible roles (Gene Ontology
    terms) for DNA binding motifs.
    [manual]

SpaMo
:   SpaMo is a tool for inferring possible transcription factor
    complexes by finding motifs with enriched spacings.
    [manual]

[close ]

Download your motif as a position weight matrix or a custom logo.

[close ]

# positive sequences matching the motif / # positive sequences.

Note these counts are made after erasing sites that match previously
found motifs.

[close ]

# negative sequences matching the motif / # negative sequences.

Note these counts are made after erasing sites that match previously
found motifs.

[close ]

The p-value of the Fisher Exact Test for enrichment of the motif in
the positive sequences.

Note that the counts used in the Fisher Exact Test are made after
erasing sites that match previously found motifs.

[close ]

The E-value is the motif p-value times the number of candidate motifs
tested.

Note that the p-value was calculated with counts made after
erasing sites that match previously found motifs.

[close ]

The E-value of the motif calculated without erasing the sites of
previously found motifs.

[close ]

All words matching the motif whose uncorrected p-value is less than
0.01.

[close ]

# positive sequences with matches to the word / # positive sequences.

Note these counts are made after erasing sites that match previously
found motifs.

[close ]

# negative sequences with matches to the word / # negative sequences.

Note these counts are made after erasing sites that match previously
found motifs.

[close ]

The p-value of the Fisher Exact Test for enrichment of the word in
the positive sequences.

Note that the counts used in the Fisher Exact Test are made after
erasing sites that match previously found motifs.

[close ]

The word p-value times the number of candidates tested.

Note that the p-value was calculated with counts made after
erasing sites that match previously found motifs.

[close ]

## .

↥

Submit

⇢

Download

⟱

#### Details

| Positives ? | Negatives ? | P-value ? | E-value ? | Unerased E-value ? |
| --- | --- | --- | --- | --- |
| /48429 | /48429 |  |  |  |

#### Enriched Matching Words

| Word ? | Positives ? | Negatives ? | P-value ? | ▼E-value ? |
| --- | --- | --- | --- | --- |
|  | /48429 | /48429 |  |  |

## Submit ""

## Submit All Motifs

x

⇧

⇩

#### Select what you want to do

- Search Motifs
- Search Sequences
- Rank Sequences
- Predict Gene Ontology terms
- Infer TF Complexes

#### Select a program

- Tomtom
- FIMO
- MAST
- GOMO
- SpaMo

Or

#### Search Motifs with Tomtom

Find similar motifs in published
libraries or a library you supply.

## Download ""

x

⇧

⇩

PSPM FormatPSSM FormatLogo

|  |  |
| --- | --- |
| Format: | PNG (for web) EPS (for publication) |
| Orientation: | Normal Reverse Complement |
| Small Sample Correction: | Off On |
| Width: | cm |
| Height: | cm |

For further information on how to interpret these results or to get a
copy of the MEME software please access
http://meme.nbcr.net.

If you use DREME in your research please cite the following paper:  
Timothy L. Bailey, "DREME: Motif discovery in transcription factor ChIP-seq data", *Bioinformatics*, **27**(12):1653-1659, 2011.

Discovered motifs  |  Program information

|  |  |
| --- | --- |
| Discovered Motifs | Next Top |

**Click on the ↧** under the **More** column to show more
information about the motif.  
**Click on the ⇢** under the **Submit** column to send the
motif to another MEME suite program. Eg. Tomtom  
**Click on the ⟱** under the **Download** column to get
the position weight matrix of a motif or to download the logo image with
your chosen options.

|  | Motif ? | Logo ? | RC Logo ? | E-value ? | Unerased E-value ? | More ? | Submit ? | Download ? |
| --- | --- | --- | --- | --- | --- | --- | --- | --- |
| 1. | VGGAAR |  |  | 1.6e-725 | 1.6e-725 | ↧ | ⇢ | ⟱ |
| 2. | TTATCW |  |  | 9.1e-611 | 1.4e-630 | ↧ | ⇢ | ⟱ |
| 3. | AGRDGGCR |  |  | 3.8e-901 | 4.2e-934 | ↧ | ⇢ | ⟱ |
| 4. | CYCCDCCC |  |  | 4.6e-397 | 1.7e-466 | ↧ | ⇢ | ⟱ |
| 5. | TGAGTCAB |  |  | 1.3e-288 | 9.9e-298 | ↧ | ⇢ | ⟱ |
| 6. | GGGYGTGK |  |  | 1.4e-168 | 4.2e-192 | ↧ | ⇢ | ⟱ |
| 7. | WAACCACR |  |  | 2.9e-150 | 1.9e-176 | ↧ | ⇢ | ⟱ |
| 8. | CACGTG |  |  | 2.1e-116 | 6.1e-123 | ↧ | ⇢ | ⟱ |
| 9. | GGMGGHR |  |  | 6.8e-110 | 3.3e-365 | ↧ | ⇢ | ⟱ |
| 10. | RGRAA |  |  | 2.1e-091 | 8.0e-562 | ↧ | ⇢ | ⟱ |
| 11. | GGMRGGA |  |  | 4.3e-068 | 3.8e-307 | ↧ | ⇢ | ⟱ |
| 12. | CCACTAGR |  |  | 1.2e-063 | 9.7e-169 | ↧ | ⇢ | ⟱ |
| 13. | TGACSWCA |  |  | 1.2e-058 | 2.6e-071 | ↧ | ⇢ | ⟱ |
| 14. | ARATA |  |  | 2.9e-050 | 6.1e-286 | ↧ | ⇢ | ⟱ |
| 15. | GAKGACR |  |  | 6.2e-044 | 2.2e-064 | ↧ | ⇢ | ⟱ |
| 16. | TGATGHAA |  |  | 2.6e-043 | 2.8e-046 | ↧ | ⇢ | ⟱ |
| 17. | CAKCCTS |  |  | 1.1e-036 | 3.2e-072 | ↧ | ⇢ | ⟱ |
| 18. | GCAGCNGC |  |  | 1.1e-031 | 6.0e-059 | ↧ | ⇢ | ⟱ |
| 19. | GTRACD |  |  | 6.0e-035 | 3.7e-124 | ↧ | ⇢ | ⟱ |
| 20. | GATWA |  |  | 1.6e-025 | 1.5e-430 | ↧ | ⇢ | ⟱ |
|  |  |  |  |  |  |  |  |  |
| --- | --- | --- | --- | --- | --- | --- | --- | --- |
|  |  |  |  |  |  |  |  |  |

Submit All

⇢

Previous Top

##### DREME version

4.9.1 (Release date: Fri Aug 23 16:49:42 2013 +1000)

##### Reference

Timothy L. Bailey, "DREME: Motif discovery in transcription factor ChIP-seq data", *Bioinformatics*, **27**(12):1653-1659, 2011.

##### Command line summary

dreme -v 1 -oc dreme\_out -p ./seqs-centered -n ./seqs-shuffled -png -t 8537 -e 0.05  
Result calculation took 2 hours 22 minutes 16.28 seconds

show model parameters...

##### Model parameters

positives = name: "seqs-centered", count: "48429", file: "./seqs-centered", last\_mod\_date: "Thu Jun 05 17:58:41 PDT 2014"
negatives = name: "seqs-shuffled", count: "48429", from: "file", file: "./seqs-shuffled", last\_mod\_date: "Thu Jun 05 17:59:28 PDT 2014"
background = type: "dna", A: "0.206", C: "0.293", G: "0.294", T: "0.207", from: "dataset"
stop = evalue: "0.05", time: "8537"
norc = FALSE
ngen = 100
add\_pv\_thresh = 0.01
seed = 1
host = sten-0-5.local
when = Thu Jun 05 18:20:34 PDT 2014

hide model parameters...
