# Supplementary material for: Altered Chromatin Occupancy of Master Regulators Underlies Evolutionary Divergence in the Transcriptional Landscape of Erythroid Differentiation
Source: PLoS Genet. 2014 Dec 18;10(12):e1004890. doi: 10.1371/journal.pgen.1004890 (PMC4270484; doi:10.1371/journal.pgen.1004890)
Supplement: S8 Dataset — Human proerythroblast GATA1 motif discovery results. Raw motif results. Abbreviations used: none. (ZIP) [file pgen.1004890.s029.zip › Dataset S8 human_GATA1.tar!/appMEMECHIP_4.9.11402016304829-631144661/meme_tomtom_out/tomtom.html]

TOMTOM


For further information on how to interpret these results or to get a
copy of the MEME software please access
http://meme.nbcr.net.

If you use TOMTOM in your research, please cite the following paper:  
Shobhit Gupta, JA Stamatoyannopolous, Timothy Bailey and William Stafford Noble,
"Quantifying similarity between motifs",
*Genome Biology*, **8**(2):R24, 2007.

Query Motifs  |  Target Databases  |  Matches  |  Program information  |  Explanation

|  |  |
| --- | --- |
| Query Motifs | Next Top |

| Name | Preview | Matches | List |
| --- | --- | --- | --- |
| 1 | ACAGATAAGAA | 8 | MA0035.3 (Gata1),  MA0036.2 (GATA2),  MA0482.1 (Gata4),  UP00080\_1 (Gata5\_primary),  MA0037.2 (GATA3),  UP00032\_1 (Gata3\_primary),  UP00100\_1 (Gata6\_primary),  MA0140.2 (TAL1::GATA1) |
| 2 | GCGTGAGCCACCGTGCCCGGC | 4 | MA0162.2 (EGR1),  MA0477.1 (FOSL1),  MA0066.1 (PPARG),  UP00004\_2 (Sox14\_secondary) |
| 3 | ACCCAGGCTGGGGCGCAGGGG | 9 | MA0162.2 (EGR1),  UP00022\_1 (Zfp740\_primary),  UP00021\_1 (Zfp281\_primary),  UP00006\_1 (Zic3\_primary),  UP00102\_1 (Zic1\_primary),  UP00033\_2 (Zfp410\_secondary),  MA0516.1 (SP2),  UP00057\_1 (Zic2\_primary),  UP00007\_1 (Egr1\_primary) |

|  |  |
| --- | --- |
| Target Databases | Previous Next Top |

| Database | Number of Motifs | Motifs Matched |
| --- | --- | --- |
| JASPAR\_CORE\_2014\_vertebrates.meme | 205 | 9 |
| uniprobe\_mouse.meme | 386 | 11 |

|  |  |
| --- | --- |
| Matches to Query: 1 | Previous Next Top |

|  |  |
| --- | --- |
| Summary | Alignment |
| Name \| MA0035.3; Alt. Name \| Gata1; Database \| JASPAR\_CORE\_2014\_vertebrates.meme; *p*-value \| 6.11963e-12; *E*-value \| 3.6167e-09; *q*-value \| 7.22055e-09; Overlap \| 11; Offset \| 0; Orientation \| Reverse Complement |  |
| Create custom LOGO ↧ | [Next Match] [Query Top] |
| Summary | Alignment |
| Name \| MA0036.2; Alt. Name \| GATA2; Database \| JASPAR\_CORE\_2014\_vertebrates.meme; *p*-value \| 2.44785e-11; *E*-value \| 1.44668e-08; *q*-value \| 1.44411e-08; Overlap \| 11; Offset \| 0; Orientation \| Reverse Complement |  |
| Create custom LOGO ↧ | [Previous Match] [Next Match] [Query Top] |
| Summary | Alignment |
| Name \| MA0482.1; Alt. Name \| Gata4; Database \| JASPAR\_CORE\_2014\_vertebrates.meme; *p*-value \| 1.42181e-07; *E*-value \| 8.40291e-05; *q*-value \| 5.59199e-05; Overlap \| 10; Offset \| 1; Orientation \| Reverse Complement |  |
| Create custom LOGO ↧ | [Previous Match] [Next Match] [Query Top] |
| Summary | Alignment |
| Name \| UP00080\_1; Alt. Name \| Gata5\_primary; Database \| uniprobe\_mouse.meme; *p*-value \| 1.62293e-06; *E*-value \| 0.000959151; *q*-value \| 0.000478723; Overlap \| 11; Offset \| 3; Orientation \| Normal |  |
| Create custom LOGO ↧ | [Previous Match] [Next Match] [Query Top] |
| Summary | Alignment |
| Name \| MA0037.2; Alt. Name \| GATA3; Database \| JASPAR\_CORE\_2014\_vertebrates.meme; *p*-value \| 4.68162e-06; *E*-value \| 0.00276684; *q*-value \| 0.00110477; Overlap \| 8; Offset \| -2; Orientation \| Normal |  |
| Create custom LOGO ↧ | [Previous Match] [Next Match] [Query Top] |
| Summary | Alignment |
| Name \| UP00032\_1; Alt. Name \| Gata3\_primary; Database \| uniprobe\_mouse.meme; *p*-value \| 3.08084e-05; *E*-value \| 0.0182078; *q*-value \| 0.00605847; Overlap \| 11; Offset \| 5; Orientation \| Normal |  |
| Create custom LOGO ↧ | [Previous Match] [Next Match] [Query Top] |
| Summary | Alignment |
| Name \| UP00100\_1; Alt. Name \| Gata6\_primary; Database \| uniprobe\_mouse.meme; *p*-value \| 0.000310219; *E*-value \| 0.18334; *q*-value \| 0.0522897; Overlap \| 11; Offset \| 3; Orientation \| Normal |  |
| Create custom LOGO ↧ | [Previous Match] [Next Match] [Query Top] |
| Summary | Alignment |
| Name \| MA0140.2; Alt. Name \| TAL1::GATA1; Database \| JASPAR\_CORE\_2014\_vertebrates.meme; *p*-value \| 0.000660504; *E*-value \| 0.390358; *q*-value \| 0.097416; Overlap \| 9; Offset \| 9; Orientation \| Reverse Complement |  |
| Create custom LOGO ↧ | [Previous Match] [Query Top] |

|  |  |
| --- | --- |
| Matches to Query: 2 | Previous Next Top |

|  |  |
| --- | --- |
| Summary | Alignment |
| Name \| MA0162.2; Alt. Name \| EGR1; Database \| JASPAR\_CORE\_2014\_vertebrates.meme; *p*-value \| 0.000158882; *E*-value \| 0.0938993; *q*-value \| 0.185174; Overlap \| 14; Offset \| -7; Orientation \| Normal |  |
| Create custom LOGO ↧ | [Next Match] [Query Top] |
| Summary | Alignment |
| Name \| MA0477.1; Alt. Name \| FOSL1; Database \| JASPAR\_CORE\_2014\_vertebrates.meme; *p*-value \| 0.000593475; *E*-value \| 0.350744; *q*-value \| 0.319798; Overlap \| 11; Offset \| -1; Orientation \| Reverse Complement |  |
| Create custom LOGO ↧ | [Previous Match] [Next Match] [Query Top] |
| Summary | Alignment |
| Name \| MA0066.1; Alt. Name \| PPARG; Database \| JASPAR\_CORE\_2014\_vertebrates.meme; *p*-value \| 0.000823173; *E*-value \| 0.486495; *q*-value \| 0.319798; Overlap \| 19; Offset \| -2; Orientation \| Normal |  |
| Create custom LOGO ↧ | [Previous Match] [Next Match] [Query Top] |
| Summary | Alignment |
| Name \| UP00004\_2; Alt. Name \| Sox14\_secondary; Database \| uniprobe\_mouse.meme; *p*-value \| 0.00138641; *E*-value \| 0.819366; *q*-value \| 0.323166; Overlap \| 15; Offset \| -4; Orientation \| Reverse Complement |  |
| Create custom LOGO ↧ | [Previous Match] [Query Top] |

|  |  |
| --- | --- |
| Matches to Query: 3 | Previous Next Top |

|  |  |
| --- | --- |
| Summary | Alignment |
| Name \| MA0162.2; Alt. Name \| EGR1; Database \| JASPAR\_CORE\_2014\_vertebrates.meme; *p*-value \| 5.32797e-06; *E*-value \| 0.00314883; *q*-value \| 0.00621058; Overlap \| 14; Offset \| -5; Orientation \| Reverse Complement |  |
| Create custom LOGO ↧ | [Next Match] [Query Top] |
| Summary | Alignment |
| Name \| UP00022\_1; Alt. Name \| Zfp740\_primary; Database \| uniprobe\_mouse.meme; *p*-value \| 0.000637858; *E*-value \| 0.376974; *q*-value \| 0.170887; Overlap \| 16; Offset \| -3; Orientation \| Reverse Complement |  |
| Create custom LOGO ↧ | [Previous Match] [Next Match] [Query Top] |
| Summary | Alignment |
| Name \| UP00021\_1; Alt. Name \| Zfp281\_primary; Database \| uniprobe\_mouse.meme; *p*-value \| 0.000770676; *E*-value \| 0.455469; *q*-value \| 0.170887; Overlap \| 15; Offset \| -6; Orientation \| Reverse Complement |  |
| Create custom LOGO ↧ | [Previous Match] [Next Match] [Query Top] |
| Summary | Alignment |
| Name \| UP00006\_1; Alt. Name \| Zic3\_primary; Database \| uniprobe\_mouse.meme; *p*-value \| 0.000823629; *E*-value \| 0.486765; *q*-value \| 0.170887; Overlap \| 15; Offset \| 0; Orientation \| Reverse Complement |  |
| Create custom LOGO ↧ | [Previous Match] [Next Match] [Query Top] |
| Summary | Alignment |
| Name \| UP00102\_1; Alt. Name \| Zic1\_primary; Database \| uniprobe\_mouse.meme; *p*-value \| 0.000868982; *E*-value \| 0.513568; *q*-value \| 0.170887; Overlap \| 13; Offset \| 1; Orientation \| Normal |  |
| Create custom LOGO ↧ | [Previous Match] [Next Match] [Query Top] |
| Summary | Alignment |
| Name \| UP00033\_2; Alt. Name \| Zfp410\_secondary; Database \| uniprobe\_mouse.meme; *p*-value \| 0.000881486; *E*-value \| 0.520958; *q*-value \| 0.170887; Overlap \| 17; Offset \| -4; Orientation \| Reverse Complement |  |
| Create custom LOGO ↧ | [Previous Match] [Next Match] [Query Top] |
| Summary | Alignment |
| Name \| MA0516.1; Alt. Name \| SP2; Database \| JASPAR\_CORE\_2014\_vertebrates.meme; *p*-value \| 0.00114347; *E*-value \| 0.675792; *q*-value \| 0.170887; Overlap \| 15; Offset \| -4; Orientation \| Reverse Complement |  |
| Create custom LOGO ↧ | [Previous Match] [Next Match] [Query Top] |
| Summary | Alignment |
| Name \| UP00057\_1; Alt. Name \| Zic2\_primary; Database \| uniprobe\_mouse.meme; *p*-value \| 0.0014794; *E*-value \| 0.874325; *q*-value \| 0.170887; Overlap \| 15; Offset \| 0; Orientation \| Reverse Complement |  |
| Create custom LOGO ↧ | [Previous Match] [Next Match] [Query Top] |
| Summary | Alignment |
| Name \| UP00007\_1; Alt. Name \| Egr1\_primary; Database \| uniprobe\_mouse.meme; *p*-value \| 0.00149755; *E*-value \| 0.885055; *q*-value \| 0.170887; Overlap \| 14; Offset \| -3; Orientation \| Reverse Complement |  |
| Create custom LOGO ↧ | [Previous Match] [Query Top] |

Previous Next Top

##### TOMTOM version

4.9.1 (Release date: Fri Aug 23 16:49:42 2013 +1000)

##### Reference

Shobhit Gupta, JA Stamatoyannopolous, Timothy Bailey and William Stafford Noble,
"Quantifying similarity between motifs",
*Genome Biology*, **8**(2):R24, 2007.

##### Command line summary

tomtom -verbosity 1 -oc meme\_tomtom\_out -min-overlap 5 -dist pearson -evalue -thresh 1 -no-ssc -bfile ./background meme\_out/meme.xml db/JASPAR\_CORE\_2014\_vertebrates.meme db/uniprobe\_mouse.meme  
Background letter frequencies (from ./background):  

A: 0.258   C: 0.242   G: 0.242   T: 0.258

  
Result calculation took 14.162 seconds

show model parameters...

##### Model parameters

distance\_measure = value: "pearson"
threshold = type: "evalue"
background = from: "file", A: "0.2576", C: "0.2424", G: "0.2424", T: "0.2576", file: "./background"
host = sten-0-1.local
when = Thu Jun 5 20:52:36 2014

hide model parameters...

|  |  |
| --- | --- |
| Explanation of TOMTOM Results | Previous Top |

#### The TOMTOM results consist of

- The query motifs with links to further information on matches. [View]
- The target databases with information on the number of matches. [View]
- The **matches** between query motifs and target motifs. [View]
  - The summary of match information.
  - An alignment of the two motifs.
- The **program** details including:
  1. The **version** of TOMTOM and the date it was released. [View]
  2. The **reference** to cite if you use TOMTOM in your research. [View]
  3. The **command line summary** detailing the parameters with which you ran TOMTOM. [View]
- This **explanation** of how to interpret TOMTOM results.

#### Inputs

The important inputs to TOMTOM.

##### Query Motifs

The query motifs section lists the motifs that were searched for in the target databases as well as links to matches found.

Name
:   The motif name.

Alt. Name
:   The alternative motif name. This column may be hidden if no query motifs have an alternate name.

Website
:   A link to a website which has more information on the motif. This column may be hidden if no query motifs have website information.

Preview
:   The motif preview. On supporting browsers this will display as a motif logo, otherwise the consensus sequence will be displayed.

Matches
:   The number of significant matches.

List
:   Links to the first 20 matches

##### Target Databases

The target motifs section lists details on the motif databases that were specifed to search.

Database
:   The database name.

Number of Motifs
:   The number of motifs read from the motif database minus the number that had to be discarded due to conflicting ids.

Motifs Matched
:   The number of motifs that had a match with at least one of the query motifs.

#### Matches

The matches section list the group of motifs that match each query motif. For each query-target match the following information is given:

##### Summary Table

The summary gives the important statistics about the motif match.

Name
:   The name of the matched motif.

Alt. Name
:   The alternative name of the matched motif.

*p*-value
:   The probability that the match occurred by random chance according to the null model.

*E*-value
:   The expected number of false positives in the matches up to this point.

*q*-value
:   The minimum **F**alse **D**iscovery **R**ate required to include the match.

Overlap
:   The number of letters that overlaped in the optimal alignment.

Offset
:   The offset of the query motif to the matched motif in the optimal alignment.

Orientation
:   The orientation of the matched motif that gave the optimal alignment.
    A value of "normal" means that the matched motif is as it appears in
    the database otherwise the matched motif has been reverse complemented.

##### Alignment

The image shows the alignment of the two motifs. The matched motif is shown on the top and the query motif is shown on the bottom.

##### Create custom LOGO

By clicking the link "Create custom LOGO ↧" a form to make custom logos
will be displayed. The download button can then be clicked to generate a motif
matching the selected specifications.

Image Type
:   Two image formats, png and eps, are avaliable. The pixel based portable network graphic (png) format is commonly used on the Internet
    and the Encapsulated PostScript (eps) format is more suitable for publications that might require scaling.

Error bars
:   Toggle error bars indicating the confidence of a motif based on the number of sites used in its creation.

SSC
:   Toggle adding pseudocounts for **S**mall **S**ample **C**orrection.

Flip
:   Toggle a full reverse complement of the alignment.

Width
:   Specify the width of the generated motif.

Height
:   Specify the height of the generated motif.
